# *Dact* genes are chordate specific regulators at the intersection of Wnt and Tgf-β signaling pathways

**DOI:** 10.1186/1471-2148-14-157

**Published:** 2014-08-06

**Authors:** Frank Richard Schubert, Débora Rodrigues Sobreira, Ricardo Guerreiro Janousek, Lúcia Elvira Alvares, Susanne Dietrich

**Affiliations:** 1 Institute of Biomedical and Biomolecular Sciences, School of Biological Sciences, University of Portsmouth, King Henry Building, King Henry I Street, Portsmouth PO1 2DY, UK; 2 Department of Histology and Embryology, State University of Campinas - UNICAMP, Rua Charles Darwin, s/n°, CP 6109, Campinas, SP CEP 13083-863, Brazil; 3 Institute of Biomedical and Biomolecular Sciences, School of Pharmacy and Biomedical Sciences, University of Portsmouth, St. Michael’s Building, White Swan Road, Portsmouth PO1 2DT, UK

**Keywords:** Dact, Dapper, Frodo, Wnt signaling, Tgfβ signaling, Evolution, Protein motifs, Expression, Chordates, Vertebrates

## Abstract

**Background:**

Dacts are multi-domain adaptor proteins. They have been implicated in Wnt and Tgfβ signaling and serve as a nodal point in regulating many cellular activities. *Dact* genes have so far only been identified in bony vertebrates. Also, the number of *Dact* genes in a given species, the number and roles of protein motifs and functional domains, and the overlap of gene expression domains are all not clear. To address these problems, we have taken an evolutionary approach, screening for *Dact* genes in the animal kingdom and establishing their phylogeny and the synteny of *Dact* loci. Furthermore, we performed a deep analysis of the various Dact protein motifs and compared the expression patterns of different *Dacts*.

**Results:**

Our study identified previously not recognized *dact* genes and showed that they evolved late in the deuterostome lineage. In gnathostomes, four *Dact* genes were generated by the two rounds of whole genome duplication in the vertebrate ancestor, with *Dact1/3* and *Dact2/4*, respectively, arising from the two genes generated during the first genome duplication. In actinopterygians, a further *dact4r* gene arose from retrotranscription. The third genome duplication in the teleost ancestor, and subsequent gene loss in most gnathostome lineages left extant species with a subset of *Dact* genes. The distribution of functional domains suggests that the ancestral Dact function lied with Wnt signaling, and a role in Tgfβ signaling may have emerged with the Dact2/4 ancestor. Motif reduction, in particular in Dact4, suggests that this protein may counteract the function of the other Dacts. *Dact* genes were expressed in both distinct and overlapping domains, suggesting possible combinatorial function.

**Conclusions:**

The gnathostome *Dact* gene family comprises four members, derived from a chordate-specific ancestor. The ability to control Wnt signaling seems to be part of the ancestral repertoire of Dact functions, while the ability to inhibit Tgfβ signaling and to carry out specialized, ortholog-specific roles may have evolved later. The complement of *Dact* genes coexpressed in a tissue provides a complex way to fine-tune Wnt and Tgfβ signaling. Our work provides the basis for future structural and functional studies aimed at unraveling intracellular regulatory networks.

## Background

Wingless and Transforming growth factor beta (Tgfβ) signaling are two cell-cell signaling systems that are well conserved throughout the animal kingdom and that control a plethora of processes ranging from embryonic development, cell proliferation, differentiation and migration, to tissue homeostasis, stem cell behavior, tissue regeneration and cancer [1,2]. Dact (Dapper/Frodo) proteins have been identified in mammals, chicken, frog and zebrafish as intracellular multi-adapter molecules with the ability to modulate and possibly integrate the Wnt and Tgfβ signaling cascades. This ability primarily relies on the physical interaction of Dact proteins with Dvl (Dishevelled), CKIδ/ϵ, Vangl, PKA, PKC, which are players in the various Wnt pathways, or with the Alk4/5 Tgfβ receptors [3-9]. In line with these properties, Dact proteins positively as well as negatively regulate the Wnt/β-Catenin pathway and positively regulate the Wnt/PCP pathway (involvement in the Wnt/Ca^2+^ pathway has not been investigated). In addition, specifically Dact2 has been implicated in the suppression of Tgfβ-dependent wound healing and Nodal-dependent mesoderm induction due to its ability to facilitate lysosomal degradation of Alk5 [6,7,10]. In addition to these established roles, Dact proteins have been shown to stabilize p120 Catenin (a mediator of Cadherin function and Rho GTPases) which in turn sequesters the transcriptional repressor Kaiso, thus leading to the activation of Kaiso targets [11]. Since the p120-Dact interaction is stimulated by Wnt and is mediated by Dvl, and because many Kaiso targets are also Tcf/Lef targets, the p120 Catenin/Kaiso pathway is seen as a parallel pathway to the Wnt/β-Catenin pathway. Dact proteins have been shown to also modulate Wnt signaling mediators in a ligand-independent fashion: Dact proteins shuttle between the nucleus and cytoplasm, and can block nuclear β-Catenin function by disrupting β-Catenin/Lef1 complexes and enhancing Lef1-HDAC interaction [12]. However, they can also promote Tcf/Lef function when the Dact N-terminal domain interacts with these transcription factors [13]. In addition, Dact proteins can interact with Dbf4 which, independent from its role in cell cycle regulation, inhibits β-Catenin targets [14]. Finally, Dact function has been shown to depend on its phosphorylation state which is controlled in two ways: firstly, in the absence of Wnt, Dact is unphosphorylated, binds to Dvl and blocks its ability to protect β-Catenin from phosphorylation, thus promoting β-Catenin degradation. In the presence of Wnt, CKIδ/ϵ not only phosphorylates Dvl but also Dact; this decreases their affinity and promotes the resolution of β-Catenin destruction complex, thereby stabilizing β-Catenin. It also allows Dact to promote the function of Tcf/Lef molecules, thus further enhancing the Wnt response [15]. Secondly, cyclic AMP activated PKA phosphorylates Dact; this allows the binding of 14-3-3β which also blocks the ability of Dact to promote Dvl degradation, thus enhancing Wnt signal transduction [16]. Taken together, Dact proteins have emerged as nodal points in the simultaneous control of the various Wnt and Tgfβ signaling pathways.

Dact are modular proteins, using different structural domains to interact with their specific partners. The functions of some of these domains have already been characterized. A leucine zipper located in the N-terminal half of the protein is required for homo-and hetero-dimerization, a C-terminal PDZ binding domain together with a domain located in the center of the protein is crucial for Dvl binding, a serine-rich domain upstream of the PDZ binding domain is required for the interaction with Vangl2, the sequences encoded by the first three and the start of the fourth exon are sufficient to inhibit Alk5, a region encoded by the end of the 3^rd^ and start of the 4^th^ exon has been implicated in Tcf3 binding and a not well characterized central portion of the protein interacts with p120 Catenin [3-9,11,13,17]. Furthermore, nuclear export and import signals have been identified [12]. However, *in vitro* binding studies showed that binding affinity and specificity of Dact proteins with their various partners is variable, with mouse Dact2 being the only Dact showing significant affinity to Tcf/Lef and Alk5 and, in comparison to Dact1 and Dact3, weak binding to Vangl2 [9]. Knock out studies in the mouse implicated Dact1 in Wnt/PCP and Dact2 in Tgfβ signaling, yet morpholino knockdown experiments in zebrafish implicated dact1 in Wnt/β-Catenin and dact2 in Wnt/PCP signaling [8,10,18]. This indicates that the structure-function relationship of Dact proteins is still unclear.

A key factor in our limited understanding of Dact function is the fact that the full complement of *Dact* genes available in different animals to regulate Wnt and Tgfβ signaling is not known, and therefore, *Dact* functions may have been overlooked or misinterpreted due to gene redundancy. Moreover, *Dact* genes have so far only been found in bony vertebrates. However, bony vertebrates together with cartilaginous vertebrates belong to the infraphylum of jawed vertebrates, and in the ancestors of this animal group the genome has been duplicated twice, followed by subsequent gene loss or gene diversification [19-23]. Thus, the origin of *Dact* genes and their evolutionarily basic function is not known. To unravel the original and derived roles of *Dact* genes and proteins, we took an evolutionary approach. We searched for so far elusive *Dact* family members in the animal kingdom, and, using bioinformatic tools, we determined their phylogeny. Moreover, we searched for conserved amino acid stretches that may serve as functional domains. Finally, we determined the expression of *dact* genes in the zebrafish, the organism with the highest number of *dact* genes, in comparison with that of the chicken, which has only two.

Our study shows that *Dact* genes are unique to chordates. In jawed vertebrates, four distinct *Dact* paralogs were identified, with *Dact1* and *Dact3* originating from one, *Dact2* and *Dact4* from the second *Dact* gene that was present after 1R. Remarkably, all four genes are still present in *Latimeria* (a lobe-finned animal related to tetrapods), turtles (anapsid reptiles) as well as lizards and snakes (diapsid reptiles), but mammals, birds and amphibians have independently lost particular *Dact* genes. In most teleosts, a *dact1*, *dact2*, two *dact3* and one *dact4* gene have been kept; zebrafish and the spotted gar, a holost fish, have an additional, intronless and hence possibly retrotranscribed *dact4r*. Motif comparison suggests that the ability to dimerize, shuttle between cytoplasm and nucleus, bind Tcf/Lef and Vangl molecules and to interact with various kinases may have been already present in the ancestral Dact protein. The ability to interact with Alk5 may have evolved with Dact2 and 4. Motif combinations in extant Dact4 proteins suggest that these molecules may sequester Dact binding partners, thereby inhibiting their function. Significantly, the various *Dact* genes show similar expression patterns, suggesting that in a given tissue, the regulation of Wnt and Tgfβ signaling will depend on the combinatorial action of Dact proteins.

## Results

### Searches for Dact genes in the animal kingdom

#### Identification of new members of the gnathostomeDact gene family

**Figure 1:**
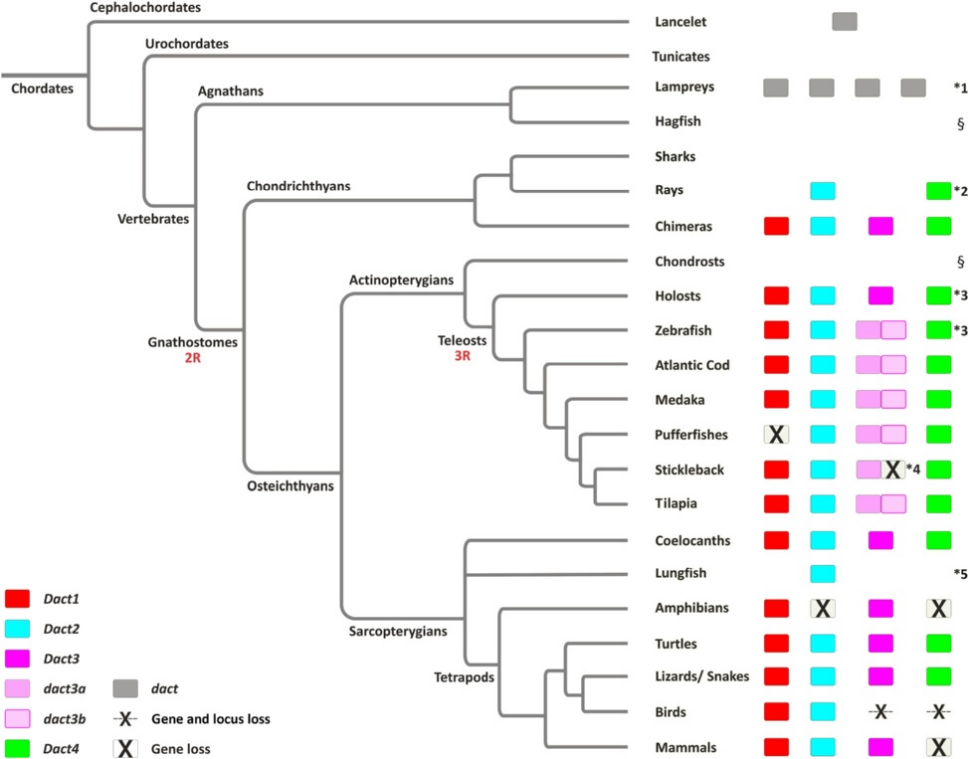
***Dact *genes are a chordate innovation.** Diagram showing the *Dact* genes found in the database searches of the main chordate taxa. The gene family is exclusive to gnathostome and cyclostome vertebrates and cephalochordates, suggesting that these genes arose in the deuterostome lineage when chordates evolved. In the lancelet only one *dact* gene was found, while at least four different *dact* genes (*dactA-D*) were recognized in cyclostomes. Gnathostome *Dact* genes fell into four distinct paralog groups (*Dact1-4*), resulting from two rounds of genome duplications (1R, 2R). Teleost fish showed two *dact3*-related paralogs, the only duplicates generated by 3R that have been maintained in extant species. An intronless *dact4*-related gene was revealed in the gar and the zebrafish, probably having been retrotranscribed in the actinopterygian lineage before the holost-teleost split. Interestingly, the four gnathostome *Dact* groups are distinct from the four cyclostome *dact* genes, suggesting independent duplications in the cyclostome and gnathostome lineage. *Dact* genes identified in this study are represented by filled boxes; gene loss is indicated by a crossed box, gene locus loss by a singular cross. For the sake of simplicity, the loss of genes or loci generated during teleost 3R is not indicated. § No sequence information available. *1 Four distinct sets of dact sequences and additional short sequence stretches. *2 Insufficient sequence information to allow a conclusive analysis of the number of paralogs. *3 Gar and zebrafish have a second, intronless *dact4* gene that possibly was retrotranscribed. *4 Large sequence gaps on the stickleback chromosome carrying the *dact3b* locus. *5Insufficient sequence information for lungfish; however a *Dact2* gene was found for *Protopterus aethiopicus*.

Currently, three *Dact* family members are known in mouse and humans, two *Dact* genes have been identified in the chicken, one in *Xenopus* (with a *dact1a* and *dact1b/ frodo* gene in the pseudo-tetraploid *Xenopus laevis*) and two in the zebrafish [3,4,24-28]. In order to obtain a comprehensive overview of *Dact* genes in jawed vertebrates (gnathostomes), we searched the genomes of various lobe-finned/lobe-limbed (=sarcopterygian) and ray-finned (actinopterygian) bony vertebrates. In our search we also included the genomic database for the elephant shark, a cartilaginous (=chondrichthyan) vertebrate. To perform these searches, we interrogated the Ensembl and NCBI databases using the known human, mouse, chicken, *Xenopus laevis* and zebrafish Dact protein sequences as queries. Moreover, we performed searches with protein sequences encoded by individual exons or we used known Dact protein motifs. Since some of the selected genomes are not fully characterized, we also used the query sequences to interrogate the NCBI expressed sequence tags (EST) database for the aforementioned groups, for additional bony vertebrates and for the spiny dogfish shark, Pacific electric ray and little skate (chondrichthyan vertebrates). The organisms searched in this study are listed in Additional file 1; the accession numbers of sequences are provided in Additional file 2, the results of our searches are shown in Figure 1.

The searches revealed that like mouse and humans, all mammals carried three *Dact* genes and all birds had two. In amphibians, we discovered a previously not recognized *dact* gene, increasing the complement of *Dact* genes in these animals to two as well. Remarkably, four distinct *Dact* genes were found in lizards and snakes, in turtles and in the coelacanth, while five *dact* genes were present in the gar as well as in the Tilapia, Medaka and the Atlantic cod, six in zebrafish, four in the stickleback and in pufferfish. These newly discovered genes indicate that the gnathostome *Dact* gene family is larger than previously anticipated. In order to ensure that all gnathostome Dact family members were traced, we repeated the searches, using the newly discovered sequences as queries. These searches, however, did not produce any further hits and confirmed the earlier results.

Based on similarities in sequence and organization, the *Dact* genes identified in sarcopterygians and actinopterygians fell into four paralog groups. Matching sequences for all four paralog groups were found in chondrichthyans, indicating that four *Dacts* genes were present in the ancestral gnathostome genome. The first group encompassed known Dact1 sequences and their newly identified relatives. Dact1-type proteins consisted of 800-850aa with 56.0% overall sequence identity; they were encoded by three small and a 4^th^, large exon. Sequences of this type were found in all gnathostomes with the exception of pufferfish. In all species, only a single *Dact1* gene was present (Figure 1). A second set of sequences was 750-850aa long with overall 40.6% sequence identity and encompassed known and novel Dact2 proteins. The *Dact2* genes showed the same intron-exon structure as *Dact1* genes, however the third exon was almost twice as long as the 3^rd^ exon in *Dact1*. Dact2-type sequences were found in all gnathostomes with the exception of amphibians. Similar to Dact1, only a single *Dact2-*type gene was found in a given species. The third set of sequence encompassed both previously and newly identified Dact3 proteins which were present in all gnathostomes with the exception of birds. In teleosts, two distinct sets of *dact3* genes were found, designated *dact3a* and *dact3b*; a possible exception is the stickleback where due to gaps in the genomic sequence and absence of *dact3b* ESTs, the presence of this gene could not be ascertained. The Dact3 proteins showed significant length variations, ranging from 420 (*Xenopus*), 540–660 (teleosts), 610–630 (mammals) to 820aa (*Latimeria*). Given that the Dact family was thought to consist of three members only [3,4,24-28], we were surprised to find a fourth, distinct set of sequences. Dact4 proteins encompassed some 700 (Anole lizard), 830 (*Latimeria*), 990 (zebrafish) or up to 1070-1120aa (acanthopterygian teleosts). Like most Dacts, Dact4 proteins were encoded by genes containing four exons. The exception was a second gar and zebrafish dact4 protein which stems from an intronless gene that possibly was retrotranscribed and hence was named *dact4r*. Remarkably, *Dact4*genes were present in chondrychthians, in actinopterygians and in the following sarcopterygians: *Latimeria*, anapsid and diapsid reptiles. This suggests that the *Dact4* gene belongs to the original gnathostome Dact repertoire and persisted well beyond the actinopterygian-sarcopterygian split, the coelacanth-tetrapod split, the amphibian-amniote split and the segregation of the amniote lineages, but was lost independently in the avian, mammalian and amphibian lineages. Since both the gar and the zebrafish have *dact4r* genes, this suggests that the gene occurred before the teleost-specific, third genome duplication (3R) [21,22], but in most teleosts it was eliminated together with the duplicate of the genuine *Dact4* gene.

### Identification of cyclostome *Dact* genes

Given that we found *Dact* genes well-represented in all gnathostome lineages, we wondered whether cyclostomes that split from gnathostomes some 536 million years ago [29] might also carry these genes. We therefore searched the Ensembl and NCBI databases for *dact* family members in the two cyclostome genomes available (*Petromyzon marinus* and *Lethenteron japonicum*). As queries, we used full-length, exon-specific or motif-specific sequences from all four gnathostome Dact proteins. The search revealed several contigs with dact-like sequences in the *Lethenteron* genome and also in the PetMar1 version of the sea lamprey genome. When the current version of the sea lamprey genome (PetMar2) was released, however, all except the sequences previously located on contig 36439, now GL476511, had been removed. Yet several of the original *Petromyzon* contigs encoded conserved Dact motifs in the correct order, they had highly similar counterparts in the *Lethenteron* genome, and some sequences were also represented in ESTs. We therefore considered these sequences as trustworthy. The results are included in Figure 1 and Additional file 3.

The analysis of the lamprey genome and EST sequences indicated the existence of at least four dact-related genes in cyclostomes (*dactA-D*). For two of these genes, sequences corresponding to all four *Dact* gene exons were located on single contigs in Lethenteron (*dactA* on KE993709, *dactB* on KE993739). Partial matches for both genes were found in the *Petromyzon* genome (PetMar1 c36439/PetMar2 GL476511 for *dactA* and PetMar1 sc37220/c20195 for *dactB*). For *dactC*, only exons 2–4 were identified on contig KE9993726. Sequences with high similarity to exon4 of *dactC* were found on two more *Lethenteron* contigs (KE999188 and KE995210), but not in the *Petromyzon* genome. Contig KE994909 of *Lethenteron* contained exon4 of the *dactD* gene, also represented in PetMar1 c54804. In addition, identical, likely exon1 sequences were found on contigs APJL01152884 and APJL01160608. Since these sequences were not contiguous with the *dactC* or *dactD* sequences, they could not be unambiguously assigned to either gene.

While the four cyclostome *dact* genes displayed similarity with the other vertebrate *Dacts*, they could not be clearly allocated to any of the gnathostome *Dact* paralog groups.

### Identification of invertebrate *dact* genes

To trace the so far elusive origin of *dact* genes, we next searched the Ensembl and NCBI genome and EST collections for *Oikopleura dioica*, *Ciona intestinalis*, *Ciona savignyi* (non-vertebrate chordates, subphylum tunicates), *Branchiostoma floridae* (non-vertebrate chordates, subphylum cephalochordates), *Saccoglossus kowalevskii* (hemichordates) and *Strongylocentrotus purpuratus* (echinoderms). These are all deuterostome animals. In addition, we searched the sequences available for the following protostomes: *Aplysia californica*, a mollusc representing lophotrochozoans; *Drosophila melanogaster*, *Tribolium castaneum*, *Bombyx mori* (insects, ecdysozoans) and *C. elegans*, *C. briggsae* and *Loa loa* (nematodes, ecdysozoans). Finally, we interrogated the NCBI protist and fungi genomes. The searches were performed as before, using full length or exon-specific Dact protein sequences or protein motifs as queries.

Our results revealed that the only invertebrate harboring dact sequences was the cephalochordate *Branchiostoma floridae*, the Florida lancelet (Figure 1; Additional file 3). Here, the blast hits matched with exons 8–10 of a predicted 10-exon cDNA on a single scaffold (s65). Exons 1–7 were confirmed by ESTs, encoding however the lancelet homologue of the *RPA2* gene. Exons 8–10 were confirmed by two further sets of ESTs. The first set encompassed exon8, 9 and start to mid-exon10. The second set carried middle and end of exon10. Yet there are no ESTs to suggest that exons 1–10 are linked in a transcript. Moreover, as will be shown below, exons 8–10 carry the complete sequence for a *dact* gene. We therefore renamed the exons that belong to *Branchiostoma dact* exons1,2,3. Exon1 encoded 73aa with loose homology to exon1 derived sequences in vertebrate *Dacts*. Exon2 accounted for 58aa that aligned well with exon2-derived sequences of gnathostome Dact1-3, including a 5x leucine zipper. Different to vertebrates, however, the *Branchiostoma* exon2-3 boundary encoded an extended serine-rich stretch. Exon3 encoded in total 872aa that encompassed a number of the conserved sequence motifs which in vertebrates are encoded by the 3’ end of exon2, and by exons 3 and 4. Taken together, we traced the origin of *dacts* back to chordates, where many motifs and functional domains were established already.

### Phylogenetic analysis of Dact protein sequences

**Figure 2:**
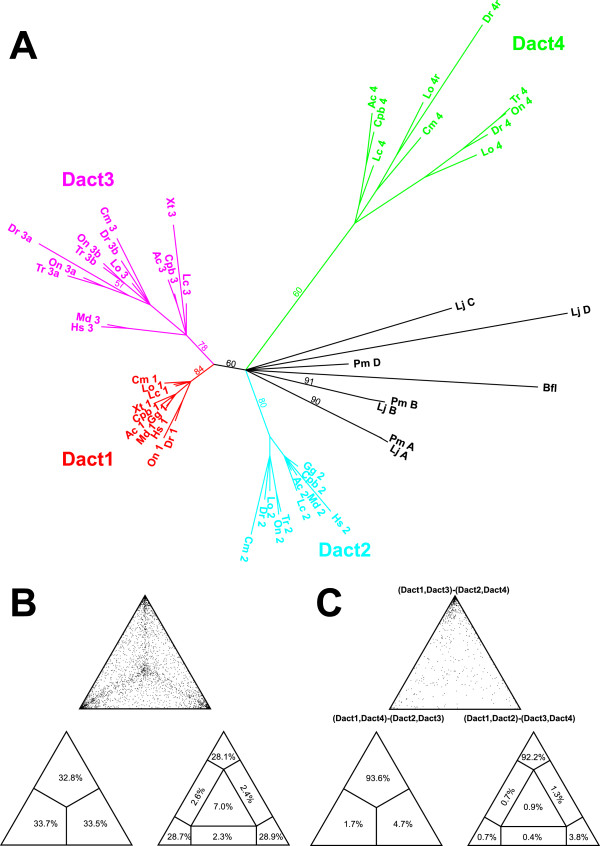
**Phylogenetic analysis of Dact proteins.** Reconstruction of the phylogenetic tree of Dact proteins and likelihood mapping by quartet puzzling using Tree-Puzzle. **(A)** Unrooted phylogenetic tree of Dact protein sequences from humans (Hs), opossum (Md), chicken (Gg), Anole lizard (Ac), the Western painted turtle (Cpb), *Xenopus tropicalis* (Xt), Latimeria (Lc), the spotted gar (Lo), zebrafish (Dr), Fugu (Tr), Tilapia (On), elephant shark (Cm), sea lamprey (Pm), Japanese lamprey (Lj), and *Branchiostoma floridae* (Bfl). The tree was created using the JTT model with accurate parameter estimation and using 100,000 puzzling steps. Likelihood values are indicated for branch points separating major groups. Sequences are annotated using the abbreviation for the species, followed by the Dact ortholog number. Note that the gnathostome Dact1 (red branches) and Dact3 sequences (pink branches) formed a metagroup. Dact2 (turquoise branches) and Dact4 sequences (green branches) each formed distinct groups. They emerge from a star-like node together with the four cyclostomes dact proteins and the *Branchiostoma* dact sequence, indicating the ambiguity of the tree topology for this part of the tree. **(B)** Likelihood mapping of the Dact protein sequences used for the phylogenetic tree reconstruction, based on 10,000 random quartets. 85.7% of quartets were fully resolved, indicating overall tree-like character. **(C)** Likelihood mapping of the Dact1, Dact2, Dact3 and Dact4 clusters, based on 10,000 random quartets. 92.2% of quartets support the Dact1/3 versus Dact2/4 subdivision.

The initial sequence analysis of the known and the newly identified Dact sequences suggested that until recently, both sarcopterygian and actinopterygian vertebrates had four distinct *Dact* genes that were generated during the second genome duplication in vertebrate evolution (2R) [20]. To further corroborate this finding and to determine which of the *Dact* genes are more related and hence, originated from a common ancestor, we carried out a phylogenetic analysis of Dact proteins, using maximum likelihood and Bayesian methods (PhyML, IQTree, MrBayes and TreePuzzle). To ensure that the major chordate taxa are represented, we focused on sequences from humans, opossum, chicken, Anole lizard, the Western painted turtle, *Xenopus tropicalis*, coelacanth, spotted gar, zebrafish, Fugu, *Tilapia* and *Branchiostoma* that were full length or near full length; in addition we included the partial sequences from the elephant shark, and the complete and partial sequences from the two cyclostomes, *dactA-D* from *Petromyzon* and *Lethenteron*. We used an unbiased approach, i.e. an unrooted tree (Figure 2A; trees rooted from the *Branchiostoma* sequence are shown as Additional file 4). Likelihood mapping shows that 85.7% of quartets were fully resolved (Figure 2B), indicating the sequences were suitable for phylogenetic reconstruction.

In the tree, the gnathostome sequences were placed into four distinct groups (Figure 2; Dact1: red, Dact2; turquoise, Dact3; pink, Dact4; green). Within the Dact3 group, the Dact3, 3a and 3b sequences formed the expected subgroups. Likewise, the gar and zebrafish dact4r sequences formed a subgroup within the Dact4 group. Thus the phylogenetic tree analysis supports our Dact1-4 group allocations. Within the individual Dact groups, sarcopterygian and actinopterygian Dact sequences formed subgroups, particularly evident in the rooted trees (Additional file 4). The position of the elephant shark sequences was less clear, possibly because these sequences are incomplete. Interestingly, in the unrooted tree and the rooted trees, the gnathostome Dact1 and Dact3 sequences formed a meta-group. The gnathostome Dact2 and Dact4 sequences formed a second metagroup, evident in the maximum likelihood and Bayesian trees (Additional file 4B-C). The division into the Dact1/3 and Dact2/4 groups was highly significant in the likelihood mapping analysis (92.2%, Figure 2C) and well supported in the PhyML tree for gnathostome sequences (bootstrap value of 100; Additional file 5). This suggests that of the two *Dact* genes created in 1R, one gave rise to *Dact1* and *3*, the other to *Dact2* and *4* genes.

In the maximum likelihood and Bayesian phylogenetic trees for all vertebrate sequences (Additional file 4B-C), the cyclostome sequences were grouped together, separated from the gnathostome Dacts. The quartet puzzling tree (Figure 2A), however, shows a star-like topology for this node, and consequently the evolutionary relationship of cyclostome and gnathostome genes cannot be determined with certainty.

### Organization and relationship of gnathostome *Dact* gene loci

**Figure 3:**
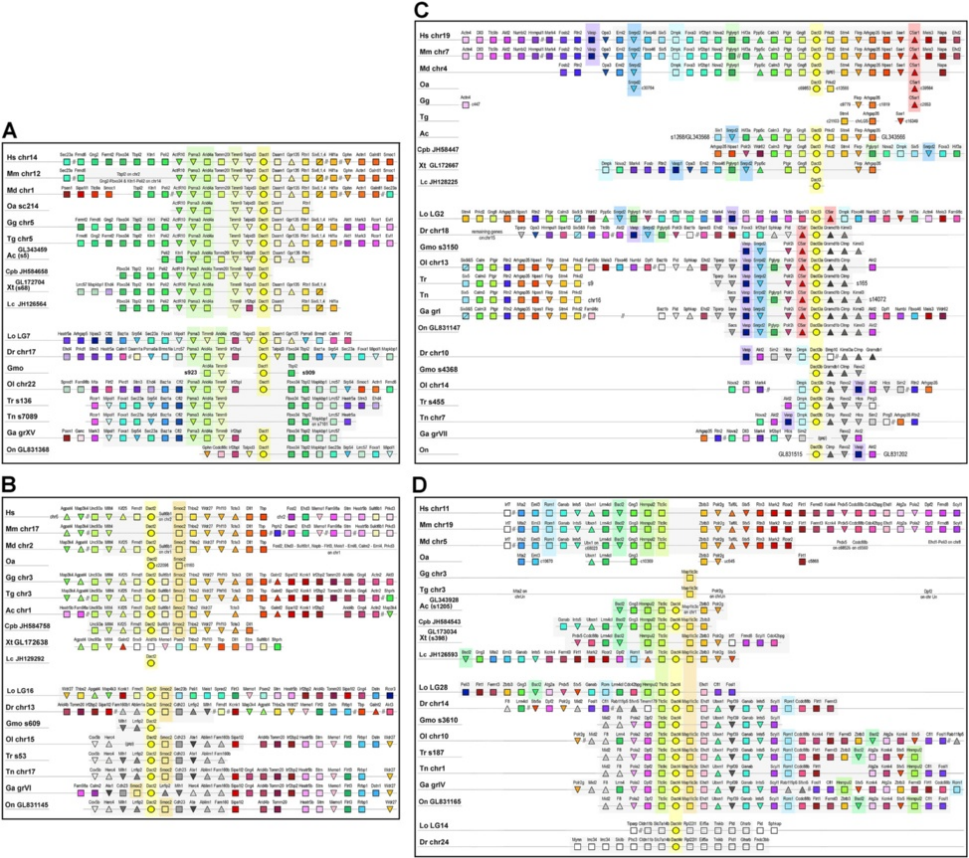
**Organization of gnathostome *Dact *genomic loci.** Genomic environment of the Dact1 **(A)**, Dact2 **(B)**, Dact3 **(C)** and Dact4 **(D)** genes. Shape code: circle - *Dact* gene; square - gene with paralogs also associated with *Dact* genes; triangle, tip down - unique gene without any paralogs; triangle, tip up - gene with paralogs not associated with *Dact* loci. Shape infill: yellow - *Dact* genes; other colours - genes associated with tetrapod and teleost *Dact* loci. A rainbow color scheme was applied to the human locus in the case of *Dact1/3* and *4*; in the case of *Dact2*, the mammalian loci are fragmented, hence the rainbow color scheme was applied to the better preserved chicken locus. Orthologous genes are displayed in the same color. Gray infill - genes associated with teleost *dact* genes only; striped pattern - genes associated with *Dact* loci in teleosts, non-mammalian tetrapods and the opossum, but dispersed in placental mammals. The color of the stripes corresponds to that of the neighboring filled-in shape in teleosts. A diagonal bar in the boxes representing *Six* genes indicates the presence of several *Six* paralogs at this site. Underlying shading: yellow shading - *Dact* genes; other colours - core genes associated with a particular *Dact* gene; grey shading - genes within 1 Mb distance from *Dact* genes. Double slash - genes or gene groups separated by more than 3 Mb. Species names (abbreviations: see Additional file 1) and genomic localization of genes are indicated on the left side of the figures if the loci are continuous. If genes are distributed over several scaffolds or chromosomes, the names of these sites are shown next to the corresponding genomic fragment. Note that the same genes, albeit not always in the same order, are associated with a particular *Dact* ortholog; also note the similarity of the teleost *dact3a* and *3b* loci, further indicating their common origin from an ancestral *dact3* gene during 3R. This supports our assignment of gnathostome Dact sequences to four paralog groups. A number of genes are only found in conjunction with teleost *dact* genes, suggesting that they have invaded the locus after the two round of genome duplication shared by sarcopterygians and actinopterygians, but before the third, teleost-specific genome duplication.

Our study revealed novel gnathostome Dact sequences that were allocated to four paralog groups, based on the combination of aa sequence features and the phylogenetic analysis. To further corroborate this allocation, we analyzed the organization of vertebrate *Dact* genomic loci, reasoning that *Dact* orthologs would reside in syntenic genomic regions. For our analysis, we focused on representative sarcopterygian and actinopterygian species with reasonably well characterized genomes. We first determined the localization of a given *Dact* gene, performing a Blast search on the Ensembl database. We then established the order of neighboring genes in a 1–2 Mb radius (Figure 3), exploiting the Ensembl gene annotations or performing Blast-searches for these genes. During this process, we noticed that, following inversions and other forms of recombination events, genes associated with a particular *Dact* gene in sarcopterigians often had been placed at a distance in actinopterygians, and vice versa. We therefore also established the wider environment of *Dact* genes (Additional file 6).

### *Dact1* loci

Genes assigned to the *Dact1* group were invariably linked with *Timm9*, *Arid4a*, *Psma3* (exception: the gap-riddled contig carrying *TilapiaDact1*; Figure 3A). In the gar, *talpid3* and *irf2bpl* were found between *dact1* and *timm9*; the two genes were also next to *dact1* in Tilapia or on either side of *dact1* in the zebrafish. In all other organisms, either *Talpid3* (tetrapods) or *Irf2bpl* (*Latimeria*, most teleosts) was located between*Timm9* and *Dact1*. In sarcopterygians as well as in the gar, on the side facing away from the *Psma3*-*Talipd3/Irf2bpl* group, *Dact1* was associated with *Daam1* and *Gpr135*. In teleosts, this position was held by *fbxo34* and *tbpl2*, which in sarcopterygians were part of a gene group linked to *Psma3*. Outside the immediate 1 Mb radius around *Dact1*, numerous additional genes were found both in the wider environment of sarcopterygian as well as actinopterygian *Dact1* (Additional file 6). Thus, although there is some variation in the arrangement of *Dact1* loci, the same genes were associated with *Dact1* in sarcopterygians and actinopterygians. Of these genes, *Psma3*, *Timm9* and *Talpid3* are single genes without any paralogs. Hence, they serve as unique identifiers of the *Dact1* locus, and support our assignment of genes to the *Dact1* group.

### *Dact2* loci

As amphibians lack a *Dact2* gene and *Latimeria dact2* was on a too short a contig, information on sarcopterygian *Dact2* loci was restricted to amniotes. However, in amniotes as well as in the gar, genes allocated to the *Dact2* group were associated with *Frmd1* on one side and *Smoc2* on the other; in teleosts, *smoc2* was also always present (Figure 3B). *Thbs2* and *Wdr27*, linked to *Smoc2* in amniotes, were within 1 Mb distance of *dact2* in the gar and only slightly more distant in teleosts (Additional file 6). Similarly, the *Map4k3-Agpat4* group was found in the wider environment of all *Dact2* genes, and the *Sipa1l2-Irf2pb2-Gng4* group was in the wider environment of bird, reptile and actinopterygian *Dact2* (this region is more dispersed in mammals). As for *Dact1*, numerous additional genes populated the *Dact2* environment both in amniotes as well as in actinopterygians. Moreover, *Wdr27*, and in amniotes *Phf10* and *Mllt4*, are unique and serve as locus identifiers, suggesting that we allocated *Dact2* orthologs correctly. In teleosts, a number of genes are linked with *dact2* that are not found in the *dact2* environment of the gar, suggesting that they invaded the locus after the split from the holost lineage (Figure 3B, Additional file 6; grey symbols). Remarkably, traces of *Dact2* locus can still be found in *Xenopus*, since a number of *Dact2* associated genes are well preserved on contig GL172638.

### *Dact3* loci

For the genes assigned to the *Dact3* group, only limited information was available for platypus and *Latimeria* (Figure 3C, Additional file 6). In all other animals, *Dact3* genes were accompanied by *Vasp*, *Snrpd2*, *Dmpk*, *Pglyrp* and *C5ar* (tetrapods, gar), *Vasp*, *Snrpd2*, *Pglyrp* and *C5ar* (most teleost *dact3a* genes), or *Vasp*, *Dmpk* (teleost *dact3b* genes). The *Prkd2-Fkrp-Arhgap35* group that is closely linked to amniote *Dact3* is found in the wider environment of gar *dact3* and teleost *dact3a*, while a duplicated copy of the Argap35 gene is found in the environment of teleost *dact3b*. Similarly, genes like *Rtn2*, *Akt2,Polr2i*, *Opa3, Ppp5corSae1*are found in the wider environment of all *Dact3* genes, with *Polr2i*, *Opa3*, *Snrpd2*, *Fkrp* and *Sae1* being unique, and *Ppp5c* and *C5ar1* having no paralogs linked to other *Dact* genes. Thus, even though the precise order of genes differs between gnathostome groups and a number of signature genes have disappeared from the teleost *dact3b* locus, all loci are recognizable as related, supporting our assignment of genes to the *Dact3* group. A set of genes was only found at teleost *dact3* loci, yet these were present both at the *dact3a* and *3b* locus. This indicates that the teleost *dact3a* and *dact3b* genes arose from the teleost-specific 3R [22]. In birds, however, almost all of the *Dact3* associated genes were absent, suggesting that the entire locus has been lost.

### *Dact4* loci

As shown above, *Dact4*-type genes were only found in anapsid and diapsid reptiles, in *Latimeria* and in actinopterygians, and the sequences of the sarcopterygian and actinopterygian proteins were rather divergent. Yet *Dact4* genes were invariably linked with *Ttc9*, and in most cases, also with *Map1lc3c* (Figure 3D). In reptiles and the gar, *Ttc9* was adjoined by *Hnrnpul2*, which was located in the *Dact4* environment in teleosts. In the sarcopterygians, *Map1lc3c* was linked with *Zbtb3* and *Polr2g*, which populated the environment of actinopterygian *dact4* genes (Figure 3D, Additional file 6). *Bscl2* was located within the 1 Mb environment of all *Dact4* genes, and in the gar and teleosts (contigs too short for the reptiles), also *Rom1* was close by. In acanthopterygian teleosts, the *dact4* environment showed a stereotype arrangement, and most of the genes found here were also found in the environment of the zebrafish, gar, coelacanth and reptile *Dact4*. Of the genes associated with *Dact4* loci, *Bscl2*, *Ints5*, *Polr2g* and *Stx5* are unique and therefore, identify this site. Thus, even though the order of genes at *Dact4* loci was not always preserved, the loci, and by extension the genes and proteins were closely related. Searching for *Dact4* associated genes in vertebrates that have lost *Dact4*, we noticed that the locus was very well-conserved in mammals and in amphibians, suggesting that their *Dact4* genes disappeared as a result of only a small deletion and possibly recently. In contrast, in birds only a few dispersed genes formerly associated with *Dact4* were present, suggesting a major chromosome rearrangement that resulted in the loss of the entire locus. The intronless *dact4r* gene found in the gar and zebrafish, however, was not accompanied by any genes linked to the original *dact4*. Yet, the *dact4r* loci closely resembled each other. This suggests that the *dact4r* gene was present in the ancestor of holosts and teleosts before the teleost 3R, but was shed from most teleost genomes thereafter.

### Phylogenetic analysis of *Dact*-associated sequences

Our synteny analysis revealed a number of *Dact*-associated genes specific for a particular *Dact* locus. However, we also found a number of genes with paralogs at several *Dact* loci, suggesting that they were part of the *Dact* locus before the gnathostome 2R. We therefore expected that, if our phylogeny analysis of the Dacts were correct, the Dact associated sequences would show the same phylogenetic relationships. To test this, we scanned the environment of *Dact* genes for genes that have four paralogs in all vertebrates, each associated with a particular *Dact* locus, making allowances for teleost genes that, after 3R were kept at the locus that since has shed the duplicated *Dact* gene. These criteria applied to *Ehd1-4; Eml1-4; Fos, Fosb, Fosl1, Fosl2; Mark1-4; Rtn1-4 and Sipa1, Sipa1l1, 1 l2, 1 l3* (genomic location: see Figure 3, Additional files 6). Interestingly, a *Sipa1* homologue was found associated with *dactA*, and an *Eml* homologue close to *dactB* in the *Lethenteron* genome (not shown). We next extracted the protein sequences encoded by these genes, and wherever possible, the corresponding lamprey, *Branchiostoma*, tunicate or *Drosophila* sequences, and, using the *Drosophila* sequences as outgroups, we constructed phylogenetic trees (Additional file 7). Notably, the trees obtained for the *Dact*-associated genes always grouped the *Dact1/3* and *Dact2/4* associated genes; the other possible permutations (*Dact1*/*2*; *Dact1*/*4*; *Dact3*/*2*; *Dact3*/*4*) were never observed. This supports the idea that during the vertebrate 2R *Dact1-Dact3* arose from one, *Dact2-Dact4* from the other *dact* precursor.

### Analysis of structural motifs in the Dact protein groups

**Figure 4:**
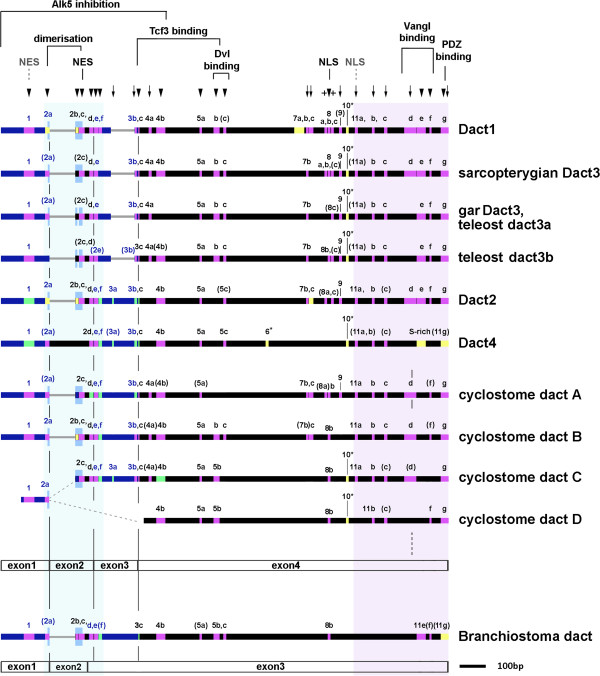
**Conserved Dact protein motifs.** Graphical display of the gapped Dact protein alignments (thick horizontal lines); large sequence stretches missing in a particular Dact are shown by thin grey lines. Purple: shared protein motifs;red: shared Dact1/3 motif variations; green: shared Dact2/4 motif variations in green; yellow: motifs typical for a particular Dact ortholog; turquoise: in motif 11 g, the cyclostome dactA and dactB proteins share amino acids that are specific for either Dact2 or Dact1/3. The leucine zipper is marked by mid-blue, higher boxes. The lengths of motifs are according to scale. The light blue and lilac background shading indicates the most conserved areas of Dact proteins. Motifs are numbered according to their position in the Dact alignment; linked motifs are marked by letters, partial versions of a motif are in brackets. Known roles of motifs or sequence stretches are indicated at the top, predicted roles are marked in grey and with dotted lines. Exon boundaries are indicated below a set of sequences; note that the exon2-3-4 boundaries are different in vertebrates and *Branchiostoma*, and that the cyclostome dactA features a fourth intron within motif 11d. In the cyclostomes, a genomic fragment carrying a recognizable dact exon1 sequence was not linked to the fragments carrying the dactC or dactD sequences, hence the exon1 carrying fragment may belong to either (dotted grey lines). Importantly, some motifs such as the leucine zipper are present already in the lancelet and hence, constitute the original repertoire of dacts (marked by arrowheads). Other motifs arose in the vertebrate (arrows) or, subsequently, in the gnathostome lineage (crosses). Gnathostome Dact orthologs have a unique composition of motifs. However, motifs are the most similar in Dact1/3 and Dact2/4, respectively. Cyclostome dact proteins resemble a mix of Dact1/3-typical sequences, Dact2/4 sequences, and unique sequences.

Dacts have been attributed a range of functions in intracellular signaling pathways, all relying on their interaction with other proteins. The ability to interact with partners resides in distinct structural motifs. The identification of a whole family of distinct Dact paralogs raises the possibility that different Dacts specialize in specific functions, and that this may be reflected in their repertoire of motifs. We hence investigated the exon-intron structure of *Dact* genes, and we investigated the presence and distribution of known protein motifs, searched for the presence of further conserved aa stretches and used the PSort and NetNes 1.1 programs to predict functionally relevant motifs. For the ease of comparison, motifs were numbered consecutively; where protein motifs were composed of several linked elements, these were labeled with letters in alphabetical order. The identity matrix for the most conserved regions is included in Additional file 8. Presence and linear distribution of the motifs is shown in Figure 4; the sequences of short motifs are summarized in Additional file 9, motifs and longer conserved stretches are indicated in the full alignments of Dact orthologs (Additional file 10) as well as in the gnathostome Dact sequence logos (Additional file 11). Our approach revealed novel sequence motifs typical for all Dact proteins. Significantly, we also identified motifs and sequence variations that distinguish Dact orthologs and that, even in individual species with six *Dact* genes, assigned them to the four paralog groups.

### Dact1-type sequences

Conserved stretches of aa in the Dact-1 type proteins included a putative nuclear export signal encoded by the centre of exon 1 (motif 1), a series of linked elements spanning the 3’ end of exon1, exon2 and the 5’ end of exon3 (motifs 2a-f, 90.4% identity) which included a 6x leucine zipper required for homo-and heterodimerization [9] and a nuclear export signal [12], and in comparison to Dact2 a reduced set of elements encoded by the exon3/4 border (motifs3b,c). Exon 4 continued with sequence motifs 4a,b, 5a-c; functionally, the region encompassing motifs 3c-5b has been implicated in Tcf3 binding; the region encompassing motifs 5b,c was shown to participate in Dvl binding [8,13]. Following a variable portion, further conserved aa stretches (motifs 7a, b, c, 8a-c, 9, 10) including a nuclear localization signal [12]were recognizable, with motif 7a and the specific sequence of the 10^th^ motif only occurring in this protein group. The last 200aa with motif elements 11a-g were again highly conserved (81.8% identity) and encompassed a further putative nuclear localization signal, the known Vangl binding domain and the C-terminal PDZ binding domain [8].

### Dact2-type sequences

In the Dact2 proteins, exon 1 encoded a distinct version of motif 1, which was followed by the exon1-3 spanning domain that had 85.1% identity, contained motifs 2a-f, a 6x leucine zipper and the nuclear export signal. Yet the specific sequence of motif 2f was distinct from the corresponding sequence in Dact1 proteins. The 3’ end of exon 3 encoded two sets of sequences (designated motifs 3a,b) that both resembled Dact1 motif 3b, indicating that they may have arisen from an internal duplication. Exon 4 contributed to a specific version of motif 3c, followed by motifs 4b, 5a, motif 5c, motifs 7b and 7c, incomplete motifs 8a,c and motif 9. The C-terminus displayed 61.2% identity and encompassed motifs 11a,b, partial motif 11c, motif 11d, a distinct version of motif 11e, motif 11f, and a terminal motif 11 g that was reminiscent of the lamprey dactA-C sequences. Compared to Dact1, motifs 4a, 5b, 7a, the nuclear localization signal motif 8b and motif 10 were missing.

### Dact3-type sequences

Not surprisingly, given the differences in sequence length, Dact3 proteins had only 26.3% overall sequence identity. However, these proteins shared a number of features that distinguished them from the other Dact-types. Dact3-type proteins harbored motif 1, partial motifs 2c-e and 3b, motif 3c, 4a, 5a-c, 7b, incomplete motif 8c, motif 9, motif 10, partial motif 11a, and well recognizable motifs 11b,c,e,f,g. Motifs 2a, 4b, 8a and 11d were present in some but not all Dact3 proteins; motifs 2b, 2f, 3b, 7a, 7c were always absent. Interestingly, motifs 1, 4a, 5b, 7b, 11e and the PDZ binding domain containing motif 11 g resembled the corresponding Dact1 motifs more than those of Dact2; overall Dact3 motif 11 had 43.6% identity with that of Dact1 and 31.8% identity with motif 11 of Dact2. Most remarkable however was a strong reduction of the leucine zipper. Owing to sequence variability at the 3’ terminus of exon 1 and start of exon 2, this region did not regularly provide a suitable leucine to contribute to the leucine zipper. Exon 2 encoded for several leucines, but in *Latimeria*, the gar and the teleost dact3a proteins, a loss of 3aa interrupted the regular array of leucines, in most animals leading to a 3x plus 2x leucine zipper arrangement (Additional file 12). Since these animals represent both the sarcopterygian and the actinopterygian lineage, we concluded that the interruption of the leucine zipper had occurred before the sarcopterygian-actinopterygian split. In tetrapods, further 4aa were lost, such that 2–4 correctly placed leucines restored a 3x-5x leucine zipper. On the other hand, in teleost dact3b sequences, the leucine zipper was further reduced with *Tetraodon* dact3b lacking it altogether.

### Dact4-type sequences

The overall conservation of the Dact4 protein sequences was low, but several recognsizable motifs showed much higher sequence similarity. Dact4 proteins harboured sequence motifs 1, incomplete motif 2a, motifs 2d,e,f, partial motif 3a, motifs 3b, 3c, 4b, 5a, 5c, a Dact4-specific motif 6, a Dact4-specific motif 10 and partial motifs 11a-c. In teleosts, motifs 5c and 6 were separated by a repetitive stretch consisting of repetitive asparagines and leucines; motifs 6 and 10 were separated by a stretch enriched in serines, histidines and prolines. The proteins concluded with a serine-rich domain that was ill-conserved between sarcopterygians and actinopterygians but may represent a degenerate version of motif 11e, followed by a number of alkaline and neutral aa resembling Dact1-3 motif 11 g. Thus, while these proteins evolved some new motifs, a number of motifs present in other Dacts were lost. Importantly, these newly identified Dact proteins lacked the PDZ binding domain, suggesting that they may not be able to interact with Dvl. Similarly, exons 1–2 did not encode a leucine zipper, indicating that these proteins may be unable to homo- or heterodimerize.

### The cyclostome dact proteins

The cyclostome dact proteins share many of the conserved motifs identified in the gnathostome Dacts. Motifs 1-5c, 7b-c, 8b, 9, 11a-d, 11f and 11 g were well recognizable in at least one of the cyclostome proteins, and often in all of them. A leucine zipper was recognizable in all available sequence. The dactA protein had a small 2x leucine zipper encoded by exon 2, while dactB showed a bipartite, 2x plus 3x, leucine zipper. No information was available for exon 1 of *dactC*, but exon 2 encodes a 2x leucine zipper. The orphan exon 1 sequence had a 3x leucine zipper. Interestingly, in the *dactA* gene of both *Petromyzon* and *Lethenteron*, the 11d motif was split by an additional intron, so that the *dactA* gene is comprised of five exons. Some of the motifs shared aa characteristic either for the Dact1/3 proteins or for the Dact2/4 proteins (Figure 4, Additional file 9), but none of the cyclostome dact protein matched with either of these gnathostome metagroups.

### The *Branchiostoma* dact protein

The *Branchiostoma* dact protein was the most divergent of the proteins we analyzed. Sequences included a recognizable motif1 and a partial motif 2a, and contributed one leucine to a leucine zipper. Exon2 accounted for 58aa that aligned well with exon2-derived sequences of gnathostome Dact1-3, contributing to motifs 2b,c, and to further leucines for an in total 5x leucine zipper. Different to vertebrates, however, the *Branchiostoma* exon2-3 boundary encoded an extended serine-rich stretch. Exon3 encoded in total 872aa that encompassed sequences which in vertebrates are encoded by the 3’ end of exon2, and by exons 3 and 4, including motifs 2d,e, an incomplete motif 2f, motifs 3c, 4b, partial 5a, motifs 5b, c, the nuclear localization signal associated with motif 8b, motif 11e that was enriched in acidic aa and serines, and partial motifs 11f,g. Notably, motifs 5b,c were separated by an extended stretch of 130 aa, and the PDZ binding domain was missing. Of the motifs present in *Branchiostoma* dact, motifs 1 and 5b were more similar to motifs in Dact1/3 than to Dact2/4, while motifs 2f and 3c more strongly resembled motifs present in Dact2/4. Taken together, we traced the origin of *dacts* back to chordates, where many motifs and functional domains were established already.

### Comparative expression analysis

**Figure 5:**
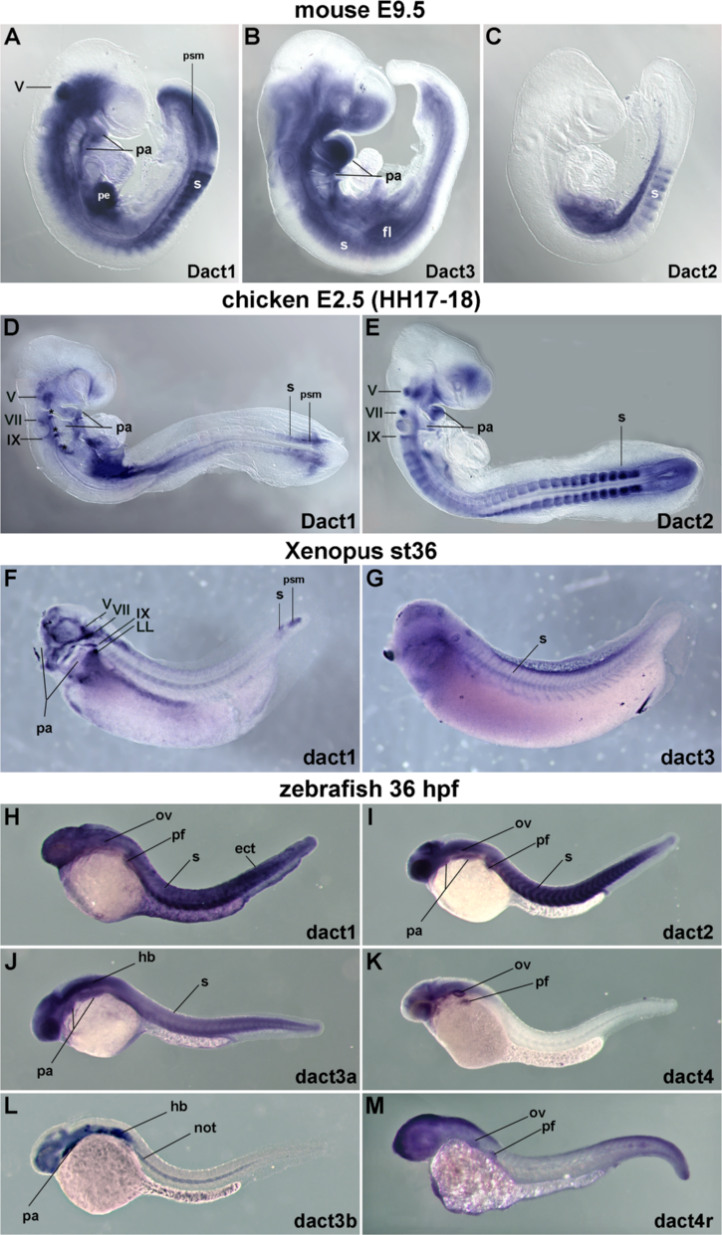
**Comparison of *Dact *gene expression in mouse, chicken *Xenopus *and zebrafish embryos. (A-C)** Lateral views of E9.5 mouse embryos, anterior to the top left; **(D,E)** of E2.5 (HH17-18) chicken embryos, anterior to the top left; **(F,G)** of st36 Xenopus laevis embryos, anterior to the left, and **(H-M)** of 36 hours post fertilization (36hpf) zebrafish embryos, also anterior to the left. The embryos are at the phylotypic stage of vertebrate development; they were assayed for mRNA expression of *Dact* genes as indicated in the panel. Note that for members of both the *Dact1/3* paralog group, as well as for the *Dact2/4* paralog group, prominent expression was found in the paraxial mesoderm, craniofacial mesenchyme, pharyngeal arches and cranial ganglia as well as the developing paired limbs/ fins (for the chicken; this expression emerges at E3; [31]), suggesting that these are original sites of *Dact* function. At a number of sites, expression of *Dact* paralogs overlaps, suggesting that here Wnt and Tgfβ signal transduction is controlled by combinatorial Dact activity. Abbreviations: drg; dorsal root ganglion; ect, surface ectoderm; fl, fore limb bud; hb, hindbrain; hl, hind limb bud; LL, caudal lateral line placode; not; notochord; ov, otic vesicle; pa, pharyngeal arches; pe, proepicardium; psm, presomitic mesoderm; pf, pectoral fin; s, somites; V, trigeminal ganglion; VII, facial ganglion; IX, glossopharyngeal ganglion; the asterisk marks the epibranchial placodes.

Our analysis showed that initially, jawed vertebrates were equipped with four *Dact* genes, of which mammals lost *Dact4*, puffer fish lost *dact1*, amphibians lost *dact2* and *dact4* and birds lost *Dact3* and *Dact4*. On the other hand, after the teleost-specific 3R, these animals kept two *dact3* genes and hence, gained a *dact* gene. Zebrafish and gar, by retaining the retrotranscribed *dact4r* gene, gained a further *dact* gene. All these genes may still show aspects of their original expression patterns and cooperate in a given tissue. Alternatively, their expression domains may have been redistributed, with each gene acquiring unique sites of action. To investigate this, we comparatively analyzed *Dact* gene expression in animals with the most divergent complements of Dact genes: mouse (three *Dact* genes), chicken (two *Dact* genes), *Xenopus* (two *dact* genes, but both belong to the *dact* 1/3 group) versus zebrafish (six *dact* genes). We focused primarily on pharyngula-early somite stage embryos since at this stage, vertebrate embryos are the most similar (phylotypic stage; [30] and references therein). At this stage (9.5 dpc), mouse *Dact1* was expressed widely, with highest expression levels in the presomitic mesoderm and young somites, the proepicardium, the craniofacial mesenchyme and pharyngeal arches and the trigeminal ganglion. *Dact3* was also expressed widely, with strong signals in somites, the pharyngeal arches and the forelimb bud. *Dact2* showed prominent expression in young somites and the developing intestine (Figure 5A-C; [27]); in more strongly stained specimen, all somites as well as the trigeminal, facial and glossopharyngeal ganglia were labeled (not shown). Chicken *Dact1* was expressed in the presomitic mesoderm and young somites, the craniofacial mesenchyme, the splanchnopleural lateral mesoderm, several cranial ganglia and the epibranchial placodes (Figure 5D; [27]); expression in the mature somites, in the limb mesenchyme and the dorsal root ganglia emerged slightly later at E3 ([27,31]. Chicken *Dact2* is known for its early expression in the cranial neural crest [27]. At E2.5, the gene was expressed in the somites, the craniofacial mesenchyme, and several cranial ganglia (Figure 5E; [27]). Later at E3, the gene was also expressed in the mesenchyme surrounding the dorsal root ganglia, the limb buds, the lung bud and the eye [27,31]. *Xenopus dact1* expression was initially found in the dorsal blastopore lip, the neural plate, the emerging neural crest cells and the emerging paraxial mesoderm ([3] and not shown). At stage 36, the gene was expressed in the presomitic mesoderm and young somites, the lateral mesoderm and in several cranial ganglia and the posterior lateral line placode (Figure 5F); in more strongly stained specimen, staining was seen in all somites as reported by [3,25]. *Xenopus dact3* showed a rather widespread expression, at gastrulation and neurulation stages labeling the primitive ectoderm, with higher expression levels in the neural plate and newly formed paraxial mesoderm (not shown). At stage 36, the gene still was expressed widely, with prominent expression in the somites (Figure 5G). In the zebrafish at 36hpf, *dact1* was expressed widely, including the craniofacial mesenchyme, the somites, the neural tube, the otic vesicle, the pectoral fin bud and the surface ectoderm (Figure 5H). A somewhat more restricted expression pattern was found for *dact2* (Figure 5I), which showed strong expression in the pharyngeal arches and the somites. *dact3a* showed a widespread expression including the hindbrain, pharyngeal arches and somites, while *dact3b* expression labeled the fore-, mid- and hindbrain, the pharyngeal arches and notochord (Figure 5J,L). *dact4* and *dact4r* displayed similar expression patterns, encompassing the brain, the otic vesicle and the pectoral fin bud (Figure 5K,M). Taken together, while individual *Dact* genes were expressed in unique, at times species-specific locations, at least one member of the Dact1/3 gene group as well as of the Dact2/4 gene group was expressed in the paraxial mesoderm, the fin/limb buds and the mesenchyme of the pharyngeal arches in all vertebrates, suggesting that these are sites of original *dact* function. The exception is *Xenopus*, where no *dact2*/*4* representative is present. Here, *dact1* has taken over *dact2* expression domains such as the emigrating cranial neural crest cells. Notably, in all species, expression domains overlapped, suggesting that *Dact* genes may regulate Tgfβ and Wnt signaling in a combinatorial fashion.

## Discussion

Dact multi-adapter proteins are important regulators at the intersection of Wnt and Tgfβ signaling [3,6,9]. The aim of this study was to shed light on the evolution of *Dact* genes and their functional domains and motifs. Here, we identified previously unknown *dact* genes and show that they arose late in the deuterostome lineage. In gnathostomes, four *Dact* genes were generated after 2R, and in many extant species, these four genes are still present. The distribution of functional domains and protein motifs suggests that the ancestral Dact function lied with Wnt signaling; a role in Tgfβ signaling may have emerged later. Motif reduction in particular in the newly identified Dact4 suggests that this protein may counteract the function of the other Dacts. Significantly, many *Dact* genes are co-expressed during development. Hence, the complement of Dact proteins present in a given tissue will determine the outcome of Wnt and Tgfβ signaling events.

### Gnathostomes were originally equipped with four *Dact* paralogs

Previous studies identified *Dact1,2,3* genes in mouse and humans, a *Dact1* and *2* gene in chicken, one *dact1* gene in frogs (duplicated in the pseudotetraploid *Xenopus laevis*), and a *dact1* and *2* gene in zebrafish [3,4,24-28]. Performing extensive database searches, we identified numerous gnathostome *Dact* genes: four distinct *Dacts* were identified in chondrichthyans; for actinopterygian bony vertebrates, we found five *dacts* in holosts and four to six in teleosts, and for sarcopterygians, we found four *Dacts* in *Latimeria* as well as in anapsid and diapsid reptiles, two in birds, two in amphibians and three in mammals. The phylogenetic analysis of Dact proteins, protein motif comparison and genomic synteny analysis revealed that all these Dacts belong to four paralog groups that arose after 2R rather than by individual gene duplication events. Subsequently, specifically in the tetrapod lineage individual *Dact* genes were lost, with mammals shedding *Dact4*, birds loosing *Dact3* and *Dact4*, and amphibians loosing *dact2* and *dact4*. The presence of *Dact4* in the two reptile lineages and the conservation of the *Dact4* gene locus in mammals and frogs suggest that in tetrapods, this newly discovered gene persisted well after the split of the amphibian and the various amniote lineages, and was independently shed in frogs, birds and mammals.

### During the vertebrate 2R, *Dact1/3* arose from one and *2/4* from the other precursor

**Figure 6:**
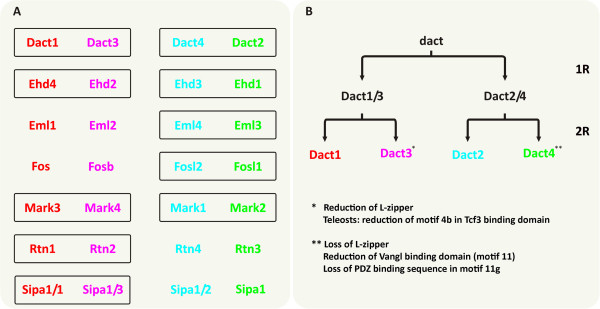
**Summary of the phylogenetic analysis of gnathostome *Dact *and *Dact *associated genes. (A)** Grouping of gnathostome *Dact* and *Dact* associated genes as suggested by the phylogenetic analysis of the cognate protein sequences. Genes genomically colocalizing with a particular *Dact* gene are depicted in the same color as the associated *Dact* gene. Black boxes link genes that form a well-supported metagroup in the corresponding phylogenetic tree (Additional file 7). Note that in all cases, *Dact1/3* and/or *Dact2/4* associated sequences were grouped. **(B)** Model for the evolution of gnathostome *Dacts*. The pairwise grouping of *Dact1/3* and *Dact2/4* and their associated genes suggests that after the first vertebrate genome duplication (1R), a *Dact1/3* and a *Dact2/4* precursor was generated, which during the 2R gave rise to the individual *Dact1*, *Dact3*, *Dact2* and *Dact4* genes. Subsequently, in Dact3 the leucine zipper required for Dact dimerization was reduced. Moreover, in teleosts, motif 4b located in the center of the Tcf3 interacting region was reduced (dact3b) or eliminated (dact3a). In Dact4, the leucine zipper as well as the PDZ binding domain of motif 11 g was lost, motifs 11d-f (Vangl binding domain) were reduced, and motifs 6 and 10 were gained. This suggests that Dact3 and, more prominently, Dact4 proteins have altered molecular properties compared to Dact1, Dact2, and the original dact.

The analysis of Dact proteins sequences revealed a number of motifs that distinguish individual Dacts. However, we also found motif or motif variations that suggest a particularly close relationship of Dact1/3 and Dact2/4. In phylogenetic tree analyses, Dact1 and Dact3 proteins formed a metagroup, and Dact2/4 formed another metagroup. Phylogenetic trees constructed for genes that have paralogs at each of the four Dact loci showed the same topology as the Dact trees (summarized in Figure 6A). Metagroups linking *Dact1*/*2*; *Dact1*/*4*; *Dact3*/*2* or *Dact3*/*4* and associated genes were never observed. Moreover, the pair-wise grouping of Dact1/3 and Dact2/4 sequences as well as the sequences from Dact1/3- or Dact2/4-associated genes was supported by high bootstrap values. This suggests that *Dact1/3* arose from one ancestor and *Dact2/4* from the other ancestor that had been generated during 1R (summarized in Figure 6B).

### Tracing the teleost *dacts*

In teleost fish, the genome was duplicated a third time (3R, [21,22]). However, we were only able to identify single *dact1* and *2* genes, located in a conserved, *dact1*- and *dact2*-specific genomic environment, respectively. This suggests that immediately after the 3R and before the radiation of teleosts, one of the *dact1*and *dact2* genes was shed. In pufferfish, while the *dact1* locus environment was clearly recognizable, the *dact1* gene itself was absent, suggesting a more recent gene loss. In contrast to *dact1* and *dact2*, consistently two genes and gene loci were found for teleost *dact3* (possible exception: stickleback). In phylogenetic trees, the dact3a and 3b protein sequences formed well supported subgroups. Moreover, *dact3a* and *dact3b* loci were related but clearly distinguishable. This suggests that teleosts kept both *dact3* genes and gene loci that were generated during 3R. Interestingly, two *dact4* genes were found in the gar and the zebrafish. The first gene closely resembled the *Dact4* of other vertebrates and consisted of the typical 4 exons. The second gene was intronless. It resided in a similar genomic environment in the gar and the zebrafish, but this environment was unrelated to that of the first *dact4* gene. Significantly, the gar is a holost fish that has not undertaken the teleost-specific 3R [22,23]. Together, this suggests that the second *dact4* is a retrotranscribed gene (hence called *dact4r*) that appeared in actinopterygians before the holost-teleost split, and, together with the genuine 3R-derived *Dact4b*, was eliminated in all teleosts analyzed here except cyprinids.

### *Dact* genes evolved late in the deuterostome lineage

Dact proteins are important regulators of Wnt and Tgfβ signal transduction. Yet these signaling pathways evolved prior to the split of deuterostome and protostome lineages [1,2]. This seems at odds with the current view that *Dact* genes are specific for bony vertebrates [3,4,24-28]. Our study for the first time identified dact sequences in cyclostome vertebrates and in non-vertebrate chordates. However, despite intensive searches, no dact sequences were found outside chordates, suggesting that *dact* genes appeared late in the deuterostome lineage. In the cyclostomes *Petromyzon marinus* and *Lethenteron japonicum*, our searches identified several genomic fragments encoding aa sequences with homology to gnathostome *dacts*. As some of these fragments were unlinked, it was not possible to determine the exact number of *dact* genes present in cyclostomes. However, at least four distinct *dacts* could be clearly distinguished. Currently, it is controversial whether cyclostomes and gnathostomes shared the first round of genome duplication, whether an independent genome duplication occurred in the cyclostome lineage, or whether individual genes were duplicated [32-34]. While most of the phylogenetic trees rather support independent expansions of the Dact family in cyclostomes and gnathostomes, the star-like topology shown by quartet puzzling indicates the uncertainty of their relationship. For non-vertebrate chordates, we were able to identify a *dact* gene in the Florida lancelet, but not in any of the tunicates searched. This is remarkable, given that tunicates are thought to be more closely related to vertebrates than cephalochordates [35]. However, tunicates have reduced their body plan during evolution, and it is possible that they secondarily lost their *dact* gene. We can speculate that the loss of signaling cascades regulators may have facilitated the reduction of tunicate body structures.

### The original chordate *dact* may have served in Wnt signaling

Comparing the presence and distribution of functional domains and proteins motifs we found that a number of these, but not all, were shared by Dacts from gnathostomes, cyclostomes and the lancelet, including motifs 1, 2a-f, 3c, 4b, 5a-c, 8b, 11e-f, and the basic aa of the C-terminal motif 11 g. Thus, these motifs may represent the original repertoire of the ancestral dact. Motifs 1–5 occupy the N-terminal half of Dact proteins and encompass the leucine zipper essential for homo- and heterodimerization, a functionally characterized and a further predicted nuclear export signal, a domain that assists binding to Dvl and a domain that in gnathostome Dact1 has been implicated in Tcf3 binding [9,12,13], and this study). The motifs located in the C-terminal half provide a functionally characterized nuclear localization signal (motif8b) and contribute to the Vangl binding domain (motifs 11e,f; [8,9,12], and this study). All proteins are enriched with serines, particularly in the area containing motifs 2f, 11e. This suggests that already the ancestral dact was a multiadaptor protein, capable of interacting with molecules in the β Catenin dependent and PCP Wnt signaling pathway, possibly able to shuttle between the nucleus and cytoplasm, and subject to extensive regulation by phosphorylation.

In gnathostomes Dacts 1,2,3, motif 11 g contains the K-L/V-MTTV sequence, a PDZ binding domain required for the interaction of Dact with Dvl [3-5,9]. This motif was also found in cyclostome dactA, B and D, suggesting that it was a feature of the Dact protein in the last common ancestor of vertebrates. In contrast, the lancelet motif 11 g does not contain a recognizable PDZ binding motif. Thus, either *Branchiostoma* dact has secondarily lost this sequence, or alternatively, this sequence appeared in the vertebrate lineage. Consequently, it cannot be decided when the main Dvl-interacting ability of Dacts emerged during evolution. However, this function was firmly established in the vertebrate lineage.

In addition to the PDZ binding domain, a number of further motifs (3a-b, 4a, 7b-c, 9, 11a-d) are found in gnathostome and cyclostome Dacts, suggesting that they arose in the vertebrate lineage. Motif 4a resides in the Tcf3 binding domain, and motif 11d maps to the region implicated in Vangl binding [8,9,17]. Thus, it is possible that these vertebrate-specific motifs improved the ability to control Wnt signaling events. Gnathostome proteins exhibit some additional motifs (8a, 8c), and the region encompassing motifs 2a-f and 11a-g is strongly conserved. This suggests that the stabilized protein domains carry out essential molecular roles. Unfortunately, the gnathostome-specific sequence motifs have not been functionally characterized.

### The ability to inhibit Alk5 may have evolved with *Dact2/4* genes

Functional studies on mammalian and zebrafish *Dact2* showed that this molecule can regulate both Wnt and Tgfβ signaling [6,7,18]. The corresponding test has not been carried out for Dact1,3; however, in binding assays using mouse Dact proteins, only Dact2 showed significant Alk5 affinity [9]. Interestingly, the region that was implicated in Dact2-Alk5 interaction is very similar in all Dact2 and 4 proteins. Moreover, this region contains motif 3a which is absent in Dact1/3 proteins. Furthermore, gnathostome Dact2/4 have secondarily lost the S-P rich motif 4a in the Tcf3 binding domain and motif 5b in the internal Dvl binding domain. Molecular studies are required to test whether these differences account for the ability of Dact proteins to interact with Alk5. However, it is quite possible that the ability to regulate Tgfβ signaling evolved with or was stabilized in the ancestor of Dact2/4, at the expense of some functions in the Wnt signaling system.

### Could the gnathostome *Dact4* be an “anti-Dact”?

It has been recognized that after the two (teleosts: three) rounds of gnathostome genome duplications, re-diploidization occurred for many genes, but duplicated genes involved in signaling were preferentially retained. This has been interpreted as an evolutionary platform to increase complexity [2]. However, immediately after these duplication events, biological systems are potentially deregulated and instable. After the 2R, the ancestral gnathostome had four *Dact* genes, all possibly interfering with Wnt signaling. Moreover, with the duplication of *Dact2/4*, possibly also the capacity to inhibit Tgfβ signaling was enhanced. Furthermore, in the actinopterygian lineage, the *dact4r* gene appeared, potentially further destabilizing the system. How did vertebrates cope with this?

In a number of gnathostome lineages, *Dact* genes were shed: mammals lost *Dact4*, birds lost *Dact3* and *4*, frogs lost *dact2* and *4* (remarkably, *Xenopus dact3* is rather divergent and may have taken over some *dact2* function), teleosts lost the duplicated *dact1* (pufferfish lost both *dact1* copies), *dact2*, *dact4*, and most also lost *dact4r*. In animals that kept a complement of *Dact1, 2, 3*, the Dact3 leucine zipper was reduced or incapacitated, thus inhibiting the ability to dimerize. In teleost dact3 proteins, the motif 4b in the Tcf3 binding domain was reduced (dact3b) or removed (dact3a), possibly reducing Tcf3-binding capacity. Furthermore, in most (exception: zebrafish) *dact3b* genes the 3^rd^ exon was lost. Thus, specifically in teleosts, *dact3* genes may have evolved into a less potent version of *dact1*.

Amongst gnathostome *Dacts*, however, *Dact4* is the most derived. The protein lost (motifs 2b,c, 7b,c, 8a-c, 9, 11d,f), modified (motifs 2a, 3a, 11a,b,c,e,g) and gained (motifs 6, 10) a number of motifs. Significantly, the lost motifs encompass the leucine zipper; thus, the proteins are unable to dimerize. The modified motifs encompass the internal and the C-terminal (loss of the MTTV sequence) Dvl binding domain, and hence, Dact4 proteins may be unable to regulate this key molecule essential for all Wnt pathways. Since some motifs have been maintained and new motifs have been stabilized, we can assume, however, that the protein is able to carry out some protein-protein interactions. This may lead to a sequestering of Dact-interacting proteins, and hence the antagonization of Dact1,2,3 function.

### The combinatorial expression of *Dact* genes may determine the outcome of Wnt and Tgfβ signaling events in gnathostomes

In addition to gene loss or sub- and neo-functionalization, duplicated genes may diversify at the level of their cis-regulatory sequences, leading to expression divergence [2]. However, our expression analysis of mouse (*Dact1,2,3* genes), chicken (*Dact1,2* genes only), *Xenopus* (*dact1*,*3* genes only) and zebrafish *dacts* (*dact1,2,3a,3b,4,4r*) suggests that at the pharyngula- early somite stage of development (the vertebrate phylotypic stage, [30]), *Dact* genes are co-expressed in many tissues. Notably, most *Dact1* and *2* genes, and where present, *Dact3/dact3a* genes were expressed in the paraxial mesoderm, the fin/limb buds and the craniofacial mesenchyme and pharyngeal arches ([3,4,24-28]; this study), suggesting that they are the sites of original *Dact* function before the split of the *Dact1/3* and *Dact2/4* groups. This coexpression furthermore suggests that in a given tissue, the outcome of Wnt and Tgfβ signaling events depends on the combinatorial activity of these *Dacts*.

In the zebrafish, *dact3b* and *dact4* genes are mainly expressed in the brain, nevertheless still labeling the pharyngeal arches (*dact3b*) and the pectoral fin buds (*dact4, 4r*). The latter is remarkable since the expression of a retrotranscribed gene depends on the regulatory elements present at the integration site. It could be speculated that this potential anti-dact has been kept since, together with the original *dact4*, it may counterbalance the function of the numerous dact1-3 gene products. However, the net outcome of *Dact* function in mouse and chicken (few *Dacts*, no potential anti-*Dact*) and in the fish (many *dacts*, but potentially counterbalanced by *dact4* and *4r*) may be similar.

## Conclusions

This study traced the evolution of *Dact* genes and with it, the evolution of a molecular system that allows the simultaneous control of Wnt and Tgfβ signaling. Our study suggests that *Dacts* are chordate specific, with gnathostome *Dact1/3* having arisen from one, *Dact2/4* from the second precursor generated after 2R. The protein motifs present in the lancelet and gnathostome Dacts suggest that while the control of Wnt signaling may have been the ancestral role of these proteins, the ability to inhibit Tgfβ signaling may have evolved with the gnathostome *Dact2/4* precursor. Moreover, our study raises the possibility that in those vertebrates that kept Dact4, this protein may inhibit the function of the other Dacts. Our study provides the basis for structural and molecular biologists to systematically test the function of the shared and divergent Dact protein motifs, and for cell and developmental biologists to explore the combinatorial aspects of Dact function.

## Methods

### Database searches

Genomes of humans, mouse, cattle, dog, African elephant, opossum, platypus, chicken, turkey, zebrafinch, duck, budgerigar, Anole lizard, Western painted turtle, Chinese soft shield turtle, *Xenopus tropicalis*, coelacanth, spotted gar, zebrafish, Atlantic cod, Medaka, Fugu, Tetraodon, stickleback, Nile Tilapia, Southern platyfish, sea lamprey, *Ciona intestinalis, Ciona savignyi, Drosophila melanogaster, Caenorhabditis elegans, Saccharomyces cerevisiae* were searched using the Ensembl browser (http://www.ensembl.org/index.html; genome editions 2012 and 2008). Genomes of the Burmese python, *Oikopleura dioica, Branchiostoma floridae, Saccoglossus kowalevskii, Strongylocentrotus purpuratus, Aplysia californica, Tribolium castaneum, Bombyx mori,Caenorhabditis briggsae, Loa loa* and of the groups Kinetoplastida including Trypanosoma and Fungi were searched using the NCBI browser (http://www.ncbi.nlm.nih.gov/; 2011 genome editions). The genomes of the elephant shark and the Japanese lamprey were searched at the respective genome project portals (http://esharkgenome.imcb.a-star.edu.sg/ and http://jlampreygenome.imcb.a-star.edu.sg/). EST databases for the above species and for *Xenopus laevis,* and for the taxonomical groups lungfish, chondrosts, holosts, teleosts, chondrichthyans, cyclostomes, ascidians, protostomes and for protists were performed, using the NCBI browser. The first round of TBLASTN searches were performed using the human and mouse Dact1,2,3; chicken Dact1,2; *Xenopus laevis* dact1a,1b and zebrafish dact1,2 protein sequences as queries. Subsequently, we also used the newly identified zebrafish, lizard and turtle Dact3 and Dact4 sequences, the lamprey and the *Branchiostoma* sequences. Moreover, we performed searches with protein sequences encoded by individual exons and with protein motifs. Fgenesh [36] was used to predict the exon structure for sequences where no annotation was available.

### Molecular phylogenetic analyses

For molecular phylogenetic analyses, protein sequences were aligned using ClustalW [37] and T-Coffee [38]. The alignment was optimized manually using BioEdit [39], using information from pairwise alignments and the position of functionally significant amino acids (Additional file 13). The resulting alignment had large gaps, and many regions outside identifiable conserved motifs could not be aligned unambiguously. Using the ‘automated1’ and ‘strict’ settings of trimAl [40] as a guide, non-significant residues were removed manually (Additional file 14). The most suitable evolution model for the alignment was determined by using ProtTest3 [41] as JTT + G + F. The JTT model was used in all subsequent analyses.

Phylogenetic tree reconstruction was carried out employing a variety of methods. Maximum Likelihood analyses were carried out using PhyML 3.0 [42] with bootstrap analysis (100 repeats) on the phylogeny.fr server [43], and by using IQTree [44] with fast bootstrap analysis (1000 repeats). Bayesian MCMC sampling (100,000 generations) was carried out using MrBayes 3.2 [45] with model averaging (resulting in selection of the JTT model). For tree reconstruction using quartet puzzling, Tree-Puzzle 5.2 [46] was used with 100,000 puzzling steps. Tree-Puzzle was in addition used for likelihood mapping. The resulting trees were visualized using iTOL [47]. Consensus sequences of the untrimmed, gapped alignments were generated using WebLogo [48].

### Motif prediction

To identify potential functional domains in the Dact proteins, protein sequences were searched using PSort [49] and NetNes 1.1 [50].

### Embryos and *in situ* hybridization

Fertilized chicken eggs (Winter Farm, Royston) were incubated in a humidified atmosphere at 38.5°C. Embryos were staged according to [51]. Mice were obtained from the UoP animal resource centre and mated overnight. The appearance of a vaginal plug the next morning was taken as day 0.5 of development (E0.5). Zebrafish embryos (Biological Services Unit, King’s College London) were raised at 28°C in egg water (0.3 gl/l Instant Ocean Salt, 1 mg l/l Methylene Blue; after 24hpf supplemented with 0.2 mM 1-phenyl-2-thiourea (PTU, Sigma) to prevent pigmentation) and staged according to [52]. All animal experiments were conducted following the UK Animals (Scientific Procedures) Act and have been approved of by UoP AWERB (Reference No. 14005).

Embryos were harvested in 4% PFA and subjected to in situ hybridization as described in [27] (mouse and chicken embryos) and [53] (zebrafish embryos). Probes for mouse *Dact* genes were kindly provided by R. Suriben [27], chicken *Dact1* and *Dact2* probes are detailed in [27]. Probes for *Xenopus dact1a* and *dact3*/ scaffold 110 were amplified using the primers Xd1F 5’-CCGGGAGCGCCTGGAGG-3’ and Xd1R 5’-AGTTCATTGACATTACAAGAAGG-3’ and Xd3F 5’-GGTGGTGACCGAGGGCG-3’ and Xd3R 5’-CCTGTGTGAAATCTCATGATC-3’, respectively. The *dact1* probe recognizes *dact1a* and *b*, the *dact3* probe recognized *dact3* derived from both scaffold 110 and from scaffold 13803. Probes for zebrafish *dact1* and *dact2* were synthesized using PCR products obtained from 36hpf embryo cDNAs, which were amplified using a gene specific forward primer and a reverse primer containing the T7 promoter sequence in addition to gene specific region. The sequences of the primers used are: zfdact1F 5’- GTTGCTTAGGAAACAGTTGAA-3’, zfdact1R 5’- *TAATACGACTCACTATAGGGAG*AGATGATGTCTGGGAGCCTAC-3’; zfdact2F 5’- TGGTGGTTCAGGCTCATTGT-3’ and zfdact2R 5’- *TAATACGACTCACTATAGGGAGA*GTTGAGGTCCATTCAGCGAT-3’. Probes for zebrafish *dact3a, 3b, 4, 4r* were obtained from the plasmids IMAGp998P2412045Q (*dact3a*), IMAGp998G1214848Q (*dact3b*); IMAGp998F2414609Q (*dact4*); IMAGp998I1217623Q (*dact4r*) obtained from Source Bioscience.

## Availability of supporting data

The data sets supporting the results of this article are available in the Treebase repository, http://purl.org/phylo/treebase/phylows/study/TB2:S15970 [54].

## Competing interests

The authors declare that they have no competing interests.

## Authors’ contributions

All authors contributed to the collection, analysis and interpretation of the data. The laboratory work was performed by DRS and SD, the phylogenetic analyses by FRS, the synteny analysis by SD, LEA, DRS and RGJ. SD and LEA conceived the design of this study. SD wrote the manuscript with contribution of LEA and FRS. All authors have read and approved the final manuscript.

## Supplementary Material

Additional file 1 **Bilaterians searched for *Dact *** **genes.** Click here for file

Additional file 2 Accession numbers of the Dact sequences analyzed in this work. Click here for file

Additional file 3 **Alignment of cyclostome and *Branchiostoma *** **dact proteins.** Click here for file

Additional file 4 Rooted phylogenetic trees for chordate Dact proteins. Click here for file

Additional file 5 Unrooted phylogenetic tree for gnathostome Dact proteins. Click here for file

Additional file 6 **Extended synteny analysis of gnathostome *Dact *** **gene loci.** Click here for file

Additional file 7 **Phylogenetic protein trees for genes associated with all four *Dact *** **loci.** Click here for file

Additional files 8 Dact protein identity matrix. Click here for file

Additional file 9 Conserved Dact protein motifs. Click here for file

Additional file 10 Alignments of gnathostome Dact proteins. Click here for file

Additional file 11 Gnathostome Dact protein sequence logo. Click here for file

Additional file 12 Arrangement of the leucine zipper. Click here for file

Additional file 13 Alignment of Dact protein sequences. Click here for file

Additional file 14 Trimmed alignment of Dact protein sequences. Click here for file
