# Supplementary material for: Dact genes are chordate specific regulators at the intersection of Wnt and Tgf-β signaling pathways
Source: BMC Evol Biol. 2014 Aug 6;14:157. doi: 10.1186/1471-2148-14-157 (PMC4236578; doi:10.1186/1471-2148-14-157)
Supplement: Additional file 1 — Bilaterians searched for Dact genes. [file 1471-2148-14-157-S1.pdf]

Additional File 1. Bilaterians searched for *Dact* genes.

Abbreviations of species names are indicated on the left; others: o, order; so, superorder. *Dact* EST sequences are represented by a tick; for genomic sequences, the genomic localization is indicated, following the nomenclature of the 2012 edition of the Ensemble (http://www.ensembl.org/index.html), NCBI (http://www.ncbi.nlm.nih.gov/), elephant shark genome project (http://esharkgenome.imcb.a-star.edu.sg/) and Japanese lamprey genome project (http://jlampreygenome.imcb.a-star.edu.sg/) databases. For the Anole lizard, *Xenopus tropicalis*, the coelacanth, and the sea lamprey, in addition the nomenclature used in the 2008 edition of Ensemble is shown.

**Deuterostomes**									
	abbreviation	species	common name	*Dact1*	*Dact2*	*Dact3 or dact3a*	*dact3b*	*Dact4*	comments
classification									
Phylum: Chordata									
**Subphylum: Vertebrata**									
Infraphylum: Gnathostomata									
Superclass: Osteichthyes									
Class: Sarcopterygii									
Subclass: Tetrapodomorpha									
Amniota; Synapsids - Infraclass: Mammals; Placentalia	Hs	*Homo sapiens*	human	chr14✓	chr6✓	chr19✓		(locus chr11)	*Dact4* locus is identifiable but lacks a *Dact* gene
	Mm	*Mus musculus*	mouse	chr12✓	chr17✓	chr7✓		(locus chr19)	
	Bt	*Bos Taurus*	cattle	chr10	chr9	chr18		(locus chr29)	
	Cf	*Canis familiaris*	dog	chr8	chr1	chr1		(locus chr18)	
	La	*Loxodonta africana*	African elephant	s2755		s4446			
Infraclass: Mammals; Marsupials	Md	*Monodelphis domestica*	opossum	chr1	chr2	chr4		(locus chr5)	
Infraclass: Mammals; Monotremes	Oa	*Ornithorhynchus anatinus*	platypus	c214, 26117; c164635 (ex4)	c20694, c22096	c69853 (ex1), c289003 (ex4)			
Amniota; Diapsids; Archosauromorpha; Infraclass: Aves	Gg	*Gallus gallus*	chicken	chr5✓	chr3✓				*Dact3* and *4* loci not conserved
	Mg	*Meleagris gallopavo*	turkey	chr5	chr2				
	Tg	*Taeniopygia guttata*	zebrafinch	chr5	chr3				
	Ap	*Anas platyrhynchos*	duck	s626	s530				
	Mu	*Melopsittacus undulatus*	budgerigar	JH556483	JH556595				
Amniota; Diapsids; Infraclass: Lepidosauromorpha	Ac	*Anolis carolinensis*	Anole lizard	GL343459 (formerly s5, s29) and chr2	chr1 (formerly s549)	GL343568 (formerly s1268)		GL343928 (formerly s71, s1206)	
	Pym	*Python molurus*	Burmese python	c26513843 c26939700	c24659227 c26563497 c26659464 c26922077	c26582260		c26563039 c26110406 c25444438 c24942884	
Amniota; Anapsids; Infraclass: Testudines (turtles and tortoises)	Cpb	*Chrysemys picta bellii*	Western painted turtle	JH584658	JH584758	JH584472		JH584543	
	Ps	*Pelodiscus sinensis*	Chinese soft shield turtle	JH212506	JH208515	JH209944		JH206113	
Infraclass: Amphibia; Anura	Xt	*Xenopus tropicalis*	Western clawed frog	GL172704 (formerly s68) ✓	(locus GL172638)	GL172667 (formerly s31) ✓		(locus GL173034, formerly s398)	Remnants of *Dact2* and *4* loci identifiable
	Xl	*Xenopus laevis*	African clawed frog	1-a, 1-b ✓		3-a, 3-b ✓			
	Rc	*Rana catesbeiana*		✓					
Subclass: Dipnoi (lungfishes)	Pa	*Protopterus aethiopicus*	African or marbled lungfish		✓				
Subclass: Coelocanthimorpha	Lc	*Latimeria chalumnae*	Coelacanth	JH126564 (formerly c002619-17)	JH129292 (formerly c230613-11)	JH128225 (formerly c190000,13,18,22)		JH126593 (formerly c015702)	
Class: Actinopterygii<br>Subclass: Neopterygii									
Infraclass: Holostei (bowfins, gars)	Lo	*Lepisosteus oculatus*	Spotted gar	LG7	LG16	LG2		LG28	Intronless *dact4r* on LG14
Infraclass: Teleostei									
o: Cypriniformes	Dr	*Danio rerio*	zebrafish	chr17✓	chr13✓	chr18✓	chr10✓	chr14✓	Intronless *dact4r* on chr24 ✓
	Pp	*Pimephales promelas*	Fathead minnow	✓		✓		✓	
o: Siluriformes	Ip	*Ictalurus punctatus*	Channel catfish	✓					

**so: Protacanthopterygii**									
o: Salmoniformes	Ssa	*Salmo salar*	Altlantic salmon	✓					
	Ot	*Oncorhynchus tshawytscha*	Chinook salmon					✓	
	Omy	*Oncorhynchus mykiss*	Rainbow trout	✓			✓		
o: Osmeriformes	Omo	*Osmerus mordax*	Rainbow smelt		✓				
**so: Paracanthopterygii**									
o:Gadiformes=Anacanthini	Gmo	*Gadus morhua*	Atlantic cod	s909/ c572298, c143687,	s609/ c360826	s3150	s4368	s3610; partial sequences on c76676	
**so: Acanthopterygii**									
o: Tetraodontiformes	Tr	*Takifugu rubripes*	Fugu	(locus on s136)	s53	s165	s455	c187	*dact1* locus present, but no *dact1* gene
	Tn	*Tetraodon nigroviridis*	Green spotted pufferfish	(locus on s7089)	chr17	chrUn/s14702	chr7	chr1	
o: Gasterosteiformes	Ga	*Gasterosteus aculeatus*	three-spined stickleback	grXV✓	grVI	grI✓	(locus on grVII)	grIV✓	Sequence gap at the position of the *dact3b* gene
o: Beloniformes	Ol	*Oryzias latipes*	Medaka	chr22✓	chr15✓	chr13	chr14✓	chr10✓	
o: Perciformes	Pf	*Perca flavescens*	Yellow perch					✓	
	Dl	*Dicentrarchus labrax*	European seabass					✓	
	On	*Oreochromis niloticus*	Nile Tilapia	GL831368	GL831145	GL831147	GL831515 ✓	GL831165	
	Hb	*Haplochromis burtani*	African cichlid				✓		
	Sau	*Sparus aurata*	Gilthead seabream					✓	
o: Cyprinodontiformes	Xm	*Xiphophorus maculatus*	Southern platyfish	JH557083	JH556663	JH556880		AGAJ01047724 & JH556834	
SuperclassChondrichthyes									
Subclass: Elasmobranchii;									
Subdivision Selachii; **so: Squalomorphi**	Sac	*Squalus acanthias*	Spiny dogfish shark	✓					
Subdivision / **so: Batoidea**	Tc	*Torpedo californica*	Pacific electric ray		✓			✓	
	Le	*Leucoraja erinacea*	Little skate http://www.marinegenomics.org/node/27111					✓	
Subclass: Holocephali; o: Chimaeriformes	Cm	*Callorhinchus milii*	Elephant shark	AAVX 014209261.1 01221604.1 01236760.1 01179725.1 01276763.1	AAVX 01057442.1 01184388.1	AAVX 01159308.1		AAVX 01316550.1	
Infraphylum: Agnatha									
Superclass: Cyclostomata									
Class Hyperoartia/Petromyzontida	Pm	*Petromyzon marinus*	Sea lamprey	dactA (exons 3+4, GL476511/c36439); dactB (exons 3+4, c37220 and c20195); dactD (exon 4, c54804)					At least 3 genes
	Lj	*Lenthenteron japonicum*	Japanese lamprey	dactA (KE993709); dactB (KE993739); dactC (exons 2-4, KE993726; similar exon 4 on KE999188/KE99520); dactD (exon 4, KE994909); orphan exon 1: APJL01152884/APJL01160608					At least 4 genes
Phylum: Chordata **Subphylum: Tunicata/Urochordata**									
Class: Appendicularia	Oik	*Oikopleura dioica*							No significant hit
Class: Ascidiacea	Cii	*Ciona intestinalis*	Vase tunicate						
	Cis	*Ciona savignyi*							
Phylum: Chordata **Subphylum: Cephalochordata** Class Leptocardii	Bfl	*Branchiostoma floridae*	Florida lancelet	s65 ✓					
Phylum: Hemichordata Class: Enteropneusta	Sak	*Saccoglossus kowalevskii*	Acorn worm						No significant hit
Phylum: Echinodermata Class: Echinoidea	Spu	*Strongylocentrotus purpuratus*	California purple sea urchin						No significant hit

**Protostomes - lophotrochozoa**									
Phylum: Mollusca Class: Gastropoda	Apc	*Aplysia californica*	California sea slug or California sea hare						No significant hit
**Protostomes - ecdysozoa**									
Phylum: Arthropoda Class: Insecta	Drm	*Drosophila melanogaster*	Fruit fly						No significant hit
	Trc	*Tribolium castaneum*	Red flour beetle						
	Bom	*Bombyx mori*	domesticated silkmoth						

Phylum: Nematoda	Cel	*C.elegans*							No significant hit
	Cbr	*C.briggsae*							
	Loa	*Loa loa*	African eye worm						
