# Supplementary material for: Dact genes are chordate specific regulators at the intersection of Wnt and Tgf-β signaling pathways
Source: BMC Evol Biol. 2014 Aug 6;14:157. doi: 10.1186/1471-2148-14-157 (PMC4236578; doi:10.1186/1471-2148-14-157)
Supplement: Additional file 2 — Accession numbers of the Dact sequences analyzed in this work. [file 1471-2148-14-157-S2.pdf]

Additional File 2. Accession information for the *Dact* genes

---

Dact1

Species	Sequence sources (location, ENSEMBL ID, Genbank ID)
Homo sapiens	chr14 ENSP00000337439
Mus musculus	chr12 ENSMUSP00000117169, NP_001177395
Bos Taurus	chr10 ENSBTAP00000025871
Canis familiaris	chr8 ENSCAFP00000022721
Loxodonta africana	super contig 2755, scaffold 9 ENSLAFP00000006293
Monodelphis domestica	chr1 ENSMODP00000010125
Ornithorhynchus anatinus	Exons 2-4: super contig 214 ENSOANP00000026117, super contig 164635 ENSOANP00000012266
Gallus gallus	chr5 ENSGALP00000019594, NP_001038157
Melleagris gallopavo	Exons 2-4: chr5 ENSMGAP00000014386
Taeniopygia guttata	chr5 ENSTGUP00000013409, XP_002200415
Anas platyrhynchus	Exons 2-4: super contig 626 ENSAPLP00000006988
Melopsittacus undulatus	JH556483
Anolis carolinensis	GL343459, scaffold 5, scaffold 29, chr2 ENSACAP00000014546, XP_003227188
Python molurus	Exons2-3: contig 26513843, contig 26939700
Chrysemys picta bellii	JH584658.1
Pelodiscus sinensis	Exons2-4: JH212506.1 ENSPSIP00000000543
Xenopus laevis	1a Q8JJ48<br>1b Q8QG92
Xenopus tropicalis	Scaffold 68 ENSXETP00000026164
Rana catesbeiana	Exon 4: EST GO472221
Latimeria chalumnae	contig 002619, contig 002618, contig 002617, JH126564 ENSLACP00000021405
Lepisosteus oculatus	LG7
Danio rerio	chr17 ENSDARP00000058000, NP_999896
Pimephales promelas	EST DT209342.1, DT203450.1
Ictalurus punctatus	Exon 4 (partial): EST FD292185.1
Salmo salar	EST DW568517, DY733227, DY706739, DW582161
Oncorhynchus mykiss	Exon1: EST CX026758, Exons 2-4: EST CX028139, CX036471, CX036458
Gadus morhua	Exon2: contig 572298/contig 143687, Exon4: scaffold 909
Gasterosteus aculeatus	grXV genomic, EST DW609376
Oryzias latipes	Exons 2-4: chr22 ENSORLP00000012735, EST AM331483
Oreochromis niloticus	GL831368 ENSONIP00000014775, probably wrong splices
Xiphophorus maculatus	Exons 2-4: JH557083 ENSXMAP00000004931 (ex3-4 no intron)
Squalus acanthias	Exon 4: EST EB688325.1
Callorhinchus milii	Exon 2: AAVX01221604, Exon 3: AAVX01236760, Exon 4: AAVX01179725, AAVX01276763

Dact2

Species	Sequence sources (location, ENSEMBL ID, Genbank ID)
Homo sapiens	chr6 ENSP00000355760
Mus musculus	chr17 ENSMUSP00000051638 NP766414
Bos Taurus	Exons 2-4: chr9 ENSBTAP00000042173
Canis familiaris	chr1 ENSCAFP00000001234 & genomic
Monodelphis domestica	chr2 ENSMODP00000029202, XP001381611.1
Ornithorhynchus anatinus	super contig 20694, super contig 22096 ENSOANP00000022342, XP001512031
Gallus gallus	chr3 ENSGALP00000018395
Meleagris gallopavo	Exons 2-4: chr2 ENSMGAP00000012895
Taeniopygia guttata	chr3 ENSTGUP00000009966; XP_002189614
Anas platyrhynchus	Exons2-4: scaffold 530, KB742905 ENSAPLP00000016017
Melopsittacus undulatus	JH556595
Anolis carolinensis	scaffold 549, contig 30595, chr1 ENSACAP00000008321, XP_003215934
Python molurus	Exons2-4: contig 24659227, contig 26563497, contig 26659464, contig 26922077
Chrysemys picta bellii	JH584758
Pelodiscus sinensis	JH208515 ENSPSIP00000007465

Protopterus aethiopicus	Exons 1-3: cDNA FL669463
Latimeria chalumnae	contig 230613, contig 230612, contig 230611, JH129292 ENSLACP00000006683
Lepisosteus oculatus	LG16
Danio rerio	chr13 ENSDARP00000078756, NP_001071262
Osmerus mordax	Exons 1-3: EST EL546145
Gadus morhua	scaffold 609 ENSGMOP00000017472, contig 360826
Takifugu rubripes	scaffold 53 ENSTRUP00000045386, SINFRUP00000135666
Tetraodon nigroviridis	chr17 ENSTNIP00000016084
Gasterosteus aculeatus	groupVI ENSGACP00000004724
Oryzias latipes	chr15, cDNA DK099130, cDNA DK090936
Oreochromis niloticus	GL831145 ENSONIP00000012881
Xiphophorus maculatus	JH556663 ENSXMAP00000008211
Torpedo californica	Exons3-4: EST EW689921
Callorhinchus milii	Exon 2: AAVX01057442, Exon 4: AAVX01184388, AAVX01179725

Dact3

Species	Sequence sources (location, ENSEMBL ID, Genbank ID)
Homo sapiens	chr19 ENSP00000375783
Mus musculus	chr7 ENSMUSP00000104133, XP_990410
Bos Taurus	chr18 ENSBTAP00000051010
Loxodonta africana	s4446 ENSLAFP00000016003
Monodelphis domestica	Exons2-4: chr4 XP_001373542
Ornithorhynchus anatinus	contig 69853 ENSOANP00000031882, contig 289003, XP_001505988
Anolis carolinensis	Exons2-4: AAWZ02039310 ENSACAP00000018986, S1268
Python molurus	Exons2-4: contig 26582260
Chrysemys picta bellii	JH584472.1
Pelodiscus sinensis	(ex4) JH209944.1
Xenopus laevis	s110<br>s13803
Xenopus tropicalis	s31, GL172662 ENSXETESTP00000009226, ESTs CX826750, BU911207
Latimeria chalumnae	contig 190000, contig 190013, contig 190018, contig 190022, JH128225 ENSLACP00000010772
Lepisosteus oculatus	LG2
Callorhinchus milii	ex4 AAVX01159308.1 rev compl
Dact3a	
Danio rerio	chr18 ENSDARP00000105187, XP_001340996, EST BM096490, EB953635
Pimephales promelas	EST DT126141, DT173940.1
Gadus morhua	Exons 2-4: scaffold 3150 ENSGMOP00000014338
Takifugu rubripes	scaffold 165 SINFRUP00000177769
Tetraodon nigroviridis	scaffold 14702 chr16 GSTENT00022091001 CAG02860 N term trimmed
Gasterosteus aculeatus	groupI ENSGACP00000008819, EST DN705293, DT983310
Oryzias latipes	chr13 UTOLAPRE05100114910, EST DK202428, AM343964, DC260713
Oreochromis niloticus	GL831147
Xiphophorus maculatus	JH556880 ENSXMAP00000000994
Dact3b	
Danio rerio	chr10 ENSDARP00000108748, XP_001345794
Oncorhynchus mykiss	3a or b, Exon 4: EST CX260816<br>3b, Exons 2,4: BX299645
Gadus morhua	Exons 1,4: s4368
Takifugu rubripes	Exons 1,2,4: scaffold 455
Tetraodon nigroviridis	chr7
Oryzias latipes	chr14, EST AM364417
Haplochromis burtani	EST DY626400
Oreochromis niloticus	Exons 1,4: GL831515, EST GR625261

Dact4

Species	Sequence sources (location, ENSEMBL ID, Genbank ID)
Anolis carolinensis	GL343928/s1206 ENSACAP00000023296, contig 40816
Python molurus	Exons2-4: contig 26563039, contig 26110406, contig 25444438, contig 24942884
Chrysemys picta bellii	JH584543
Pelodiscus sinensis	JH206113 start exon 4 probably wrong

Latimeria chalumnae	contig 015702
Lepisosteus oculatus	Exons 2-4: LG28
Danio rerio	chr14 ENSDARP00000107587 and ESTs
Pimephales promelas	EST DT209032, DT154900, DT207848
Oncorhynchus tshawytscha	EST EL555352
Gadus morhua	scaffold 3610 contig76676
Takifugu rubripes	scaffold 187
Tetraodon nigroviridis	chr1
Gasterosteus aculeatus	groupIV, EST DN714255, DT994919
Oryzias latipes	chr10 UTOLAPRE05100112289, EST BJ526132
Perca flavescens	EST GO571064
Dicentrarchus labrax	EST FM009490
Oreochromis niloticus	GL831165
Sparus aurata	AM955614
Xiphophorus maculatus	Exon 1: scaffold AGAJ01047724, Exons 2-4: JH556834
Torpedo californica	EST EW689921
Leucoraja erinacea	EST CO049720
Callorhinchus milii	Exon 4: AAVX01316550.1
Dact4r	
Lepisosteus oculatus	LG14, no introns
Danio rerio	chr24, EST DV594105

Agnathan *Dacts*

Species	Sequence sources (location, ENSEMBL ID, Genbank ID)
Petromyzon marinus	dactA Exons 3+4: contig 36439=GL476511<br>dactB Exons 3-4: super contig 37220, contig 20195<br>dactD Exon 4: contig 54804<br>Further sequences: contig 2706, contig 46758, contig 19740
Lethenteron japonicum	dactA: KE993709<br>dactB: KE993739<br>dactC Exons 2-4: KE993726 (similar exon 4 sequences on KE999188/KE99520)<br>dactD Exon 4: KE994909<br>orphan Exon 1: APJL01152884/APJL01160608
Branchiostoma floridae	cDNA_XP_002606360, EST BW773332
