# Supplementary material for: Dact genes are chordate specific regulators at the intersection of Wnt and Tgf-β signaling pathways
Source: BMC Evol Biol. 2014 Aug 6;14:157. doi: 10.1186/1471-2148-14-157 (PMC4236578; doi:10.1186/1471-2148-14-157)
Supplement: Additional file 3 — Alignment of cyclostome and Branchiostoma dact proteins. [file 1471-2148-14-157-S3.pdf]

Additional File 3. Alignment of cyclostome and *Branchiostoma* dact proteins.

Gapped alignment of cyclostome and lancelet dact sequences with the dact sequences from the coelacanth and the spotted gar.

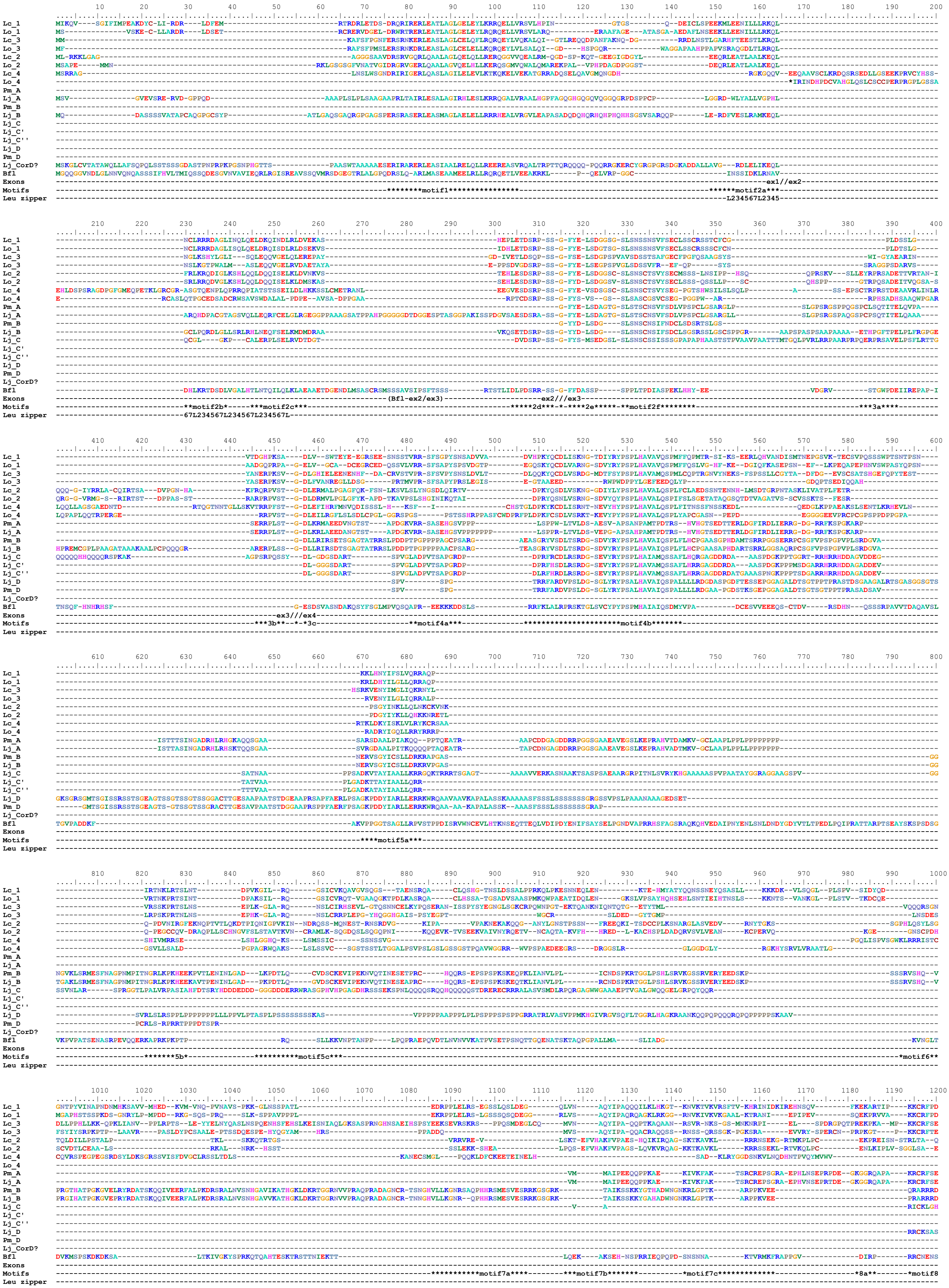
Lc_1
Lo_1
Lc_3
Lo_3
Lc_2
Lo_2
Lc_4
Lo_4
Pm_A
Lj_A
Pm_B
Lj_B
Lj_C
Lj_C'
Lj_C''
Lj_D
Pm_D
Lj_CorD?
Bfl
Exons
Motifs
Leu zipper

```
                  1210      1220      1230      1240      1250      1260      1270      1280      1290      1300      1310      1320      1330      1340      1350      1360      1370      1380      1390      1400
            ....|....|....|....|....|....|....|....|....|....|....|....|....|....|....|....|....|....|....|....|....|....|....|....|....|....|....|....|....|....|....|....|....|....|....|....|....|....|....|....|
Lc_1        DLEIN------------KKSI-KK----STSKLKK---------TNNSQIDSHHPNRQI------GLN--------------------KPISKN-----------------GH-GR---EVVLT----------------------------KPKYKR--DY--------------------------------------
Lo_1        DIPNL------------HKAS-KK----SSSKTKR---------VGSSHLDSSLAGRYP------VSTG-------------------KSAAPR---------------HHGH-GR---EVILT----------------------------KPKYKRA-DY--------------------------------------
Lc_3        EAESA------------KK-GARK----ASPRSKK---------TCRSQSENSLLNKQG------VIEKAAP----------------KYNTV---------------------DR--DEPIAG----KPP---------------------RQRRQNGGNY--------------------------------------
Lo_3        D----------------KPT-PRK-------PGRK---------ACRSQSENSLFGQRM------ALDR-------------------KYNTI---------------------DR---DGGA-----RGCQS-------------------RQRRQQPGNTCY------------------------------------
Lc_2        RVHPSKSSA--------GKGLIRRPT----YAGET---------EGRSCSESTLY-----FMEYRQPQYTDRPEPH------------KTSVNR------------------LQSS---EAACAGI--------------------------TKKKQ-------------------------------------------
Lo_2        TAPFNKHSG--------GRAVYKEHN----CVGEV---------AGRSCSQSSLY-----PAHLRQTQQVPKFGPS------------TTHR--------------------LLLP---EPRVSVDT-------------------------SKKRQT------------------------------------------
Lc_4        ---------------------------------------------------------------------------------------RKSTHR-CNSLRWVSADMDALYKGKHASR---ELVKAATLSNNY--ESAEKTNSIIKKDIFRRISLRKKPSAREDNRSRSRS-----EMNLNMAVT---KKSSALYKCESQDT
Lo_4        --------------------------------------------------------------------------------------------------------------------------------------------------------------------------------------------------------
Pm_A        DVEIIRDA---------GGQRATAAAAAATVAGGK---------ACRSQSDNSIVGRHG---RRTLPAGEASG---------------RRKDV-------------------AATA-SGATAVAAAASTTAAPPLTGTPQVQQ---------RQRRLSSH----------------------------------------
Lj_A        DVEIIRDA---------GGQRATAAVA-ATVAGGK---------ACRSQSDNSIVGRHG---RRTQPAGEASG---------------RRKDV-------------------AAAA-SGAMAAAAAAATTAAPPLTGTPQVQQ---------RQRRLSSH----------------------------------------
Pm_B        ED----------------RMQNPA--------------------AC----------------------------------------------------------------------------------------------------------------------------------------------------------
Lj_B        ED----------------RMQNPA--------------------ACAERCCRDVVSR--------ADGRDC-----------------RKSAA----------------------------AAAAGGGGR----------------------GVKRLAQQPKGH------------------------------------
Lj_C        GSWAGSLPKSLDLRGGYRHRQDDDDDHEDGAYDRRRPGNAARSSSSKRHRLREGVELVRQSFRLPGRRRRASGERASSELDVSGGG--RQPT----------------------ER--EETAGGSAAFD-----------------------YRRRLVLHPSEVRGAGAAGTRAGGH-----------------------
Lj_C'       --------------------------------------------------------------------------------------------------------------------------------------------------------------------------------------------------------
Lj_C''      --------------------------------------------------------------------------------------------------------------------------------------------------------------------------------------------------------
Lj_D        LDRVAEWRPP---PPIESAPFKRATTFGAP-GRNRGDGGGGGFGGGTG-------------RPLVST---------------------SSRSIW---------DFFAAERGKMAAAAAAECADYAAPLSV----------------------------------------------------------------------
Pm_D        --------------------------------------------------------------------------------------------------------------------------------------------------------------------------------------------------------
Lj_CorD?    --------------------------------------------------------------------------------------------------------------------------------------------------------------------------------------------------------
Bfl         HVRSTKTPSEPNTNRSESREQTTSPSVSNKPRQLKSEGRNSPCRKLQFQQDSNRMSAQQ-GQVPPGHNVPRGTEIS----------AKRCSAQA---------------STRRLSK---DAPLPQSPATPVY---GPGNDKCPAPTEF----RLSQPVVANHRPGRVPHSERYWTTGELRQQVASLNSGYAKKDE-----
Exons       --------------------------------------------------------------------------------------------------------------------------------------------------------------------------------------------------------
Motifs      b----------------***8c***---------------------*motif9*------------------------------------------------------------**motif10*----------------------------*motif11a*--------------------------------------
Leu zipper  --------------------------------------------------------------------------------------------------------------------------------------------------------------------------------------------------------

                  1410      1420      1430      1440      1450      1460      1470      1480      1490      1500      1510      1520      1530      1540      1550      1560      1570      1580      1590      1600
            ....|....|....|....|....|....|....|....|....|....|....|....|....|....|....|....|....|....|....|....|....|....|....|....|....|....|....|....|....|....|....|....|....|....|....|....|....|....|....|....|
Lc_1        --------------------CRWKSSAEIS--YEESL-----------------------------------------------------RCAR-RRQ-RREMPSIYAQ--TRFQ------------------YFSPYAYI--------------GSD-SEY--SAECESLFHSTVVDTS-----EDE--KSNYTTNC--
Lo_1        --------------------RRYKTIAEVP--YEEAM-----------------------------------------------------RKAR-RRQ-KREVLGQVSA--VYLPPTVQ--------------YASPYSYV--------------GSD-SEY--SAECASLFHSTVLDTS-----EDE--QSNYTTNC--
Lc_3        --------------------RKWRSTVEIC--QDEAAPSDSYGA---------------------------------------------QKRG--KKPHRPPAQPHSTAHG---------------------------------PRSSMYGYA--ESD-SEY--SADC-----GS-VRCAPVCEPEDD--LSSYTMNC--
Lo_3        --------------------RKWRSTLEIS--QDEGEILPGNQA---------------------------------------------PRRL--RKPPRQPPPYPYN-HPYP------------------------------------------ETDFQER--APLCRP---------------EEG-----YPTPC--
Lc_2        --------------------RKWQSAVEIS--VKAHQPSFPSSVRPGFMH----------------------------------------PKQHVRKASVLRTVSLK------------------------------THQAHYRHSAYIRSSARSDSDQSEY--SAECASLFHSTIVETS-----EDE--RSDYTTNR--
Lo_2        --------------------RKWQSVVEIS--TKPSLAPFPNSRPTG-------------------------------------------HLQVLRKAGMMRSTSLR-------------------------HRPSQYGVPRHPH-PNSYSCLLSESEVSEY--SAECASLFHSTIAESS-----EGE--LSDFTANR--
Lc_4        LSEAFGKKGKTNEWKKKPKKQKWMSVLEIS--NRT------------------PETF-------------------------------GFSQSRTRKSG-------FFSQAQAFLSR-----------------------------------------HRAF--STDCPSSFHSPILEPS-----QTD---LQNTTAHY-
Lo_4        --------------------------------------------------------------------------------------------------------------------------------------------------------------------------------------------------------
Pm_A        --------------------RRWRSTVEIC--QGAAERGLGLPSPATAAQPQWSGHAAVGQVATAVAAAQRPATMGGQAGGYGTAGRAGVRRAS-RRDAVWLQQQQSATRPFSATMGAARRP------MPVMVPVAPAALVGGGALHGSQRLV--ESD-SEY--SAECESLFRT-SFGAT--GSSDDD------------
Lj_A        --------------------RRWRSTVEIC--QGAAERGLGLPSPATAAQPQWSGHAAVGQVVTSLAAAQRPATVGGQAGGYGTAGRAGVRRAS-RREAVWLQQQQSATRPFSATMGAARRP------MPVMVPVAPAAVVGGGARHGSQRLV--ESD-SEY--SAECESLFRT-SFGAT--GSSDDD------------
Pm_B        ----------------------------------------------------------------------------------------------------------------------------------------------------------------------------------------------------NRFE
Lj_B        -------------------------SRELV--QSHGAAHGRVPAPPPPQQQPPHH-----------------------------------RRRP-APA-RVRVHPPALAHARM----------------------ARAYVTAVARADS-------DSERSEY--SAECESLFHGTVMDTS-----EDE--MSDHTTNRFG
Lj_C        --------------------RRWCSTMEIA--RGGG-----GCGN-------------------------DDDDAGGDAGTPPEPPR-WLMRAG-ARDGAWSPSWAVRGHAD---------------ASPGYPNVYPAAGCPGELLQR----------------SAIAEM---------------SED------------
Lj_C'       --------------------------------------------------------------------------------------------------------------------------------------------------------------------------------------------------------
Lj_C''      --------------------------------------------------------------------------------------------------------------------------------------------------------------------------------------------------------
Lj_D        --------------------RRLCRAGDAG--GDWAPLVGGSCGSCCCCCEEVTSC---GCPGSCDGDNDDDDDDGGGDDDGGGGGFASADRCYPSAAGFHDNRFGSEGPLQGNAGFRGDGF--CFNDGGGGNGFRGNSFHGNGFRGDGFHT---DGFHADG-FHADGFPRSGFQGHSFHGVAKLPRGSNGLRGNPMH-A
Pm_D        --------------------------------------------------------------------------------------------------------------------------------------------------------------------------------------------------------
Lj_CorD?    --------------------------------------------------------------------------------------------------------------------------------------------------------------------------------------------------------
Bfl         --------------SSRRN-KRHDGHKDSRKTQGDKRSAR---RHTTTVNTPAHMPP-------------------------------MNGLADYRN--RHSHSSTSLNGHPVSKLASNVPHGHRVPHAF-------SRGVRGLK--------KAGQSRSTSR-LYDYDT---SVLGSSS-----EE---SSSS------
Exons       --------------------------------------------------------------------------------------------------------------------------------------------------------------------(dactA-ex4/ex5)---------------------
Motifs      --------------------***motif11b*****-------------------------------------------------------**motif11c**---------------------------------------------------*************motif11d***********************---
Leu zipper  --------------------------------------------------------------------------------------------------------------------------------------------------------------------------------------------------------

                  1610      1620      1630      1640      1650      1660      1670      1680      1690      1700      1710      1720      1730      1740      1750
            ....|....|....|....|....|....|....|....|....|....|....|....|....|....|....|....|....|....|....|....|....|....|....|....|....|....|....|....|....|....|.
Lc_1        ------------------------------FGDSESSFSEVDFV---GEST---TSSDTDESGG--------LVWPQFGPSTSMQQIAVS-------------ELNNAVPKNFVKIK-----ASHNLKKKILRFRSGSLKLMTTV------
Lo_1        ------------------------------FGDSESSLSEVDFV---GEST---TSSDSDESGG--------LVWPQFGQSIGPVV--PS-------------EVTSAQAKAFVKIK-----ASHNLKKKILRFRSGSLKLMTTV------
Lc_3        ------------------------------FGDSESSLSEVESP---GFST---CSSDTDESGG--------LVWPQQVPPQL---VASS--------GSN--QSSMQP-KVFVKIK-----ASHALKKKIMRFRTGSLKVMTTV------
Lo_3        ------------------------------LGDSESSLSEADSP---GSSS---LSSDSDESGG--------LVWPQQLPPQL---A------RP-PSPSS-PPCGPQP-KAFIKIK-----ASHALKKKILRFRTGSLKVMTTV------
Lc_2        ------------------------------FGDSESSESDSDAT-SD--SS---LTLDYDDT-----DASE-FVWPEASS-QP---AASS---QPAAASVAPSKQ-AHTEPKLCRIK-----ASKALKKKIRKFQPASLKVMTMV------
Lo_2        ------------------------------FGDSESSESSDGS-SDSSLS---LNSE-D---M---EDGE-LVWAQSTV--------------GPTAAG---LPLSSRPEPPACRIK-----ASKALKKKIRRFQPAALKVMTMV------
Lc_4        ------------------------------LYGSESS---LSEVDATSCTS---LNRE---------------LIHEDRSIARPHLGE------KFFSNPLDNKNMVNGSIQLYRTK-----SFKELKKRVSRSMRPFSFKGTSSTK----
Lo_4        -------------------------------------------------------------------------------------------------------------------------------------------------------
Pm_A        -----------------------DDDDDRDEEDTDCDADDAEDA-RGG-GVDTVGPANVAEGGGRAEPA---LAGSLASSSSISSSSFFS-------------------------------------------------------------
Lj_A        -----------------------DDDDDRDEEDTDCDVDDAEDA-RGGGGGDTVGPADVAEGGGRAEPA---LTGSTATSSSISSSSFFSSSSSSSSDGPRAGGLGCVPQAQICRIK-----ASHALKKKILRCRSGSLKLMTTV------
Pm_B        KERRAPAESFRTQKFDDYDRDGDDEEEEEVATDSDKRRGRRRLQREHGFSASDEEEEDDVDTGGRQQSKGLPLAGRRGGQKAPVVAAPVAARGPEARGGG--GGGVRGQQHAACRIK-----ASRSLKKKMMHFRSGSLKVITTV------
Lj_B        DGETSASEGVPCSEVDDDYRD--DDEEEEVATDSDSDGDAGGFSVSTGSSASDEDEEDDVDNGGRQQSKGVPLAGRRGGQKAPAVAAPVAARGPEARGS----GGARGQQHAACRIK-----ASRSLKKKMMHFRSGSLKVITTV------
Lj_C        ------------------------------------DADDADEA-------DGAAGRLPADG--SAEP--------------------RSS---GSPEGLRGP----------CRVSQQVRAVPSAVKRKIRQRIADAAKTSTDV------
Lj_C'       -------------------------------------------------------------------------------------------------------------------------------------------------------
Lj_C''      -------------------------------------------------------------------------------------------------------------------------------------------------------
Lj_D        GSAGCLRDGF----LGDGYR-----GDAWRRGDARPQQGKASTGFGPGWGRPGAGVAGPQAGGG----------WWGAGPGGLG--VAGGRFN-GPGPQVSVA--HVVPVGPAGLDG------PAARRRALLRCHSVEPALTTTV------
Pm_D        -------------------------------------------------------------------------------------------------------------------------------------------------------
Lj_CorD?    -------------------------------------------------------------------------------------------------------------------------------------------------------
Bfl         --------------------------------DSEINRSDSESD---SES--------SDEAGG--------LVLA------KAKWP-AVVGTAT--------------------------------RRRL-SSQDSLSRMFPSCHLVTEL
Exons       -------------------------------------------------------------------------------------------------------------------------------------------------------
Motifs      ------------------------------*************11e*******************-------***11f***-----------------------**********motif11g************PDZ-binding------
Leu zipper  -------------------------------------------------------------------------------------------------------------------------------------------------------
```
