# Supplementary material for: Dact genes are chordate specific regulators at the intersection of Wnt and Tgf-β signaling pathways
Source: BMC Evol Biol. 2014 Aug 6;14:157. doi: 10.1186/1471-2148-14-157 (PMC4236578; doi:10.1186/1471-2148-14-157)
Supplement: Additional file 4 — Rooted phylogenetic trees for chordate Dact proteins. [file 1471-2148-14-157-S4.pdf]

Additional File 4. Rooted phylogenetic trees.

Phylogenetic trees showing the inferred relationships of Dact proteins. All trees are based on the alignment shown in Additional File 20. JTT had been determined as the most suitable model. Gamma distribution shape parameter (G) using four rate categories and the proportion of invariable sites (I) were estimated by the respective programme. The trees are rooted from the *Branchiostoma* Dact sequence.
(A) Tree reconstruction by quartet puzzling using Tree Puzzle 5.2, log likelihood -17586. Numbers indicate likelihood values. (B) Maximum likelihood tree using PhyML 3.0, log likelihood -17300. Numbers indicate bootstrap values. (C) Bayesian tree using MrBayes 3.2, log likelihood -17271. Numbers indicate credibility values. (D) Maximum likelihood tree using IQ-Tree, log likelihood -17130. Numbers indicate bootstrap values.
All trees support the Dact1/3-Dact2/4 dichotomy. In addition, trees B-D indicate possible monophyly of the cyclostome dactA-D genes, though the quartet puzzling highlights that this branch is ambiguous.

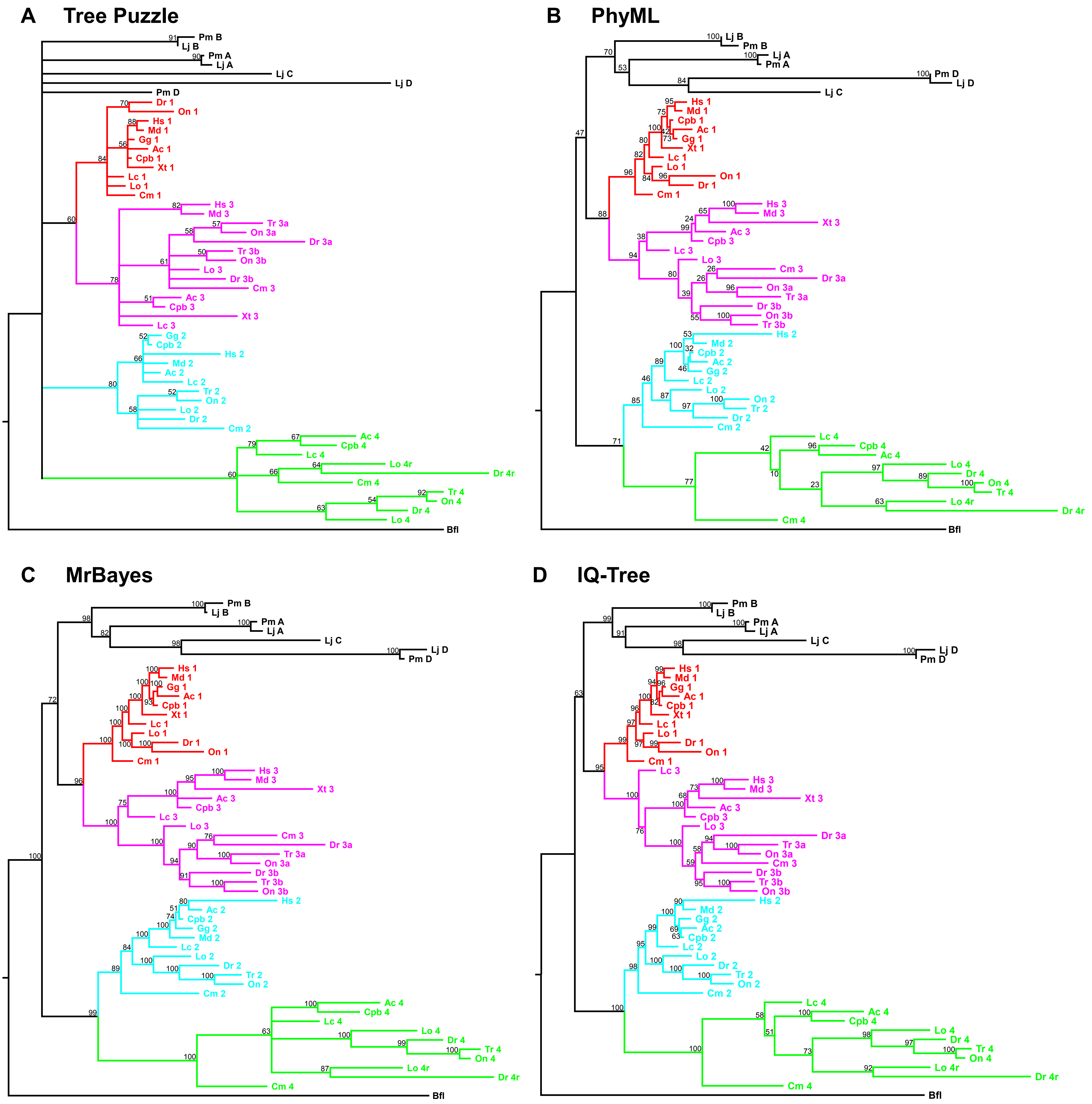
A Tree Puzzle
Pm B
Lj B
Pm A
Lj A
Lj C
Lj D
Pm D
Dr 1
On 1
Hs 1
Md 1
Gg 1
Ac 1
Cpb 1
Xt 1
Lc 1
Lo 1
Cm 1
Hs 3
Md 3
Tr 3a
On 3a
Dr 3a
Tr 3b
On 3b
Lo 3
Dr 3b
Cm 3
Ac 3
Cpb 3
Xt 3
Lc 3
Gg 2
Cpb 2
Hs 2
Md 2
Ac 2
Lc 2
Tr 2
On 2
Lo 2
Dr 2
Cm 2
Ac 4
Cpb 4
Lc 4
Lo 4r
Dr 4r
Cm 4
Tr 4
On 4
Dr 4
Lo 4
Bfl
B PhyML
Lj B
Pm B
Lj A
Pm A
Pm D
Lj D
Lj C
Hs 1
Md 1
Cpb 1
Ac 1
Gg 1
Xt 1
Lc 1
Lo 1
On 1
Dr 1
Cm 1
Hs 3
Md 3
Xt 3
Ac 3
Cpb 3
Lc 3
Lo 3
Cm 3
Dr 3a
On 3a
Tr 3a
Dr 3b
On 3b
Tr 3b
Hs 2
Md 2
Cpb 2
Ac 2
Gg 2
Lc 2
Lo 2
On 2
Tr 2
Dr 2
Cm 2
Lc 4
Cpb 4
Ac 4
Lo 4
Dr 4
On 4
Tr 4
Lo 4r
Dr 4r
Cm 4
Bfl
C MrBayes
Pm B
Lj B
Pm A
Lj A
Lj C
Lj D
Pm D
Hs 1
Md 1
Gg 1
Ac 1
Cpb 1
Xt 1
Lc 1
Lo 1
Dr 1
On 1
Cm 1
Hs 3
Md 3
Xt 3
Ac 3
Cpb 3
Lc 3
Lo 3
Cm 3
Dr 3a
Tr 3a
On 3a
Dr 3b
Tr 3b
On 3b
Hs 2
Ac 2
Cpb 2
Gg 2
Md 2
Lc 2
Lo 2
Dr 2
Tr 2
On 2
Cm 2
Ac 4
Cpb 4
Lc 4
Lo 4
Dr 4
Tr 4
On 4
Lo 4r
Dr 4r
Cm 4
Bfl
D IQ-Tree
Pm B
Lj B
Pm A
Lj A
Lj C
Lj D
Pm D
Hs 1
Md 1
Gg 1
Ac 1
Cpb 1
Xt 1
Lc 1
Lo 1
Dr 1
On 1
Cm 1
Lc 3
Hs 3
Md 3
Xt 3
Ac 3
Cpb 3
Lo 3
Dr 3a
Tr 3a
On 3a
Cm 3
Dr 3b
Tr 3b
On 3b
Hs 2
Md 2
Gg 2
Ac 2
Cpb 2
Lc 2
Lo 2
Dr 2
Tr 2
On 2
Cm 2
Lc 4
Ac 4
Cpb 4
Lo 4
Dr 4
Tr 4
On 4
Lo 4r
Dr 4r
Cm 4
Bfl
