# Supplementary material for: Dact genes are chordate specific regulators at the intersection of Wnt and Tgf-β signaling pathways
Source: BMC Evol Biol. 2014 Aug 6;14:157. doi: 10.1186/1471-2148-14-157 (PMC4236578; doi:10.1186/1471-2148-14-157)
Supplement: Additional file 5 — Unrooted phylogenetic tree for gnathostome Dact proteins. [file 1471-2148-14-157-S5.pdf]

Additional File 5. Unrooted phylogenetic tree for gnathostome Dact proteins.

Phylogenetic, maximum likelihood tree of Dact protein sequences from humans (Hs), opossum (Md), chicken (Gg), Anole lizard (Ac), the Western painted turtle (Cpb), *Xenopus tropicalis* (Xt), coelacanth (Lc), the spotted gar (Lo), zebrafish (Dr), Fugu (Tr), Tilapia (On), and elephant shark (Cm).

Asterisks indicate sequences with only partial information. Bootstrap values are indicated for all branch points (100 repeats). Sequences are annotated using the abbreviation for the species, followed by the Dact ortholog number. The gnathostome paralog groups are colour-coded: Dact1 (red), Dact3 (pink), Dact2 (turquois) and Dact4 (green). The division between the Dact1/3 and Dact2/4 metagroups is well supported with a bootstrap value of 100%.

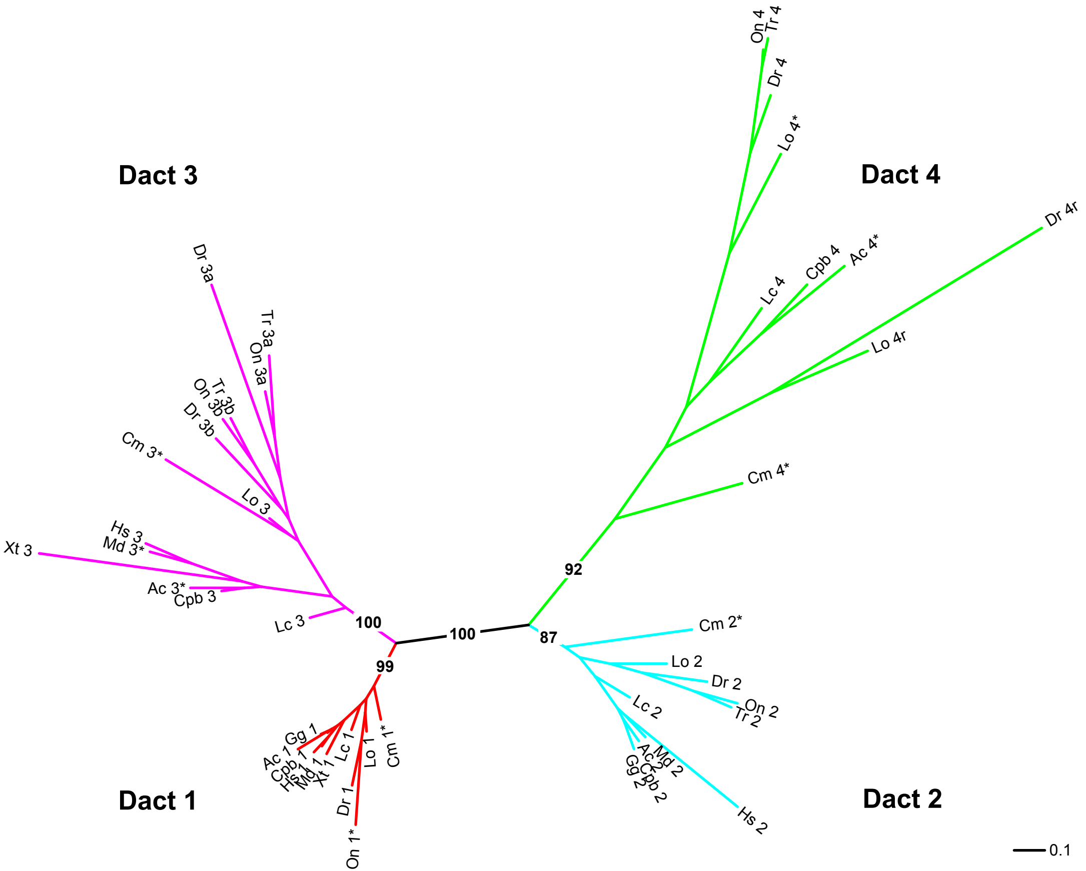

Dact 3
Dact 4
Dact 1
Dact 2
Dr 3a
Tr 3a
On 3a
Tr 3b
On 3b
Dr 3b
Cm 3*
Lo 3
Hs 3
Md 3*
Xt 3
Ac 3*
Cpb 3
Lc 3
100
100
99
92
87
On 4
Tr 4
Dr 4
Lo 4*
Dr 4r
Lc 4
Cpb 4
Ac 4*
Lo 4r
Cm 4*
Cm 2*
Lo 2
Dr 2
On 2
Tr 2
Lc 2
Md 2
Ac 2
Cpb 2
Gg 2
Hs 2
Gg 1
Ac 1
Cpb 1
Hs 1
Md 1
Xt 1
Lc 1
Lo 1
Cm 1*
Dr 1
On 1*
0.1
