# Supplementary material for: Dact genes are chordate specific regulators at the intersection of Wnt and Tgf-β signaling pathways
Source: BMC Evol Biol. 2014 Aug 6;14:157. doi: 10.1186/1471-2148-14-157 (PMC4236578; doi:10.1186/1471-2148-14-157)
Supplement: Additional file 6 — Extended synteny analysis of gnathostome Dact gene loci. [file 1471-2148-14-157-S6.pdf]

Additional File 6. Extended synteny analysis of gnathostome Dact gene loci.

Wider environment of gnathostome *Dact* loci, using the same shape and color codes as in Figure 3. Note the extended similarity of genomic loci for the individual *Dact* orthologs.

A. Dact1 loci

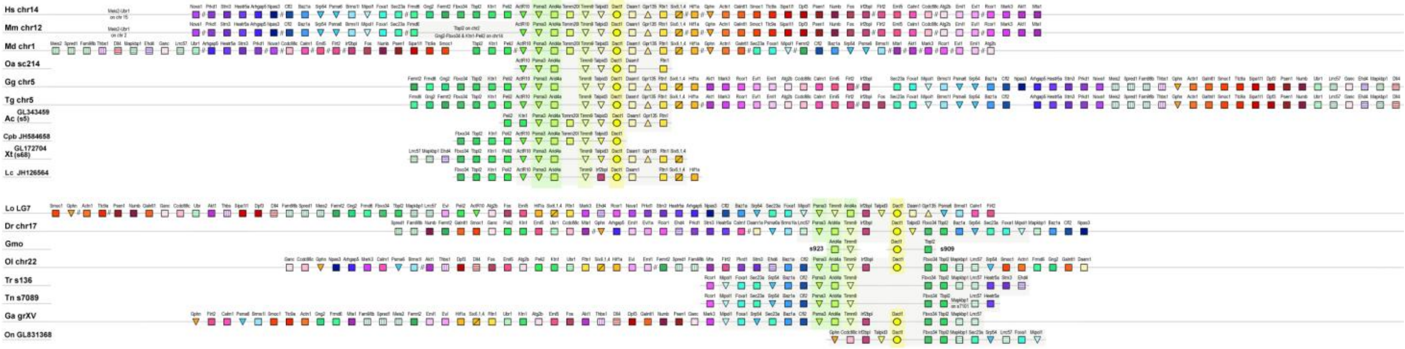

B. Dact2 loci

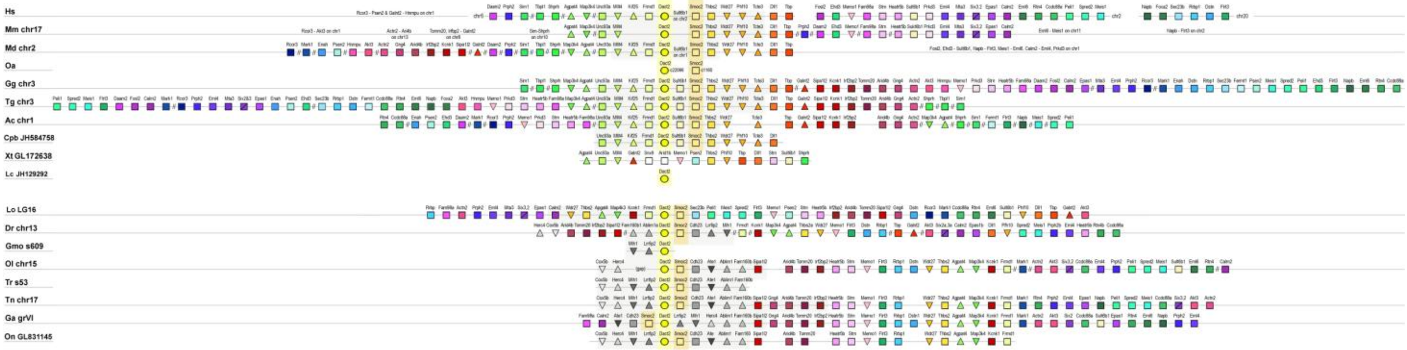

C. Dact3 loci

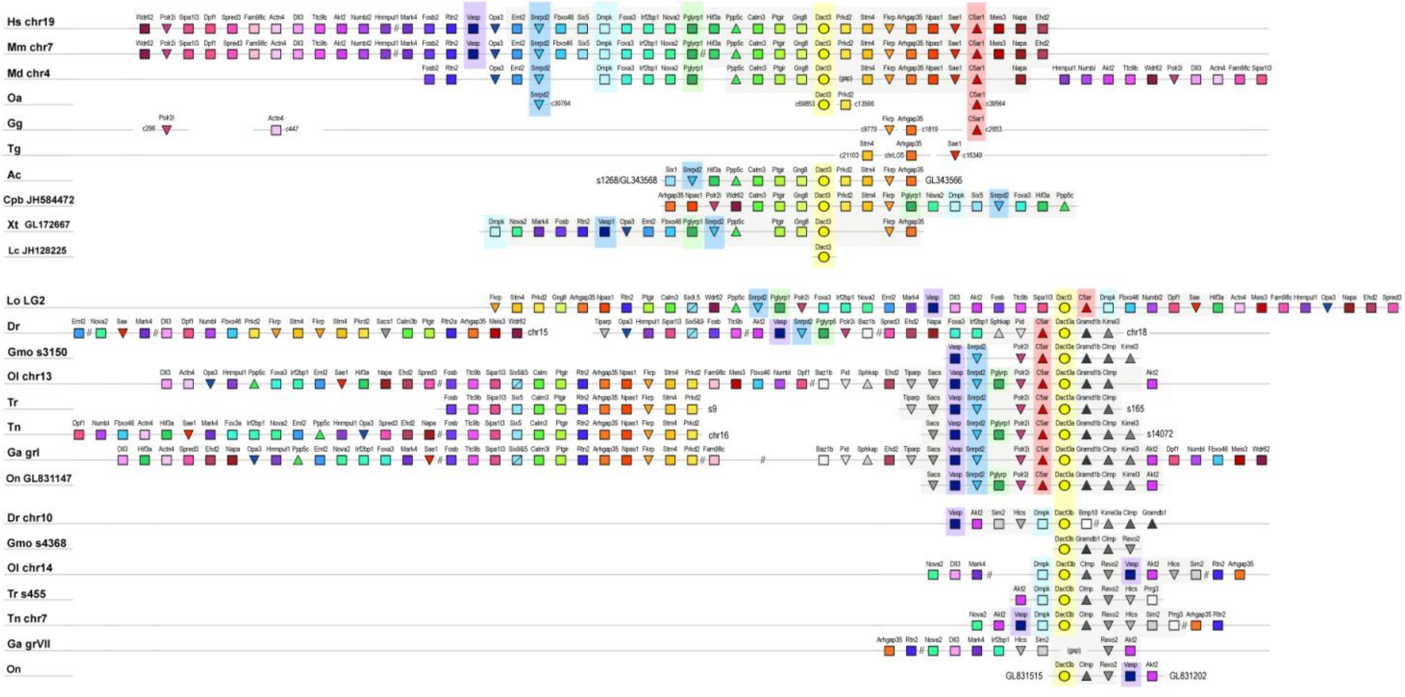

D. Dact4 loci

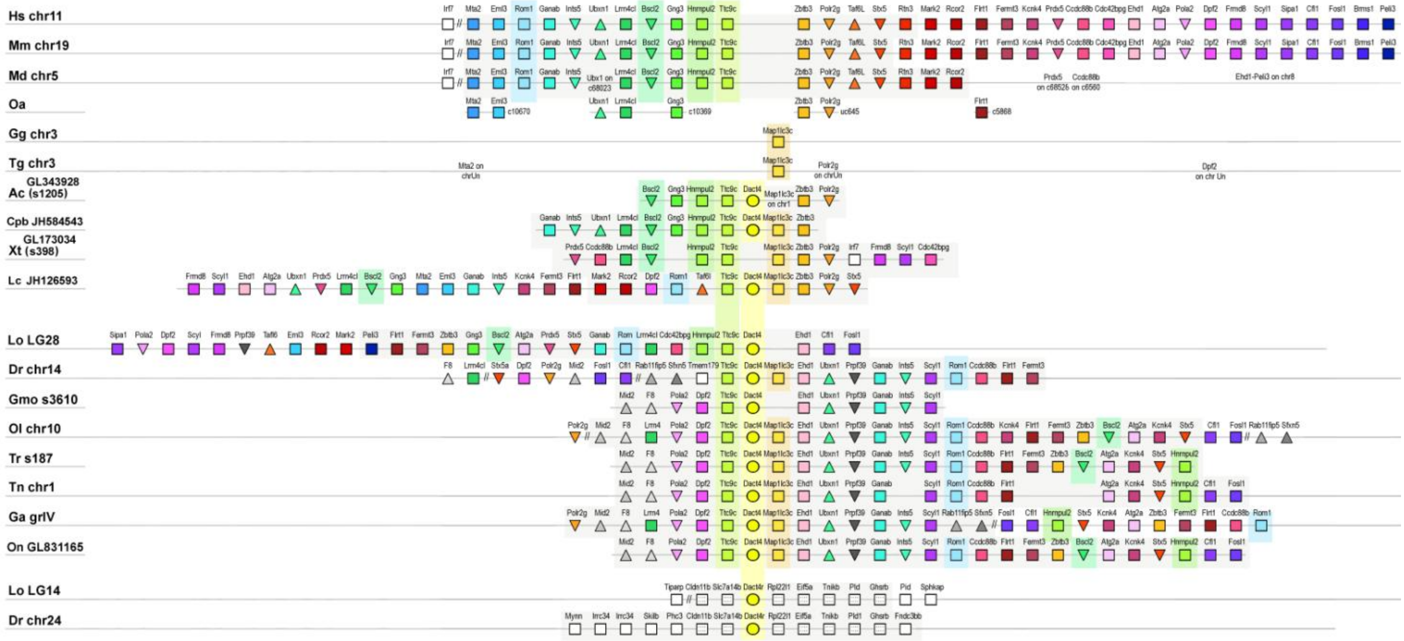
Hs chr11
Mm chr19
Md chr5
Oa
Gg chr3
Tg chr3
GL343928
Ac (s1205)
Cpb JH584543
GL173034
Xt (s398)
Lc JH126593
Lo LG28
Dr chr14
Gmo s3610
Ol chr10
Tr s187
Tn chr1
Ga grIV
On GL831165
Lo LG14
Dr chr24
