# Supplementary material for: Dact genes are chordate specific regulators at the intersection of Wnt and Tgf-β signaling pathways
Source: BMC Evol Biol. 2014 Aug 6;14:157. doi: 10.1186/1471-2148-14-157 (PMC4236578; doi:10.1186/1471-2148-14-157)
Supplement: Additional file 7 — Phylogenetic protein trees for genes associated with all four Dact loci. [file 1471-2148-14-157-S7.pdf]

Additional File 7. Phylogenetic protein trees for genes associated with all four *Dact* loci.

The color code used in the trees and abbreviations are as in Figure 2. Note that wherever statistically well supported metagroups appeared in these trees, they encompassed *Dact1,3* and/or *Dact2,4* associated genes; all other permutations (linking *Dact1,2*; *Dact1,4*; *Dact3,2* or *Dact3,4* associated genes) did not occur. This supports the idea that *Dact1* and *Dact3* loci arose from one, *Dact2* and *Dact4* loci from the second locus generated during the 1st round of vertebrate genome duplication.

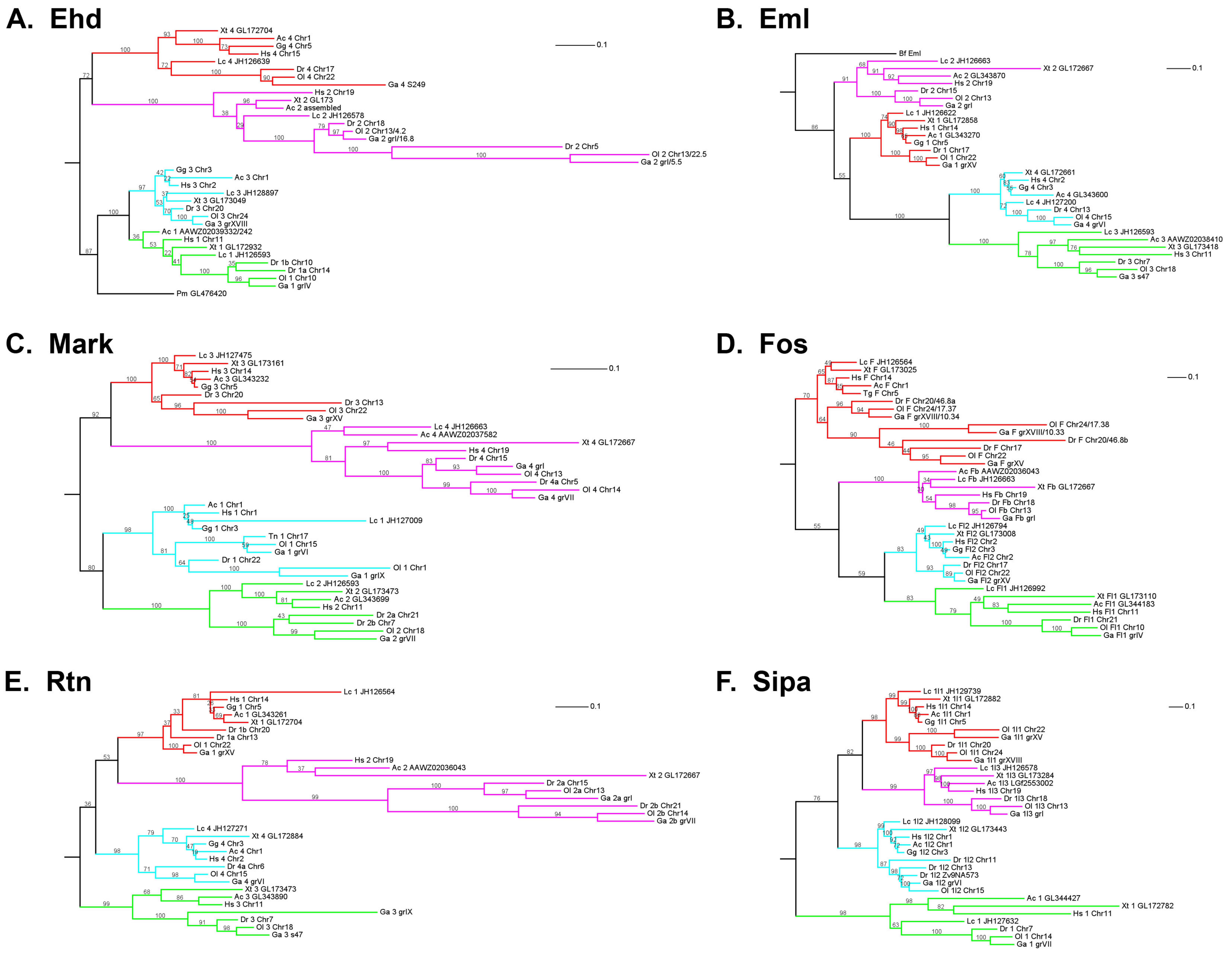
A. Ehd
0.1
Xt 4 GL172704
Ac 4 Chr1
Gg 4 Chr5
Hs 4 Chr15
Lc 4 JH126639
Dr 4 Chr17
Ol 4 Chr22
Ga 4 S249
Hs 2 Chr19
Xt 2 GL173
Ac 2 assembled
Lc 2 JH126578
Dr 2 Chr18
Ol 2 Chr13/4.2
Ga 2 grI/16.8
Dr 2 Chr5
Ol 2 Chr13/22.5
Ga 2 grI/5.5
Gg 3 Chr3
Ac 3 Chr1
Hs 3 Chr2
Lc 3 JH128897
Xt 3 GL173049
Dr 3 Chr20
Ol 3 Chr24
Ga 3 grXVIII
Ac 1 AAWZ02039332/242
Hs 1 Chr11
Xt 1 GL172932
Lc 1 JH126593
Dr 1b Chr10
Dr 1a Chr14
Ol 1 Chr10
Ga 1 grIV
Pm GL476420
B. Eml
0.1
Bf Eml
Lc 2 JH126663
Xt 2 GL172667
Ac 2 GL343870
Hs 2 Chr19
Dr 2 Chr15
Ol 2 Chr13
Ga 2 grI
Lc 1 JH126622
Xt 1 GL172858
Hs 1 Chr14
Ac 1 GL343270
Gg 1 Chr5
Dr 1 Chr17
Ol 1 Chr22
Ga 1 grXV
Xt 4 GL172661
Hs 4 Chr2
Gg 4 Chr3
Ac 4 GL343600
Lc 4 JH127200
Dr 4 Chr13
Ol 4 Chr15
Ga 4 grVI
Lc 3 JH126593
Ac 3 AAWZ02038410
Xt 3 GL173418
Hs 3 Chr11
Dr 3 Chr7
Ol 3 Chr18
Ga 3 s47
C. Mark
0.1
Lc 3 JH127475
Xt 3 GL173161
Hs 3 Chr14
Ac 3 GL343232
Gg 3 Chr5
Dr 3 Chr20
Dr 3 Chr13
Ol 3 Chr22
Ga 3 grXV
Lc 4 JH126663
Ac 4 AAWZ02037582
Xt 4 GL172667
Hs 4 Chr19
Dr 4 Chr15
Ga 4 grI
Ol 4 Chr13
Dr 4a Chr5
Ol 4 Chr14
Ga 4 grVII
Ac 1 Chr1
Hs 1 Chr1
Lc 1 JH127009
Gg 1 Chr3
Tn 1 Chr17
Ol 1 Chr15
Ga 1 grVI
Dr 1 Chr22
Ol 1 Chr1
Ga 1 grIX
Lc 2 JH126593
Xt 2 GL173473
Ac 2 GL343699
Hs 2 Chr11
Dr 2a Chr21
Dr 2b Chr7
Ol 2 Chr18
Ga 2 grVII
D. Fos
0.1
Lc F JH126564
Xt F GL173025
Hs F Chr14
Ac F Chr1
Tg F Chr5
Dr F Chr20/46.8a
Ol F Chr24/17.37
Ga F grXVIII/10.34
Ol F Chr24/17.38
Ga F grXVIII/10.33
Dr F Chr20/46.8b
Dr F Chr17
Ol F Chr22
Ga F grXV
Ac Fb AAWZ02036043
Lc Fb JH126663
Xt Fb GL172667
Hs Fb Chr19
Dr Fb Chr18
Ol Fb Chr13
Ga Fb grI
Lc Fl2 JH126794
Xt Fl2 GL173008
Hs Fl2 Chr2
Gg Fl2 Chr3
Ac Fl2 Chr2
Dr Fl2 Chr17
Ol Fl2 Chr22
Ga Fl2 grXV
Lc Fl1 JH126992
Xt Fl1 GL173110
Ac Fl1 GL344183
Hs Fl1 Chr11
Dr Fl1 Chr21
Ol Fl1 Chr10
Ga Fl1 grIV
E. Rtn
0.1
Lc 1 JH126564
Hs 1 Chr14
Gg 1 Chr5
Ac 1 GL343261
Xt 1 GL172704
Dr 1b Chr20
Dr 1a Chr13
Ol 1 Chr22
Ga 1 grXV
Hs 2 Chr19
Ac 2 AAWZ02036043
Xt 2 GL172667
Dr 2a Chr15
Ol 2a Chr13
Ga 2a grI
Dr 2b Chr21
Ol 2b Chr14
Ga 2b grVII
Lc 4 JH127271
Xt 4 GL172884
Gg 4 Chr3
Ac 4 Chr1
Hs 4 Chr2
Dr 4a Chr6
Ol 4 Chr15
Ga 4 grVI
Xt 3 GL173473
Ac 3 GL343890
Hs 3 Chr11
Ga 3 grIX
Dr 3 Chr7
Ol 3 Chr18
Ga 3 s47
F. Sipa
0.1
Lc 1l1 JH129739
Xt 1l1 GL172882
Hs 1l1 Chr14
Ac 1l1 Chr1
Gg 1l1 Chr5
Ol 1l1 Chr22
Ga 1l1 grXV
Dr 1l1 Chr20
Ol 1l1 Chr24
Ga 1l1 grXVIII
Lc 1l3 JH126578
Xt 1l3 GL173284
Ac 1l3 LGf2553002
Hs 1l3 Chr19
Dr 1l3 Chr18
Ol 1l3 Chr13
Ga 1l3 grI
Lc 1l2 JH128099
Xt 1l2 GL173443
Hs 1l2 Chr1
Ac 1l2 Chr1
Gg 1l2 Chr3
Dr 1l2 Chr11
Dr 1l2 Chr13
Dr 1l2 Zv9NA573
Ga 1l2 grVI
Ol 1l2 Chr15
Ac 1 GL344427
Xt 1 GL172782
Hs 1 Chr11
Lc 1 JH127632
Dr 1 Chr7
Ol 1 Chr14
Ga 1 grVII
