# Supplementary material for: Dact genes are chordate specific regulators at the intersection of Wnt and Tgf-β signaling pathways
Source: BMC Evol Biol. 2014 Aug 6;14:157. doi: 10.1186/1471-2148-14-157 (PMC4236578; doi:10.1186/1471-2148-14-157)
Supplement: Additional files 8 — Dact protein identity matrix. [file 1471-2148-14-157-S8.pdf]

Additional File 8. Identity of Dact proteins and most conserved regions.

Amino acid identity of Dact proteins based on (A) the full, gapped alignment, (B) the trimmed alignment used for the phylogenetic trees, (C) the alignment for the exon1-3 encoded motif 2 that encompasses the leucine zipper, a nuclear export signal and that participates in Alk5 inhibition, and (D) the C-terminal motif 11 encoded by exon 4 that encompasses a predicted nuclear localization signal, the Vangl binding domain and the PDZ binding domain. Only sequences that were complete (A, B) or for which the respective motif sequence was available (C,D) were used.

(A) whole sequence						
	**Dact1**	**Dact3**	**Dact2**	**Dact4**	**Lj/Pm**	**Bfl**
**Dact1**	56.0%	18.7%	19.5%	10.3%	15.4%	8.8%
**Dact3**	18.7%	26.3%	13.2%	7.4%	11.3%	6.7%
**Dact2**	19.5%	13.2%	40.6%	11.4%	14.0%	8.4%
**Dact4**	10.3%	7.4%	11.4%	16.2%	7.7%	5.3%
**Lj/Pm**	15.4%	11.3%	14.0%	7.7%	11.3%	6.5%
**Bfl**	8.8%	6.7%	8.4%	5.3%	6.5%	ID
**Overall protein ID: 18.5%**						

(B) whole sequence trimmed						
	**Dact1**	**Dact3**	**Dact2**	**Dact4**	**Lj/Pm**	**Bfl**
**Dact1**	81.5%	44.4%	48.1%	28.9%	42.0%	25.4%
**Dact3**	44.4%	47.8%	33.6%	19.5%	30.6%	19.5%
**Dact2**	48.1%	33.6%	66.8%	32.4%	37.5%	24.3%
**Dact4**	28.9%	19.5%	32.4%	47.4%	23.5%	17.6%
**Lj/Pm**	42.0%	30.6%	37.5%	23.5%	35.0%	18.5%
**Bfl**	24.5%	19.5%	24.3%	17.6%	18.5%	ID
**trimmed: 40.4%**						

(C) motif 2 (encoded by exons 1-3)						
	**Dact1**	**Dact3**	**Dact2**	**Dact4**	**Lj/Pm**	**Bfl**
**Dact1**	90.4%	27.2%	61.4%	29.0%	46.5%	29.8%
**Dact3***	27.2%	27.7%	27.1%	15.0%	22.3%	18.4%
**Dact2**	61.4%	27.1%	85.1%	31.4%	46.4%	27.8%
**Dact4**	29.0%	15.0%	31.4%	37.1%	27.3%	17.3%
**Lj/Pm**	46.5%	22.3%	46.4%	27.3%	37.6%	20.8%
**Bfl**	29.8%	18.4%	27.8%	17.3%	20.8%	ID

(D) motif 11 (C-terminus)						
	**Dact1**	**Dact3**	**Dact2**	**Dact4**	**Lj/Pm**	**Bfl**
**Dact1**	81.8%	43.6%	42.5%	12.9%	27.4%	15.4%
**Dact3**	43.6%	54.7%	31.8%	11.3%	22.4%	13.3%
**Dact2**	42.5%	31.8%	61.2%	15.3%	23.8%	12.5%
**Dact4**	12.3%	11.3%	15.3%	33.0%	9.1%	8.3%
**Lj/Pm**	27.4%	22.4%	23.8%	9.1%	19.2%	8.6%
**Bfl**	15.4%	13.3%	12.5%	8.3%	8.6%	ID

* strong reduction of motif 2 in teleost dact3b

ID = identical
