# Supplementary material for: Dact genes are chordate specific regulators at the intersection of Wnt and Tgf-β signaling pathways
Source: BMC Evol Biol. 2014 Aug 6;14:157. doi: 10.1186/1471-2148-14-157 (PMC4236578; doi:10.1186/1471-2148-14-157)
Supplement: Additional file 9 — Conserved Dact protein motifs. [file 1471-2148-14-157-S9.pdf]

Additional File 9. Sequences of Dact protein motifs.

Core amino acids (aa) of a motif are underlined, motif variations typical for a particular Dact ortholog are highlighted in yellow, variations typical for Dact1,3 proteins in red and variations typical for Dact2,4 proteins in green. Leucines contributing to a L-zipper are highlighted in blue. The regions containing the linked motifs 2a-f and the linked motifs 11a-g are the most conserved regions in Dact proteins.

MOTIF 1
Position: exon 1-encoded
Size: 24-36aa
Known function: (region involved in Alk5 blocking)
Predicted function: nuclear export signal

Dact1: ER-Q/V-RTRER-L/Q-EATLAGL-A/G/T-EL-E/D/G-Y/F-LR-Q/H/R-RQE, **followed by conserved** LLV
Dact3: ER-S/G-R-L/N/T **followed by** K/R-G/S-WLEGSLAGLCEL-H/P/Q-W/L-LRERQE **(amniotes) or** K/R-ERLEASLAGLCELEL-L/R-K/R-Q/E-RQE (**anamniotes)**
Dact2: DR-G/S/C/R-RVGERLQAALAGLQEL-(xx)-LR-E/D-K/R-Q
Dact4: SLWSG-T/S-E/D-RVRIGERL-K/Q-A-T/S-LAG-V/I/L-LELE-L/V-LR-(x)-K/R-H/Q-L-E/D-MV-D/E **Dact4**
SLWSG-T/S - GRKRIRDRFCATVAGLLELEVLRVKHKVMVE **dact4r**
***Lj* dactA:** APRLTAIRLESALAGIRHLESLKRRQG
***Lj* dactB:** ERSRASERLEASMAGLAELELLRRRHE
***Lj* dactCorD?:** ERIRARERLEASIAALRELQLLREERE
***Bfl* dact**: DRSLQ-(gap)-ARLMASEAAMEELRLLRQRQE

MOTIF 2a
Position: encoded by 3‘ end of exon 1
Size: 5-15aa
Known function: (region involved in Alk5 blocking)
Predicted function: ---

Dact1: **conserved** EEK **followed by** F/L-LEENILLLRKQL
Dact3: L/P-EEQL **amniotes**
L-R/K-R/K-QL **Latimeria, gar, teleost dact3a**
Dact2: **conserved** E-Q/H **followed by** RLEATL-T/A/S-ALK-E/Q-QL
Dact4: R/G/Q/P-QQV **(not conserved in dact4r)**
***Lj* dactA:** LGGRD-WLYALLV-GPHL
***Lj* dactB:** LE-RDFVESLRAM-KEQL
***Lj* dactCorD:** DDALLAVGRDLELI-KEQL
***Bfl* dact**: CINSIDKLRNAV

MOTIF 2b
Position: encoded by 5’ end of exon 2
Size: 10-16aa
Known function: (region involved in Alk5 blocking)

Predicted function: ---
Dact1: NCLRRRDAGL
Dact3: absent
Dact2: SRLRRQDVGL, linked to motif 2c by K-S/T-HL-D/Q-Q
Dact4: absent
***Lj* dactA:** absent
***Lj* dactB:** GCLPQRDLGLL
***Lj* dactC:** absent
LJ* dactD*: no sequence available
Pm* dact*: no sequence available
Bfl* dact*: DHLKRTDSDL

MOTIF 2c
Position: encoded by centre of exon 2
Size: 11-13aa
Known function: nuclear export signal; region also involved in Alk5 blocking
Predicted function: ---
Dact1: ELD-K/R-QISDLRLDV
Dact3: L-E/G/Q-QQ-V/L-GEL-R/S/K-L/V/I-D/E
Dact2: QLD-Q/R-Q/R-ISELKLDV
Dact4: absent
Lj* dactA*: LLEQRFCELGLRG
Lj* dactB*: HLNEQFSELKMDM
Lj* dactC*: ALERPLSELRVDT
LJ* dactD*: no sequence available
Pm* dact*: no sequence available
Bfl* dact*: LNTQILQLKLA

MOTIF 2d
Position: encoded by the 3' end of exon 2
Size: 6-9aa
Known function: (region involved in Alk5 blocking)
Predicted function: ---
Dact1: ETDSRPSS
Dact3: EQESGRSS - **placental mammals**
ETDSWPSS - **reptiles**
DSRPSS - **gar, teleost dact3a; no conserved end of exon 2 in teleost dact3b**
Dact2: L-E/D-SDSRPSS
Dact4: E/D-SDSRPSS
Lj* dactA*: ESDSRASS
Lj* dactB*: ETDSRPSS
Lj* dactC*: DVDSRPSS
LJ* dactD*: no sequence available
Pm* dact*: no sequence available
Bfl* dact*: DSRRSS

MOTIF 2e
Position: encoded by 5' end of exon 3

Size: 4-11aa
Known function: (region involved in Alk5 blocking)
Predicted function: ---
Dact1: GFY-E/D-LSDG-A/T-SG
Dact3: GFYE, **followed by D-**P/S-S-S/P **in amniotes**
or by SSEG-Q/P/L-SP **in teleost dact3a;**
not conserved in *Xenopus dact3* and zebrafish dact3b,
whole exon lacking in acanthopterygian teleost dact3b
Dact2: GFY-E/D-LSDGGSC
Dact4: GFY-E/D-T/V-S-E/D-M/S-G **in sarcopterygians**
GFYSVSGS **in actinopterygian dact4**
GFC-E/D **in gar and zebrafish dact4r**
***Pm/Lj* dactA:** GFYELSDAGTG
***Pm/Lj* dactB:** GYYDLSDG
***Lj* dactC:** GFYSMSEDGSL
LJ* dactD*: no sequence available
Bfl* dact*: GFFDASSP

MOTIF 2f

Position: exon 3-encoded, in the protein sequence closely linked to motif 2e
Size: 14-17aa
Known function: (region involved in Alk5 blocking)
Predicted function: ---
Dact1: SLSNSSNSVFSECLSSC
Dact3: absent **(note: whole exon lacking in acanthopterygian teleost dact3b)**
Dact2: SLSNSCTSVYSE-S/C-L/I/M-SSS
Dact4: SLS-D/N-S-C/S-(x)-S-V/M-S/H/C-S-E/D-(xx)-PGG
***Pm/Lj* dactA:** SLSTSCNSVFSDLVPSP
***Pm/Lj* dactB:** SLSNSCNSIF-N/S-DCLS-D/G-S
***Lj* dactC:** SLSNSCSSISSSGPAP
Pm/LJ* dactD*: no sequence available
***Bfl* dact:** SPPLTPDIASPEKLHHY

MOTIF 3a

Position: encoded by the 3' end of exon 3
Size: 7-9aa
Known function: (region involved in Alk5 blocking)
Predicted function: ---
Dact1: absent
Dact3: absent **(note: whole exon lacking in acanthopterygian teleost dact3b**)
Dact2: DY **(sarcopterygians, Cm), followed by** R-P/R-R/K-SA **and a conserved** DE
Dact4: RP-R/H-ST**, followed by** DE **in sarcopterygians**
RP-R/H-S-L/A-DH **gar, cyprinid teleost dact4**
PPKLWEQ **other teleosts**
Pm/Lj* dactA*: absent
***Pm/Lj* dactB:** absent
***Lj* dactC:** RPRSAVE
Pm/LJ* dactD*: no sequence available
Bfl* dact*: absent

MOTIF 3b
Position: encoded by the 3' end of exon 3
Size: 6-7aa
Known function: (region involved in Alk5 blocking)
Predicted function: ---
Dact1: D/E-G-R/C/Q-P/L/A-KSA
Dact3: S/N/C-ERPKS-V/L/A
whole exon lacking in acanthopterygian teleost dact3b
Dact2: RPRPVST
Dact4: RRP-V/F-ST **sarcopterygians, gar, cyprinid teleost Dact4**
***Pm/Lj* dactA:** ERRPLST
***Lj* dactB:** LERPLSS
***Lj* dactC:** PSRPQSS
***Pm/LJ* dactD:** no sequence available
***Bfl* dact:** absent

MOTIF 3c
Position: encoded by 5' end of exon 4
Size: 3-4aa
Known function: (region implicated in Alk5 blocking and Tcf3 binding)
Predicted function: ---
Dact1: DLI
Dact3: G-D-A/P/V
Dact2: G-DL **followed by conserved** E/D-R
Dact4: G-DLE
***Pm/Lj* dactA:** G-DLK
***Pm/Lj* dactB:** G-DLL
***Lj* dactC:** DLG
***Pm/Lj* dactD:** absent
***Bfl* dact:** GESD

MOTIF 4a
Position: exon 4-encoded, motif approximately 25 aa downstream of motif 3c
Size: 10-11aa
Known function: (region implicated in Alk5 blocking and Tcf3 binding)
Predicted function: ---
Dact1: VCRS-L/F/P-S-T/A-P-H/Q-S/F-N **- most tetrapods**
VRRSFSAPYS **- gar, teleosts**
Dact3: V/L-PRSFSAPYP - YP **not conserved in teleost dact3b**
Dact2: absent
Dact4: absent
***Pm/Lj* dactA:** VRRSASEHGS
***Pm/Lj* dactB:** TPGPPPPA-G/A-CP
***Lj* dactC:** LADPVTSAPGR
***Pm/Lj* dactD:** absent
***Bfl* dact:** absent

MOTIF 4b

Position: exon 4-encoded, motif approximately 15 aa downstream of motif 4a
Size: 12-35aa
Known function: (region implicated in Alk5 blocking and Tcf3 binding**)**
Predicted function: ---
Dact1: DV-H/N/Q **followed by** PKYQCDLVSKNGND-V/I-YRYPSPLHAVAVQSPMF
Dact3: FLYPSPLHAVA-L/M**– conserved in sarcopterygians only**
KKHATLA **weak similarity in teleost dact3b**
Dact2: D **followed by** PK-Y/F-Q-S/N/R/C-D/N-LVS-K/R-N/S/G-G-(x)-D/E-VY-P/H/L/R-YPSPLHAVALQSPLFS
Dact4: LD **followed by** P-R/K-Y-C/K/R-T/S/C-DLVSR-R/N/K/S-T-K/S-EVY-H/S/P/R-YPSPLHAVALQSPLF
Pm/Lj* dactA*: LSPP-W/R-LTVLDS-A/V-E-S/P-V/G-APSA-N/H-PAMTPDTRS
Pm/Lj* dactB*: EASGRYVSDLTSRDGSDVYRYPSPLHAVAIQSPLF
Lj* dactC*: DPRFRCDLRSRDGSEVYRYPSPLHAIAMQSSAF
Pm/Lj* dactD*: TRRFARDVPSLDGSGLYRYPSALHAVAIQSPAL
Bfl* dact*: FKLALRPRSKTGLSVCYPYPSPMHAIAIQSDM

MOTIF 5a
Position: exon 4-encoded, approximately 80-110 aa downstream of motif 4b
Size: 9-14aa
Known function: region implicated in Tcf3 binding
Predicted function: ---
Dact1: KK **followed by** D-G/S-YILSL-V/L/I-Q-K/R-K/R
Dact3: R/H-R/K-V/L/A-E-G/T/S/N-YI-L/S/F-G/S/A/R-L-I/L-Q/R-RR
Dact2: GYI-N/D-K/R-LL-Q/R-L/R
Dact4: L-D/E-Q/R-YI-S/A-R/K-L-(xx) R/Q **(not dact4r)**
Pm/Lj* dactA*: A/V-R-S/G-DAALPI-A/T-KQQ
Pm/Lj* dactB*: ERVSGYICSLLDRK
Lj* dactC*: DKVTAYIAALLKRR
Pm/Lj* dactD*: GKPDDYIARLLERR
Bfl* dact*: SAGLLRPV

MOTIF 5b
Position: exon 4-encoded, linked to motif 5a
Size: 9-10aa
Known function: (region implicated in Tcf3 and Dvl binding)
Predicted function: ---
Dact1: conserved V **followed by** RTNKPRTS-L/V/I
Dact3: RP-S/C/G-K/Q-PRT-S/T-L
Dact2: absent
Dact4: absent
Pm/Lj* dactA*: absent
***Pm/Lj* dactB:** NGRLKPKHEE
***Lj* dactC:** PALVRPASIA
Pm* dactD*: CRLSRPRRTP (***Lj*D:** VRLSLRSPPLP)
Bfl* dact*: KAPRKPKPTP

MOTIF 5c

Position: exon 4-encoded, linked to motif 5c
Size: 6-12aa
Known function: (region implicated in Dvl binding)
Predicted function: ---
Dact1: K-G/S-I/L-L **followed by** R-H/Q/N-GS-M/V/L/I-CVR
Dact3: RRQ-S/N-S-L/V-C/R/H **(less conserved but recognisable in teleost dact3a)**
Dact2: GGV **followed by** KINSSSSQL **and** EK **in birds and reptiles; motif recognizable in mammals**
Dact4: K/R-SH-(x)-S-(x)-Q/S-S-L/V-S/C
Pm/Lj* dactA*: absent
Pm/Lj* dactB*: KPDTLQ-C/G-VDSCKE
***Lj* dactC:** absent
Lj* dactD*: absent ***(Pm*D: no sequence available for motifs 6-11g)**
Bfl* dact*: RQSLLK

MOTIF 6
Position: exon 4-encoded
Size: 9aa
Known function: ---
Predicted function: ---
Dact1: absent
Dact3: absent
Dact2: absent
Dact4: R/K-I/S/T-R-R/P-RISTC **(no sequence available for dact4r)**
***Pm/Lj* dactA:** absent
***Pm/Lj* dactB:** absent
***Lj* dactC:** absent
***Lj* dactD:** absent
Bfl* dact*: absent

MOTIF 7a
Position: exon 4-encoded, approximately 250aa downstream of motif 3c and 100-120aa downstream of motif 5
Size: 21aa **Known function**: ---
Predicted function: ---
Dact1: EERP-A/P/I-L-D/E-F/L-KS-E/L-GSSSQSL-D/E-E/D-G
Dact3: absent
Dact2: absent
Dact4: absent **(no sequence available for dact4r)**
***Pm/Lj* dactA:** absent
***Pm/Lj* dactB:** absent
***Lj* dactC:** absent
***Lj* dactD:** absent
Bfl*dact*: absent

MOTIF 7b
Position: exon 4-encoded, linked to motif 7a
Size: 10-14aa
Known function: ---
Predicted function: ---

Dact1: LVN **followed by** A-Q/H-Y/F-IPAQQQ
Dact3: L/M-V-S/K/N **followed by** AQYIP-G/A-Q/A **-most species**
LV-S/N-A-Q/K/E/S-YIP-G/A-Q **-teleosts**
Dact2: EFVH **followed by** A-Q/K-FVPA-E/G-S-H/Q-Q/R
Dact4: absent **(no sequence available for dact4r)**
***Pm/Lj* dactA:** VMMAIPEEQQ
***Pm/Lj* dactB:** TAIKSSKK
***Lj* dactC:** absent
***Lj* dactD:** absent
Bfl* dact*: absent

MOTIF 7c
Position: exon 4-encoded, linked to motif 7b
Size: 14-16aa
Known function: ---
Predicted function: ---
Dact1: K/R-NV **followed by** KI-L/V-KVKSS-T/A/S-LKHR
Dact3: absent
Dact2: KT **followed by** KAVK-L/I/V-KRR-N/S-SEK
Dact4: absent **(no sequence available for dact4r)**
***Pm/Lj* dactA:** KIVKFAKTSRCREPSG
***Pm/Lj* dactB:** KRLGPTKARPPKVEE
***Lj* dactC:** absent
***Lj* dactD:** absent
Bfl* dact*: absent

MOTIF 8a
Position: exon 4-encoded
Size: 4-5aa
Known function: nuclear localization
Predicted function: ---
Dact1: REK-(x)-K/R
Dact3: RE-K/R-P-R/K **(in sarcopterygians except *Xenopus*)**
Dact2: E-K/R-(x)-R
Dact4: absent **(no sequence available for dact4r)**
***Pm/Lj* dactA:** (KGGRQ)
***Pm/Lj* dactB:** absent
***Lj* dactC:** absent
***Lj* dactD:** absent
Bfl* dact*: absent

MOTIF 8b
Position: exon 4-encoded, linked to motif 8a
Size: 6-8aa
Known function: nuclear localization
Predicted function: ---
Dact1: KKCRFP-D/E-D/E
Dact3: K/R-K/R-C/G/N-R/H-(xx)-E-D/R/E **(in non-mammalian sarcopterygians and teleost dact3b)**

Dact2: absent
Dact4: absent **(no sequence available for dact4r)**
***Pm/Lj* dactA:** KRCRFSED
***Pm/Lj* dactB:** Q/P-RARRRDE
***Lj* dactC:** RICKLGHG
***Lj* dactD:** RRCKSASL
Bfl* dact*: RRCNEN

MOTIF 8c
Position: exon 4-encoded, linked to motif 8b
Size: 3-4aa
Known function: nuclear localization
Predicted function: ---
Dact1: KK-(xx)-KK
Dact3: R/K-R/K
Dact2: RRP
Dact4: absent **(no sequence available for dact4r)**
***Pm/Lj* dactA:** absent
***Pm/Lj* dactB:** absent
***Lj* dactC:** absent
***Lj* dactD:** absent
Bfl* dact*: absent

MOTIF 9
Position: exon 4-encoded
Size: 5-8aa
Known function: ---
Predicted function: ---
Dact1: SESSL **(only in cartilaginous fish)**
Dact3: RSQSENSL
Dact2: RSCSESSL
Dact4: absent **(no sequence available for dact4r)**
***Pm/Lj* dactA:** RSQSDNSI
***Lj* dactB:** absent ***(Pm*B: no sequence available for motifs 9-11d)**
***Lj* dactC:** absent
***Lj* dactD:** absent
***Bfl* dact:** absent

MOTIF 10
Position: exon 4-encoded
Size: 7-13aa
Known function: ---
Predicted function: ---
Dact1: HGHGRE-V/A/Q/P-V-V/L-A
Dact3: V/A/L-PE-(xxx)-R/P-K/R-Y-(x)-T-(x)-ER-D/E
Dact2: no conservation in this region
Dact4: LY-R/K-GKHAS-H/R-E/K-LV-R/K **(no sequence available for dact4r)**
***Pm/Lj* dactA:** absent
***Lj* dactB:** absent

Lj **dactC:** EREETAG
Lj **dactD:** ERGKMAAAAAECAD
Bfl **dact:** absent

MOTIF 11a
Position: exon 4-encoded, start of conserved region approximately 200 aa from C-terminus
Size: 4-9aa
Known function: ---
Predicted function: nuclear localization signal
Dact1: KPKHKR-A/T/G/N-DY
Dact3: R-P/Q-RR **(amniotes, Lc, gar)**
R/K-(x)-R/K-R/K **(teleost dact3b)**
Dact2: KKKQ
Dact4: LRKKP **followed by** WKKP **(Latimeria; 1st stretch maintained in sarcopterygians, 2nd stretch in teleosts)**
Pm/Lj **dactA:** RQRRLSSH
Lj **dactB:** GVKRLAQQ
Lj **dactC:** YRRRLVLH
Pm/Lj **dactD:** absent
Bfl **dact:** absent

MOTIF 11b
Position: exon 4-encoded, linked to motif 11a
Size: 9-15aa
Known function: ---
Predicted function: ---
Dact1: RRWKSSAEISYEEA
Dact3: RRW-R/C-S-T/N-L/A-E-L/I-SQDE-G/A-E
Dact2: RKWQSTVEIS
Dact4: KW-T/A/V/M-SVLEIS **(in sarcopterygians and *Cm*; no sequence for the gar)**
Pm/Lj **dactA:** RRWRSTVEICQGAA
Lj **dactB:** SRELVQSHG
Lj **dactC:** RRWCSTMEIARGGG
Lj **dactD:** RRLCRAGDAGGDWA
Bfl **dact:** absent

MOTIF 11c
Position: exon 4-encoded, linked to motif 11b
Size: 5-10aa
Known function: ---
Predicted function: ---
Dact1: RRARR-R/N-Q/A/N-RRE
Dact3: RR-(xx)-RR-(x)-R
Dact2: PR-Q/P-P/A-A/G-RRAG **(sarcopterygians and gar)**
Dact4: RRALFRS **(only teleosts)**
Pm/Lj **dactA:** RRASRR-D/E-A
Lj **dactB:** RRRPAPARVR
Lj **dactC:** MRAGARDG

***Lj* dactD:** DRCYPSAAG
***Bfl* dact:** absent

MOTIF 11d
Position: exon 4-encoded
Size: 32-40aa
Known function: (region involved in Vangl binding)
Predicted function: ---
Dact1: highly conserved stretch of 32aa, starts with SDSEYSAEC and ends with TTNC
Dact3: present in *Latimeria*, but only parts in the other species
Dact2: highly conserved stretch of 30aa, starts with R/K-SESD-(x)-SEYSAEC and ends with T-T/A-N-R/C
Dact4: absent
***Pm/Lj* dactA:** present from ESDSEYSAEC to DDD, interrupted by intron
***Lj* dactB:** present from DSERSEYSAEC to TTNR
***Lj* dactC:** only parts present: SAIAEM and SED
***Lj* dactD:** absent
***Bfl* dact:** absent

MOTIF 11e
Position: exon 4-encoded
Size: 26-28aa
Known function: (region involved in Vangl binding)
Predicted function: ---
Dact1: highly conserved S-rich stretch of 28aa, starts with FGDSESS and ends with ESGG
Dact3: highly conserved S-rich stretch of 26aa, starts with E/D-SESS and ends with ESGG
Dact2: conserved S-rich stretch, starts with FGDSESS and ends with a block of acidic aa
Dact4: S-rich stretch, ill-conserved between sarcopterygians and actinopterygians
***Pm/Lj* dactA:** acidic aa alignable, but no S-rich stretch
***Pm/Lj* dactB:** acidic aa alignable, but few S residues
***Lj* dactC:** some similarity with dactA
***Lj* dactD:** absent
***Bfl* dact:** array of S,G and acidic aa that resembles sequences from gnathostome Dacts

MOTIF 11f
Position: exon 4-encoded
Size (aa): 3-9
Known function: involved in Vangl binding
Predicted function: ---
Dact1: L-I/N/V-W-S/P-QF
Dact3: LVWPQQLPP
Dact2: L-V/I-W
Dact4: absent
***Pm/Lj* dactA:** L-A/T-GS-L/T-A
***Pm/Lj* dactB:** LAGRRG
***Lj* dactC:** absent
***Lj* dactD:** absent
***Pm* dactB:** PVV
***Bfl* dact:** LVL

MOTIF 11g
Position: exon 4-encoded, C-terminus
Size: 14-33aa
Known function: PDZ binding domain; binds Dvl in Dacts 1-3
Predicted function: ---
Dact1: K-A/T-F-V **followed by** KIKASHNLKKKILRFRSGSLKLMTTV
Dact3: K-A/V-F-V **followed by** KIKASHALKKKI-L/M-RFR-S/T-GSLKVMTTV
Dact2: P-(x)-LPP-V/E-P-(xx)-C **followed by** RIKASKALKKKIRRFQPASLKVMT-M/L-V
Dact4: L-H/R/Y-R-S/T-RSFKELKK-R/M/V-V **(plus 11-16 additional aa in sarcopterygians) or** L-Q/T-RSRSLRDLGRKVFGSMRSLSLKRKPSKK **(most teleosts; dact4r- no sequence available)**
***Lj* dactA:** CRIKASHALKKKILRCRSGSLKLMTTV
***Pm/Lj* dactB:** CRIKASRSLKKKMMHFRSGSLKVITTV
***Lj* dactC:** CRVS(QQVRA)VPSAVKRKIRQRIADAAKTSTDV
***Lj* dactD:** PAARRRALLRCHSVEPALTTTV
***Bfl* dact:** ATRRRLSSQDSLSRMFPSCHLVTEL
