# Supplementary material for: Dact genes are chordate specific regulators at the intersection of Wnt and Tgf-β signaling pathways
Source: BMC Evol Biol. 2014 Aug 6;14:157. doi: 10.1186/1471-2148-14-157 (PMC4236578; doi:10.1186/1471-2148-14-157)
Supplement: Additional file 10 — Alignments of gnathostome Dact proteins. [file 1471-2148-14-157-S10.pdf]

Additional File 10. Alignments of gnathostome Dact proteins.

Gapped alignment of Dact protein sequences, protein motifs are annotated. Additional gaps have been introduced to facilitate the identification of exon boundaries.

A. Dact1.

B. Dact2.

C. Dact3.

D. Dact4.

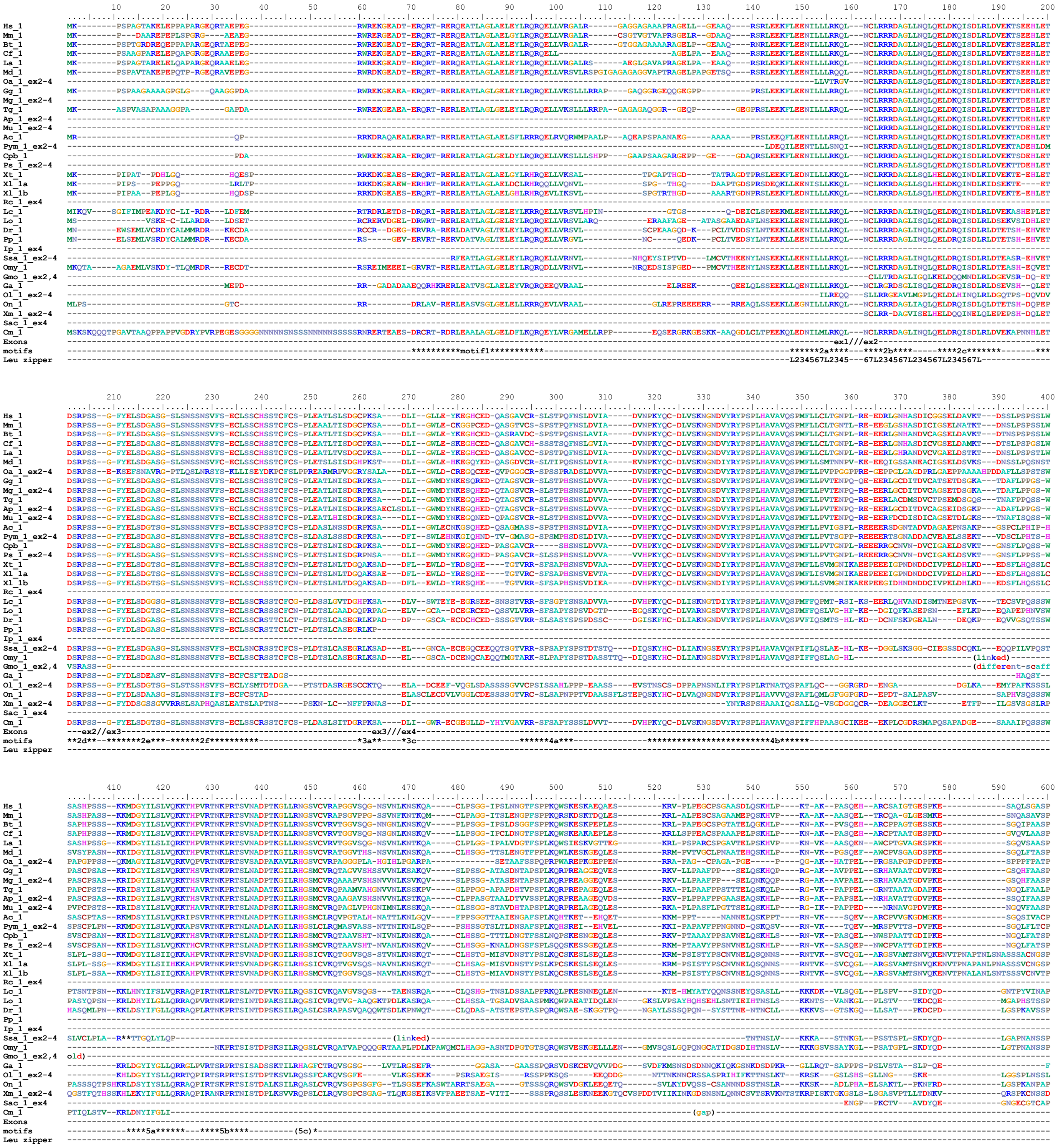
Hs_1
Mm_1
Bt_1
Cf_1
La_1
Md_1
Oa_1_ex2-4
Gg_1
Mg_1_ex2-4
Tg_1
Ap_1_ex2-4
Mu_1_ex2-4
Ac_1
Pym_1_ex2-4
Cpb_1
Ps_1_ex2-4
Xt_1
Xl_1a
Xl_1b
Rc_1_ex4
Lc_1
Lo_1
Dr_1
Pp_1
Ip_1_ex4
Ssa_1_ex2-4
Omy_1
Gmo_1_ex2,4
Ga_1
Ol_1_ex2-4
On_1
Xm_1_ex2-4
Sac_1_ex4
Cm_1
Exons
motifs
Leu zipper

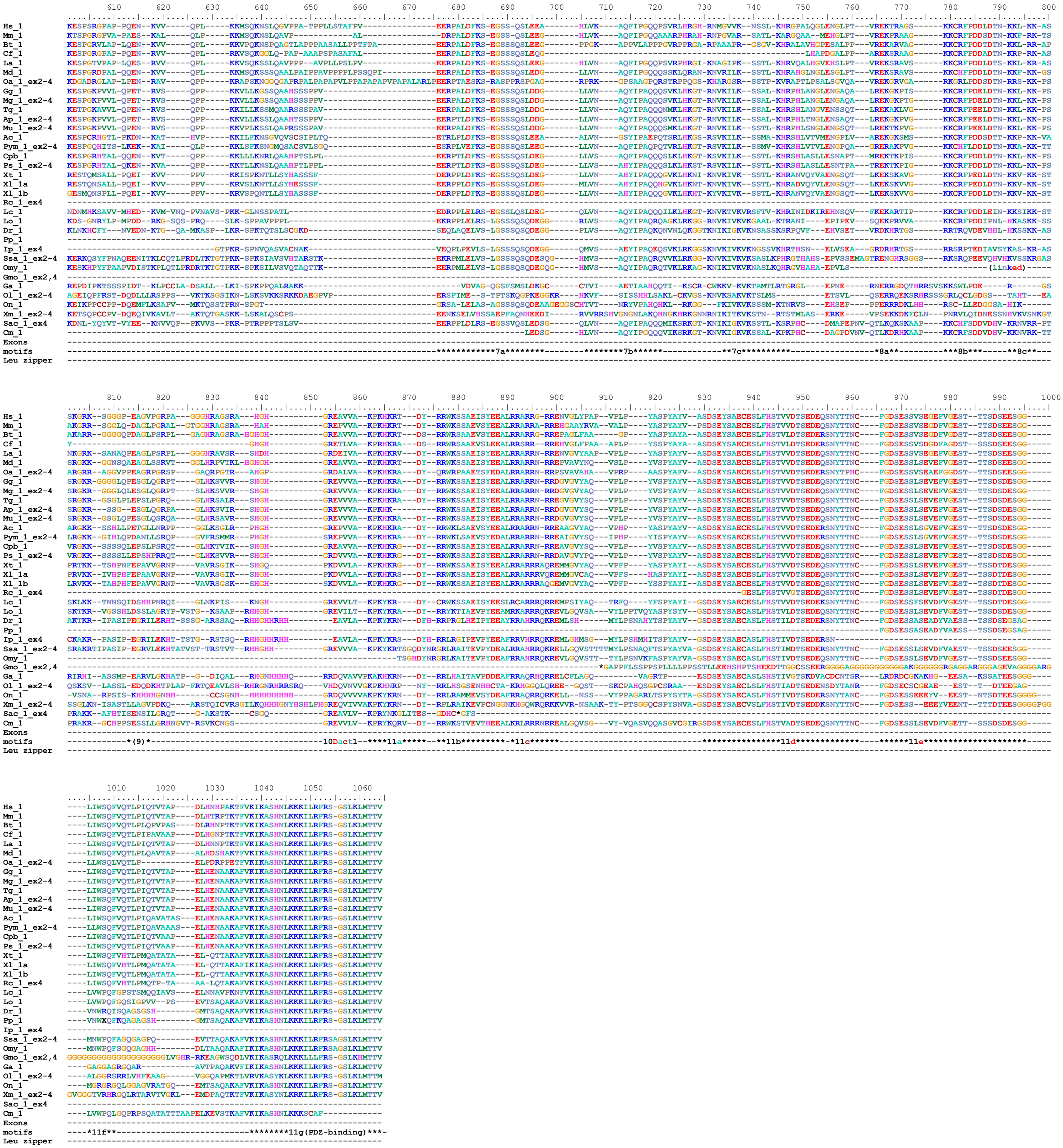
Hs_1
Mm_1
Bt_1
Cf_1
La_1
Md_1
Oa_1_ex2-4
Gg_1
Mg_1_ex2-4
Tg_1
Ap_1_ex2-4
Mu_1_ex2-4
Ac_1
Pym_1_ex2-4
Cpb_1
Ps_1_ex2-4
Xt_1
Xl_1a
Xl_1b
Rc_1_ex4
Lc_1
Lo_1
Dr_1
Pp_1
Ip_1_ex4
Ssa_1_ex2-4
Omy_1
Gmo_1_ex2,4
Ga_1
Ol_1_ex2-4
On_1
Xm_1_ex2-4
Sac_1_ex4
Cm_1
Exons
motifs
Leu zipper

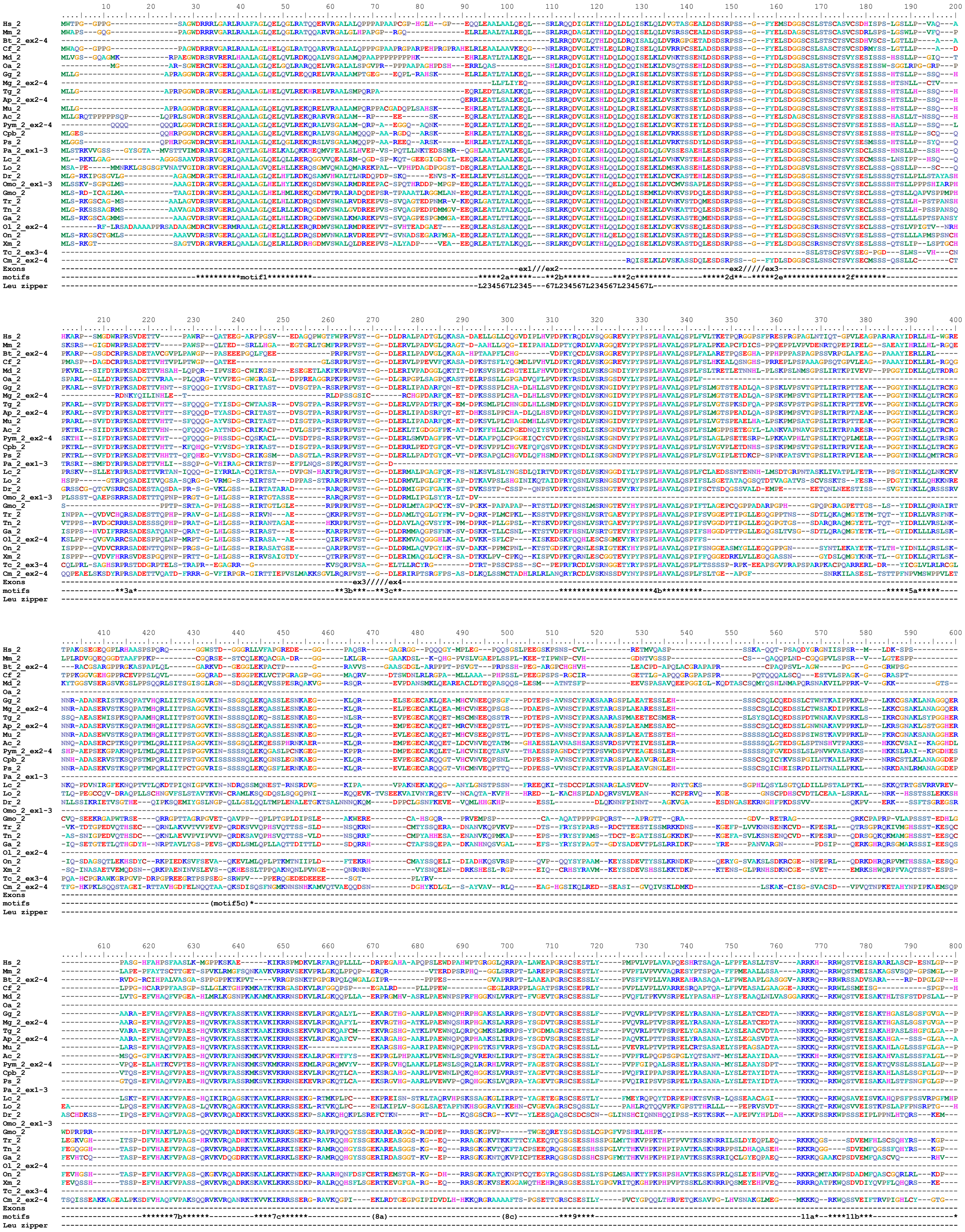
Hs_2
Mm_2
Bt_2_ex2-4
Cf_2
Md_2
Oa_2
Gg_2
Mg_2_ex2-4
Tg_2
Ap_2_ex2-4
Mu_2
Ac_2
Pym_2_ex2-4
Cpb_2
Ps_2
Pa_2_ex1-3
Lc_2
Lo_2
Dr_2
Omo_2_ex1-3
Gmo_2
Tr_2
Tn_2
Ga_2
Ol_2_ex2-4
On_2
Xm_2
Tc_2_ex3-4
Cm_2_ex2-4
Exons
motifs
Leu zipper

```
             810       820       830       840       850       860       870       880       890       900       910       920       930       940       950       960       970       980       990       1000
             ....|....|....|....|....|....|....|....|....|....|....|....|....|....|....|....|....|....|....|....|....|....|....|....|....|....|....|....|....|....|....|....|....|....|....|....|....|....|....|....|
Hs_2         RPVARRAGGP---LAR-----------------------------GRPSLV----RQDAYTRSDSEPSKHSAECDPRFPSVIPETSEGE-SSDHTTNRFGDRESSSSDEEGGAQSRDCDLALGYVAA-----GHAE--LAW----TQE-------APVS---SGPLLSPVPKLCRIKASKALKKKIRRFQPTALKVMTMV
Mm_2         RSPAKRGSGPR---AQ-----------------------------SRPTLA----RQDACARCESDPPEHSADCASLYHSTIAETSEDEEASDHTANRFGD-ESSSNDSEGCFRGGRRRLAIGSAEA-----GQGG--WAWPRVPPQQP--SR--APGN---TRPPLPPVPKLCRIKASKALKKKIRRFQPAALKVMTMV
Bt_2_ex2-4   RPAARRGGGPRPVCAR-----------------------------PRPLPA----LQDARARSASDGSERSAECASLLLSTAAESSGDE-ASDHTTSRFGDAESSGSDSGGGRRLGQ-----------------------------------R--ARSS---SRLALPPVPRLCRVKASKALKRKIRRFQPAALKVMTMV
Cf_2         RPAAGRAGGPRPVCAR-----------------------------ARPKLP----RQDARVRSESDGSEPSAEGASLPRSTVAETSEDG-ASDHTASRFGDRESSSSDSEGG---------------------------AWPGACPPQP--WR--APGA---RRPPLPPVPKLCRIKASKALRRKIRRFQPAALRVMTMV
Md_2         RPPGRRAGILRTVSSR-----------------------------SRSKIP----SHNTYAKSESDHSEYSAECASLFHSTIVETSEDD-VSDYTANCFGDSESGDSDSDGACRSSESSLTLDYEEA-----VGSE--LIWPKAIVQQP--VKGQ--AA---SKPSHHPVPKICRIKASKALKKKIRRFQPASLKVMTMV
Oa_2         ---------------------------------------------------------------------YSAECASLFHSTLAETSEEEDASDCTANRFGDSESSDGD---AARLSRSSPALHARG------TGGE--PAWP---------------RS---RMPAPPG-PRVCRIKASKALKKKIRRFQPAALKVMTTV
Gg_2         RQPARRAGVLRTVSMR-----------------------------ARPKSH----HHGAYAKSESDHSEYSAECASLFHSTIAETSEGE-VSDFTANRFGDSESSESDSDD--SSNSSSLALDYDEG-----DESE--LIWPEGSVRQL----GTAQAS---SKP-LPPVPKICRIKASKALKKKIRRFQPASLKVMTMV
Mg_2_ex2-4   RQPARRAGVLRTVSMR-----------------------------ARPKSH----HHGAYAKSESDHSEYSAECASLFHSTIAETSEGE-VSDFTANRFGDSESSESDSDD--SSNSSSLALDYDEG-----DESE--LIWPEGSVRQL----VQ-ASS----KP-LPPVPKICRIKASKALKKKIRRFQPASLKVMTMV
Tg_2         RQPARRAGIPRTVSMR-----------------------------SHSKGQ----HHGDCAKSESDHSEYSAECASLFHSTIAETSEGE-VSDFTANRFGDSESSEGDWDG--SSNSSSLALDYDEG-----DESE--LIWPEGSVRQS----VTVQAS---SKP-LPPVPKICRIKASKALKKKIRRFQPASLKVMTMV
Ap_2_ex2-4   KQPARRASVLRTLSMR-----------------------------ARSKSH----HHGAYARSESDHSEYSAECASLFHSTIAETSEGE-VSDFTANRFGDSESSESDSDG--SSNSSSLALDYDEG-----DESE--LIWPEGSVRQS----GTVQVS---SRP-LPPVPKICRIKASKALKKKIRRFQPASLKVMTMV
Mu_2         RQSARRAGVLRTVSMR-----------------------------ARSKSQ----RHGAYAKSESDHSEYSAECASLFHSTIAETSEGE-VSDFTTNRFGDSESSESDSEG--SSNSSSLTLDYDEG-----DESE--LIWPEGSVRQS----GAAQAS---SRP-LPPVPKICRIKASKALKKKIRRFQPASLKVMTMV
Ac_2         RPQGKRAGIMRAVTMR-----------------------------ARSKSRH-PFHPGTYAKSESDHSEYSAECASLFHSTIAETSEGE-VSDYTANRFGDSESSESDSEG------SSLTLDYEEEEI---EEHE--LIWPDSSVRQP----TSTQTS---SQP-LPSVPKLCRIKASKALKKKIRRFQPASLKVMTMV
Pym_2_ex2-4  KLQGRRAGILRTVTMR-----------------------------AESKKCH-PLHPGAYAKSESDHSEYSAECASLFHSTIAETSEGE-ISDYTTNRFGDSESSDSDSEGGSSSSGSSLTLDYGDE-I---HEHE--MVWPTSTGKQA----AATQVL---AKP-LPPVPKLCRIKASKALKKKIRRFQPTSLKVMTMV
Cpb_2        RQPVRRAGVLRTVSMR-----------------------------ARSKHHR----HGTYAKSESDHSEYSAECASLFHSTIAETSEGE-VSDYTTNCFGDSESSESDSEG--SSNSSSLTLDYDDA-----DESE--LVWPEASVRQP----AAVQAS---SKP-LPPMPKICRIKASKALKKKIRRFQPASLKVMTMV
Ps_2         RQPGRRAGVLRTVSTR-----------------------------AHSKHHR----HGTYAKSESDHSEYSAECASLFHSTIAETSEGE-VSDYTTNRFGDSESSESDSEG--SSSSSSLTLDYDDA-----DESE--LVWPETSVRQP---GAVHTSF----KP-LPPMPKICRIKASKALKKKIRRFQPATLKVMTMV
Pa_2_ex1-3   --------------------------------------------------------------------------------------------------------------------------------------------------------------------------------------------------------
Lc_2         KQHVRKASVLRTVSLK-----------------------------THQAHYRHSAYIRSSARSDSDQSEYSAECASLFHSTIVETSEDE-RSDYTTNRFGDSESSESDSDA-TSDSS--LTLDYDDT-----DASE--FVWPEASS-QPAASSQPAAASVAPSKQ-AHTEPKLCRIKASKALKKKIRKFQPASLKVMTMV
Lo_2         LQVLRKAGMMRSTSLR------------------------HRPSQ-YGVPRHPHPNSYSCLLSESEVSEYSAECASLFHSTIAESSEGE-LSDFTANRFGDSESSESSSDG-SSDSSLSLNSE-D---M---EDGE--LVWAQSTV-------GPTAAG---LPLSSRPEPPACRIKASKALKKKIRRFQPAALKVMTMV
Dr_2         -LNSRKVAMVRSVSARPRSGHWGCPRPRALPHSLSTSSCFSYLESRYPAAP---VSSRHPPRCESEFSEYSAECASLFHSTIAASSDGE-MSDYTTNRFGDSESSQ-GSQT-ASESDSSLSLDEED-LLEEEEEDEGGLVWAQAAM-------GTTAAGFS-LQQHHRSESAACRIKASRALKKKIRRFQPASLKVMTLV
Omo_2_ex1-3  --------------------------------------------------------------------------------------------------------------------------------------------------------------------------------------------------------
Gmo_2        --------------------------------------------------------------------------------------------------(gap)-----------------------------------LVWAQASSTKGPAVAGATAAP---QRPLHPGAPPTCRIKASRALKKKIRRFQPATLKVMTLV
Tr_2         ---QAQGSMQMVHMN-DRSGQWMGPPGSFQTPVSPHS-FLHNLNARYPPSPFH-MSRHYPPRCE---SEYSAECASLFHSTIAESSEGE-MSDNTTNRFGDSESSQ-SFLS-FSDSDSSLSLDEDYQVDNQGEERN--LVWAEDSL-------GPTAAG---LQQLPRPEPSACRIKASRALKKKIRRFQPASLKVMTLV
Tn_2         ---QAPGSMHMVRS--VKSGQWVAPPRSFQIHVPSHS-FLHNLNARYPPAPFH-MSNHYPPRCE---SEYSAECASLFHSTIAESSEGE-MSDNTTNRFGDSESSQ-SFQS-FSDSDSSLSIDEDDQVDSRGEDRD--LVWAEAAL-------GPAAAG---LRQLPRPEPSACRIKASRALKKKIRRFQPASLKVMTLV
Ga_2         ---QAPGSTQMVRSMSAKSSQWIGPHRSFQ---SSNP-FLHNLNTRYPPAAF-SISSHYPPRCE---SEYSAECASLFHSTIAESSEGE-MSDNTTNRFGDSETSQ-SFQS-FSDSDSSLSLDEEDQVY-GQEERG--LVWADAAL-------GPTAAGRP-LQQLPCPEPSACRIKASRALKKKIRRFQPASLKVMTLV
Ol_2_ex2-4   --------------------------------------------------------------------------------------------------------------------------------------------------------------------------------------------------------
On_2         -HALAPGSMQMVQSMSAKPGQWIRPARPFQSSVSSSS-FLHSLNAKYPPAPFH-VTSHYPPRCE---SEYSAECASLFHSTIAESSEGE-MSDNTTNRFGDSESSQ-SFQS-FSDSDSSLSLYEGEQADSHEEEGS--MVWAEAAL-------GPTAAGRPLKQLPPRPEPAACRIKASRALKKKIRRFQPASLKVMTLV
Xm_2         -------SMQMVRSL--KPGQWIGPSHPFHFSMSSNS-FLQSLNARYPPAPFL-MTSLYPPRCE---SEYSAECASLFHSTIAESSEGE-MSDNTTNRFGDSESSQ-SSQF-FSDSDSSLSLDEEEEEVEINEAAEGGMVWAEAAL-------GPTAAGQPLRQLPSRPEPTACRIKASRALKKKIRRFQPASLKVMTLV
Tc_2_ex3-4   --------------------------------------------------------------------------------------------------------------------------------------------------------------------------------------------------------
Cm_2_ex2-4   -GAVSQHKQQMGKA---ERGQN--------------------VSFRSRTFENH-FSVYSYARSESDQSEYSAECASLFHSTIVETSEDD-ESDHTTNCFGDGELSG-GSVS-DTASNSSLSLDDDDPA----------LVWPQGLV---AAN-EPTSSS---CQP-RHSEAQVCRIKASKALKKKIRRFQPDSLKIMTMV
Exons        --------------------------------------------------------------------------------------------------------------------------------------------------------------------------------------------------------
motifs       (11c)***------------------------------------------------------*************11d*******************-***11e**********************------------11f---------------------------------*****11g(PDZ-binding)*****
Leu zipper   --------------------------------------------------------------------------------------------------------------------------------------------------------------------------------------------------------

                     .
Hs_2
Mm_2
Bt_2_ex2-4
Cf_2
Md_2
Oa_2
Gg_2
Mg_2_ex2-4
Tg_2
Ap_2_ex2-4
Mu_2
Ac_2
Pym_2_ex2-4
Cpb_2
Ps_2
Pa_2_ex1-3
Lc_2
Lo_2
Dr_2
Omo_2_ex1-3
Gmo_2
Tr_2
Tn_2
Ga_2
Ol_2_ex2-4
On_2
Xm_2
Tc_2_ex3-4
Cm_2_ex2-4
Exons
motifs       -
Leu zipper
```

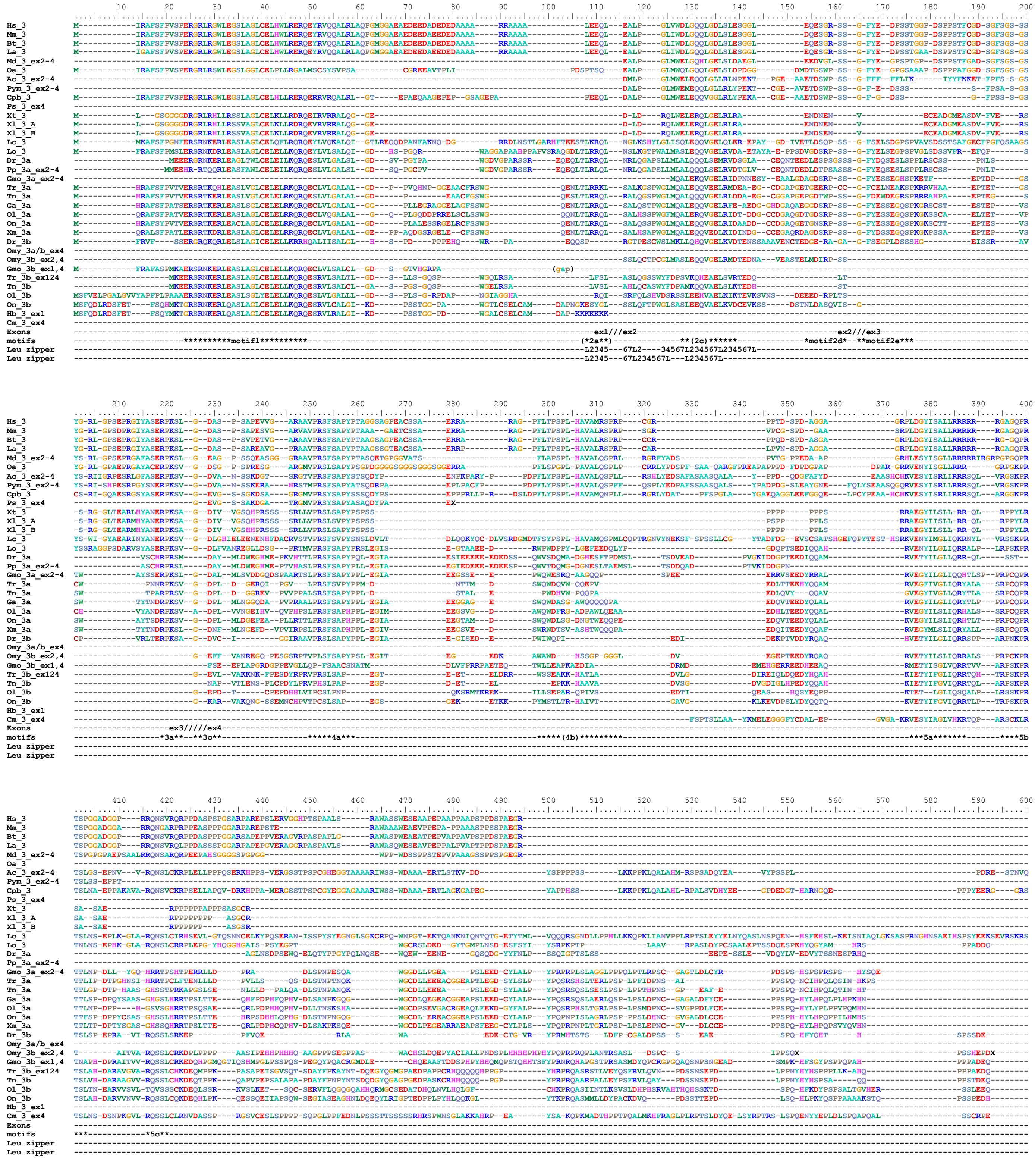

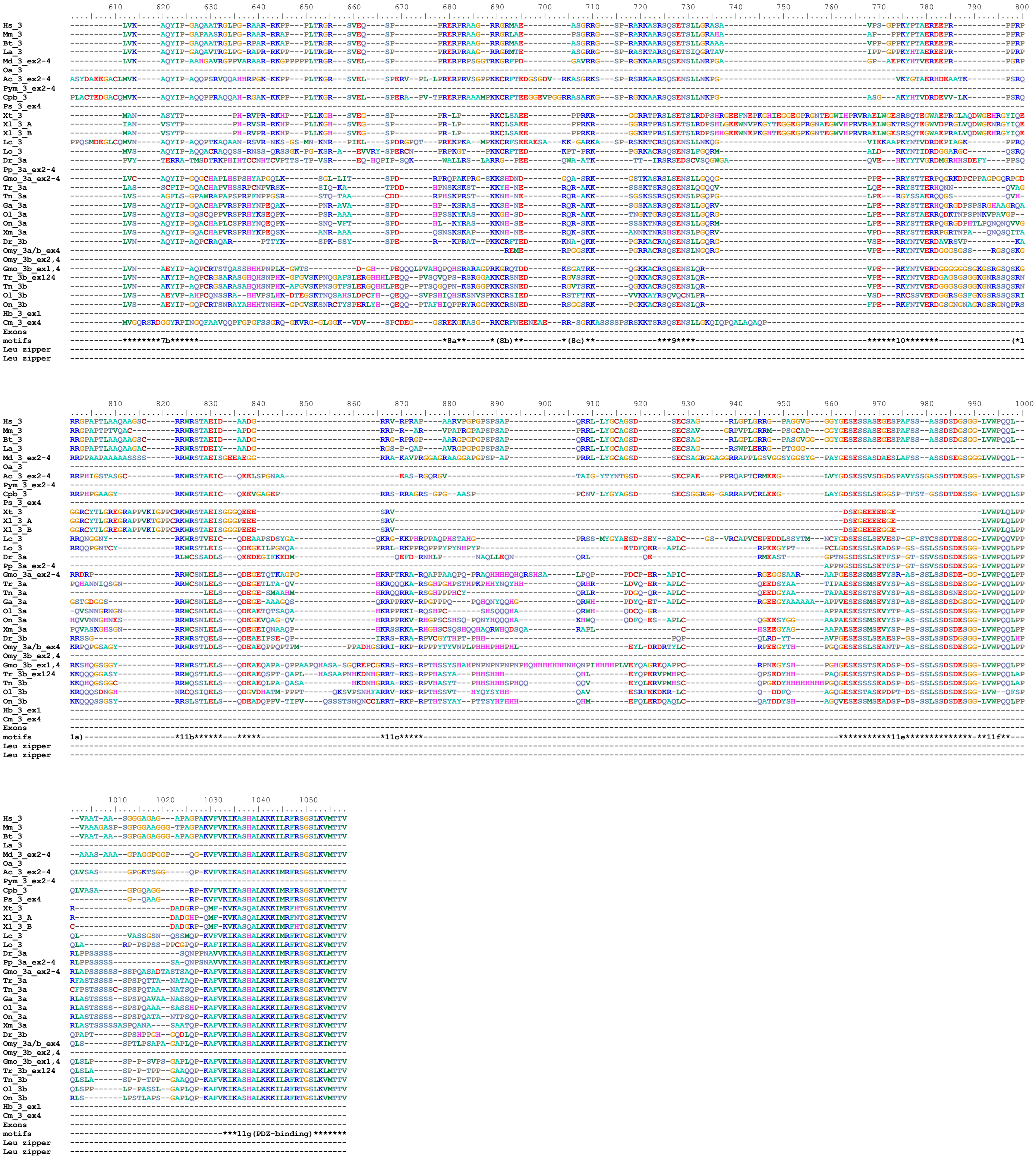
Hs_3
Mm_3
Bt_3
La_3
Md_3_ex2-4
Oa_3
Ac_3_ex2-4
Pym_3_ex2-4
Cpb_3
Ps_3_ex4
Xt_3
Xl_3_A
Xl_3_B
Lc_3
Lo_3
Dr_3a
Pp_3a_ex2-4
Gmo_3a_ex2-4
Tr_3a
Tn_3a
Ga_3a
Ol_3a
On_3a
Xm_3a
Dr_3b
Omy_3a/b_ex4
Omy_3b_ex2,4
Gmo_3b_ex1,4
Tr_3b_ex124
Tn_3b
Ol_3b
On_3b
Hb_3_ex1
Cm_3_ex4
Exons
motifs
Leu zipper
Leu zipper
11g(PDZ-binding)

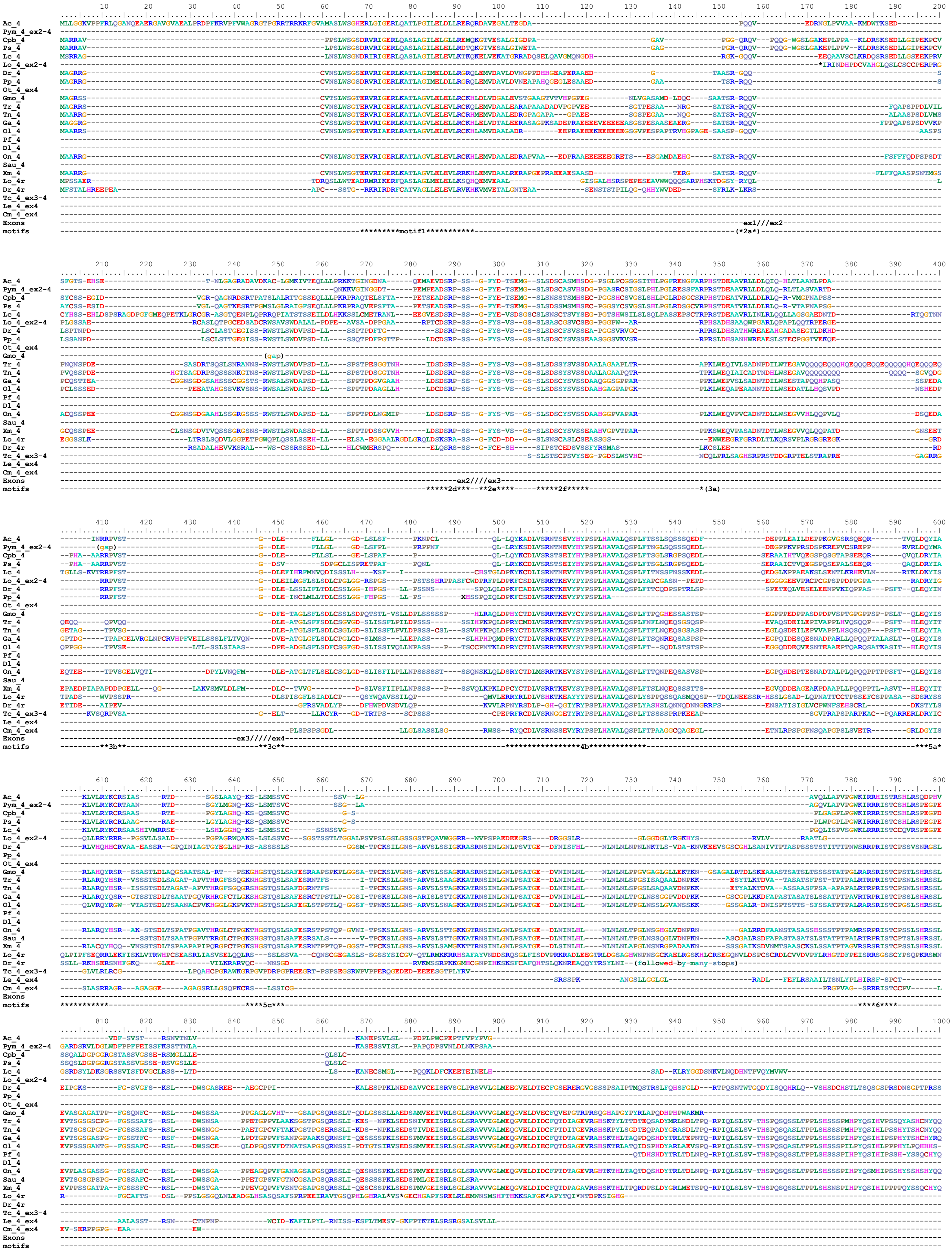
Ac_4
Pym_4_ex2-4
Cpb_4
Ps_4
Lc_4
Lo_4_ex2-4
Dr_4
Pp_4
Ot_4_ex4
Gmo_4
Tr_4
Tn_4
Ga_4
Ol_4
Pf_4
Dl_4
On_4
Sau_4
Xm_4
Lo_4r
Dr_4r
Tc_4_ex3-4
Le_4_ex4
Cm_4_ex4
Exons
motifs
motif1
ex1///ex2
ex2////ex3
ex3/////ex4
(gap)
(followed-by-many-stops)

```
                        1010      1020      1030      1040      1050      1060      1070      1080      1090      1100      1110      1120      1130      1140      1150      1160      1170      1180      1190      1200
                ....|....|....|....|....|....|....|....|....|....|....|....|....|....|....|....|....|....|....|....|....|....|....|....|....|....|....|....|....|....|....|....|....|....|....|....|....|....|....|....|
Ac_4            ------------------------SER-GLPHWTPS---------------------RKASSL-PQ-------------------------------EKLYRGKHMSRELVKRPERQEKTS--ERS--WPS----PRELL------NRLSLRRRLSPHAGNLACASS-----EMNVN------ATRHAS-----------
Pym_4_ex2-4     ------------------------PER-RALGWTAGAQQQIIKTETSF---------KKEASL-SRATPGV--------------------------DKLYKGKHASRELVKTAEREEKAA--ERS--WLS----PRELL------RRLSLRRKPSARAGGRARASS-----EMNVHAAAGLVGPSHLQA----------
Cpb_4           ------------------------REQPEGVGWAEALSCHPHVVR------------VKASSR-KALGTER--------------------------ERLYKGKHASRELVKAGEGA------ERS--WLS----PRELL------RRISLRRRPALRAEGGSRCSS-----EVNVRTPRP-VYRCSSQH----------
Ps_4            ------------------------REQPEGASWAEASSCLTHVVR------------VKASSR-KGLGCEQ--------------------------ERLYQGKHASRELVKAREGA------ERS--WLS----PRELL------RSPRAGGGSR--------CSS-----EMNVRMPGP-VYRCSSQR----------
Lc_4            ---------------------------------------------------------RKSTHR-CNS---------------------LRWV-SADMDALYKGKHASRELVKAATLSNNYESAEKTNSIIK-----KDIF------RRISLRKKPSAREDNRSRSRS-----EMNLNMA---VTKKSSALYKCESQ----
Lo_4_ex2-4      --------------------------------------------------------------------------------------------------------------------------------------------------------------------------------------------------------
Dr_4            PILPFPHNQSDPGSSPGSSHT-RVHSPPRLLSNSPFTPAQLSVFRRDSPSQCSLPRFHPCSSPLEGP-IRPRGGSLRQNTGAGWRPQDRGWRRSGDGERLYQGKHASRELIRASTVSSYSGRD-YGSGWED------EM-STQT--PKKGG-KFWKGFETRFWGREEEDDDDEDE-RDV-----RAGGFSWR--HTS-LK
Pp_4            --------------------------------------------------------------------------------------------------------------------------------------------------------------------------------------------------------
Ot_4_ex4        ----------------------------------------------------------------------------------------------------------------------------GSSNWGE----EGSHDTAQT--PKKGADKLWRDFEGHSWGKEAEEENKERWKRE---GIRRKK-EIYQKEKQQANK
Gmo_4           ---------------------(gap)---------------------------------------------------------------GWRR-SEGEGLYRGRHASRELVRASPVSSYAHRDNYGSTWGEDERGEGEPRPREASAPKRTSGRLWRGWDGRLWGRESDDDREEVDRAEYGYGWSRS--GSWRRECRR-GQ
Tr_4            IPSSS-DLPSSTASSPASHPNRRSHSPPRPLQPSPLGSTPLSVFRRDAPFQCSLPRTNTRSSPFDHGGIHPRGGSLRQSGAS--PEGSGGWKR-TEGEGLYRGKHASHKLIRAATVSSYAKRENYNSIWGE----EQEPSTVQT--PKKNN-KIWKGFEARLWGKDSECDKEEMDRAEYGYSRRRNSVGSWRKEHQR-LK
Tn_4            IPSTS-DLPSSTASGPASHPNRRSHFPPRPLQPSPLGSTPLSVFRRDAPFQCSLPRTNTRSSPFDHGGIHPRGGSLRQSGAS--NEGSGGWKR-AEGEGLYRGKHASHQLVRAATVSSYAKRENYSSIWGE----EREQPAVRT--PRKTSGKIWKGFETRLWGKDSESDREEMDRAEYGYGRRRNSIESWRKEHRR-PK
Ga_4            VPSPS-DHPSSTASSPASRPTTRGRSPPRPLQPSPLGATPLSVFRRDAPFQCSLPRTSTRSSPSEHGGIQPRGGSLRQNGGG--SGGGSGWKR-VEGEGLYKGKHASHNLVRASTVSSYAKREDYSSDWAEGE--ERERSAAQT--PRKTSSKLWKGFEGRLWGKESESEREEMDRAEYGYGWRRNSVGSWRKEHRR-GK
Ol_4            -PSPS-ELPSSTCSSPASHPIPRSHSPPRPIKPSLLGSTSLSVFRRDSPFQCSLPHGNTRTSPLEHKGIQPRGGSLRQSGGG--GDGASRWVR-MEGEGLYRGKHASHKLVRAATVSSYARREDYSSVWAE-E--EKIRSAAET--PRKSPSKLWRGLEGRFWSKESESEKEEMDRAEYGYNMMRSRAGSWRRDQRR-MK
Pf_4            QIPSPSDLPSSTASSPASHPTTRGRSPPRPLQPSPLGATPLSVFRRDAPFQCSLPRTNTRSSPLEHGGIQPRGGSLRQSGGS-----GGGVRR-VEGEGLYRGKHASHKLIRAATVSSYAKREDHSSAWAGEQ--ERERSSAQT--PRKTSKKLWRGFEGRIWSKASESEREEMDRAEYGYGWRRNSIGSWRREHRK-MK
Dl_4            -----------------------------------------------------------------------------------------------------------------------------------------DQQPD----TQEDLHKLWRGFEGRLWGKESESEREEMDRAEYGYGWRRNSIGSWRREHRR-WK
On_4            TP----DLPSSTASSPASRPTPRGRSPPRPLQPSPLGATPLSVFRRDAPFQCSLPRTNTRTSPFEHGGIQPRGGSLRQSSGG--GEGNSRRKR-AEGDGLYRGKHASHKLIRAATVSSYAKREDYSSVWGEEE--EKSRAAAHT--SKKSSSKLWKGFEGRLWSKESESEREEIDRAEYGYGWRRNSVGSWRREHRR-VK
Sau_4           --------------------------------------------------------------------------------------------------------------------------------------------------------------------------------------------------------
Xm_4            HIPSPSDLPSSTASSPASHPTPRARSPPRPLKPSPMGATPLSVFRRDSPFQCSLPH-NNRTSSWEHGGLQPRGGSLRHSGGG--GEGGGRWKKV-ERDGLYRGKHASHKLVRAATVSSYARREDYSSVWAE-E-------AAHT--PRKSSSKLWRGFEGRLWRKESETEKEEMDRAEYGYGWSRGNMGSWRREHRR-AK
Lo_4r           --------------------------------------------------------------------------------------------------------------------------------------------------------------------------------------------------------
Dr_4r           --------------------------------------------------------------------------------------------------------------------------------------------------------------------------------------------------------
Tc_4_ex3-4      --------------------------------------------------------------------------------------------------------------------------------------------------------------------------------------------------------
Le_4_ex4        -------------------APRSPRILRSRDGLLLLLRDII*NPFTGVNQLEVN*RPRSIVVRMTLLV-------WTTTS-------------------LIRLHHTSSR-------------------------------------------------------------------------------------------
Cm_4_ex4        ---------------------------------------------------------------------------------------------------LYRGRHLSHRLVRCPETLGC----GRS---VS----QAEL-------GPVGGGGYSRPGLECGLA--------EPGPR-----------------------
Exons           --------------------------------------------------------------------------------------------------------------------------------------------------------------------------------------------------------
motifs          ---------------------------------------------------------------------------------------------------****10*******-------------------------------------(11a-1)--------------------------------------------

                        1210      1220      1230      1240      1250      1260      1270      1280      1290      1300      1310      1320      1330      1340      1350      1360      1370      1380
                ....|....|....|....|....|....|....|....|....|....|....|....|....|....|....|....|....|....|....|....|....|....|....|....|....|....|....|....|....|....|....|....|....|....|....|....|....|...
Ac_4            -------------------SKGKPVKPKWTSVMEISSRTPPRPQL-FRPPP-----MLAHH---------LAFSSDSPNSFCF-LEGFE-RSLTATT-----SSS-SLAKVEATSAINLNGDWILQQLGD-----GWPHT-A--DSTDVPVRRVKLHRSRSFKEFKKAMSRSLRG--SRATK------
Pym_4_ex2-4     -------------------SRGKAVKPKWTSVLEISSKTAGRHRG--KAGTF----LP--------------(gap)--------------------AGLAGSGS-SLSEVEATSAVSLNGDWVFHQLGD-----RWLHGSATLDLADLPAPNFKLRRSRSFKELKKIVSRSFKS--SRTAKY-----
Cpb_4           -------------------CLEPPGRHKWASVLEISSQALGRPKA-EPFPP-----PS-------RQ---LAFSTDSCFPDGSQLELQPQRAE--------GSSASSLSEVDA---VSLNGDWRLRQLGPPRA--RPLHASATLDSSNLAMASFKLHRTRSFKELKRMVSRSLRPLAPKASGASK---
Ps_4            -------------------CLEPPGRHKWVSVLEISSQALERPKA-EPFPP-----PS-------RQ---LAFSTDSCFPAGSQLALQPPRAE--------GSSASSLSEVDV---LSLNGDWRLRQLGPPRA--RTLQASATLDSSDPAMASFKLHRACSFKELKRMMRMKRPLV-PKASGASK---
Lc_4            DTLSEAFGKKG-K-TNE--WKKKPKKQKWMSVLEISNRTPETFGFSQSRTRKSG--FFSQAQAFLSR--HRAFSTDCPSSFHSPILEPSQTDLQNTTAHYLYGSESSLSEVDATSCTSLNRELIHEDRSIARPHLGEKFFSNPLDNKNMVNGSIQLYRTKSFKELKKRVSRSMRPFSFKGTSSTK---
Lo_4_ex2-4      --------------------------------------------------------------------------------------------------------------------------------------------------------------------------------------------
Dr_4            ETSSSRQDHRG---NN---SKKKGTS---WDSRSSSLRLSRRALFRSESQG-ILE-PHSQKRDSN----QWRSSFEISRAGRGSER-ARNLVRK---EEKHHSSTASLF--------HLS----RSQSLEGSSHSLS--PLSSPSLSPSPPPSAPLTRSRSFRDLGRKVFGSMRSLSFNKNHKSKKKD
Pp_4            --------------------------------------------------------------------------------------------------------------------------------------------------------------------------------------------
Ot_4_ex4        ET-----ERRQK-ESS--ASKGKGKGGGGWDKRSSSLRLSRQALFRSESQR-EVLDPRALKEEHLRGA-QWTSSLDIGRGMELSVEGVRPLVFGGRAEEERLSSTASLF--------HLS----SSQSLDDSCPSLSP-PLSSPSFSPSPPSRAPLTRSRSLRDLSRRVFS-----------------
Gmo_4           PMTGSCKEKRPTVDSSPSSSSAAAKGKGGWEKRSSSLRLSRT-LFRSESQG-LLV-PRARRQEPLRAT-PWVSSLDVARARLQLEPEDGVRPLDGD-GDKRLSSTASLF--------NLS----RSQSLEGSCQSLSP-PLSSPSFSPSPPPRT-LQRS-----------------------------
Tr_4            ASSSSN-DRRSKFQNSPMFSKRRGKEEG--ERRSSSLKLSRRALFRSESQG-LLV-PRNHSEETTKQA-HWVSSLDVAQGGLYVSRDEAVRLLSTKGEDKRLSSTASLF--------NLS----RSQSLEGSCHSIS--PLSSPSFSPSPPSRMPLQRSRSLRDLGRKVFGSMRSLSLKRKPSKK---
Tn_4            ASSGSN-DRRAKIQSSPMFSKKRGKEDG--ERRSS-LKLSRRALFRSESQG-LLV-PRNHGEETAKRAAHWVSSLDVAQGGLYVSRDEAVRLLSTKGGDKRLSSTASLF--------NLS----RSQSLEGSCHSLS--PLSSPSFSPSPPSRMSLQRSRSLRDLGRKVFGSMRSLSLKRKPSKK---
Ga_4            ASSSSN-DKRPKVENSPLFSKKRGKEDG--ERRSTSLRLSRRALFRSESQG-LLV-SRNHGEEPPKRA-HWVSSLDVGQGGVGLSKYEGARPPRA-EEDKRLSSTASLF--------NLS----RSQSLEDSCHSLS--PLSSPSFSPSPPPRMPLQRSRSLRDLGRKVFGSMRSLSLKRKPSKK---
Ol_4            VSG----DRKPKVDNSPMLLRKRGKEDG--ERRSSSLRLSRRALFRSESQG-LLV-PGNHSEGATKRA-HWVSSLDLGQGSMGVRRDEGVHLLRAKGNDKRLSSTASLF--------NLS----HSQSLEGSCQSLS--PLSSPSFSPSPPPRMPLQRSRSLRDLGRKVFGSMRSLSLKRKPPKK---
Pf_4            ASSSNN-DRRPKVENSPIFSKKR---------------------------------------------------------------------------------------------------------------------------------------------------------------------
Dl_4            ASSSSN-DKRPKVENSPIFSKKKGKEEG--ERRSSSLKLSRRALFRSESQG-LLV-PRNHSEEHSKRA-HWVSSLDVGQGGMYISKEEGVRLLRAKEEDKRLSSTASLF--------NLS----RSQSLEGSCHSLS--PLSSPSFSPSPPPRMPLQRSRSLRDLGRRVF------------------
On_4            ASSSG--DKRPKVDNSPVFSKKRGKDDG--ERRSSSLRLSRRALFRSESQG-LLV-PRNHSEEPTKRP-HWVSSLDVGHGGMGIRKDEGVRLLRAKEEDKRLSSTASLF--------NLS----RSQSLEGSCHSLS--PLSSPSFSPSPPPRLPLQRSRSLRDLGRKVFGSMRSLSLKQKSSKK---
Sau_4           --------------------------------------------------------------------------------------------------------------------------------------------------------------------------------------------
Xm_4            GSS----DRKPKVDHSPVFSRSRGKEDG--ERRSSSLRLSRRALFRSESQG-LLV-PRSHREELAKRG-HWVSSFDVAQGGTS-RDEGVRLLRAK-EEDKRLSSTASLF--------NLS----RSQSLEGSCQSLS--SLSSPSFSPSPPPRLPLQRSRSLRDLGRKVFGSMRSLSLKRKPSKK---
Lo_4r           --------------------------------------------------------------------------------------------------------------------------------------------------------------------------------------------
Dr_4r           --------------------------------------------------------------------------------------------------------------------------------------------------------------------------------------------
Tc_4_ex3-4      --------------------------------------------------------------------------------------------------------------------------------------------------------------------------------------------
Le_4_ex4        --------------------------------------------------------------------------------------------------------------------------------------------------------------------------------------------
Cm_4_ex4        -------------------VKRRG----WASALEISVS-LDPQAQAQAPRR-----P-----------------------------------------------------------------------------------------------------------------------------------
Exons           --------------------------------------------------------------------------------------------------------------------------------------------------------------------------------------------
motifs          -------------------(11a-2)-***(11b)*-------------------------------------------------------------------------------------------------------------------------********(11g)***************---
```
