# Supplementary material for: Dact genes are chordate specific regulators at the intersection of Wnt and Tgf-β signaling pathways
Source: BMC Evol Biol. 2014 Aug 6;14:157. doi: 10.1186/1471-2148-14-157 (PMC4236578; doi:10.1186/1471-2148-14-157)
Supplement: Additional file 11 — Gnathostome Dact protein sequence logo. [file 1471-2148-14-157-S11.pdf]

Additional File 11. Gnathostome Dact sequence logos.

Sequence logos of the four gnathostome Dact paralog group proteins, based on the gapped alignments shown as Additional File 10.

gnathostome Dact1 consensus

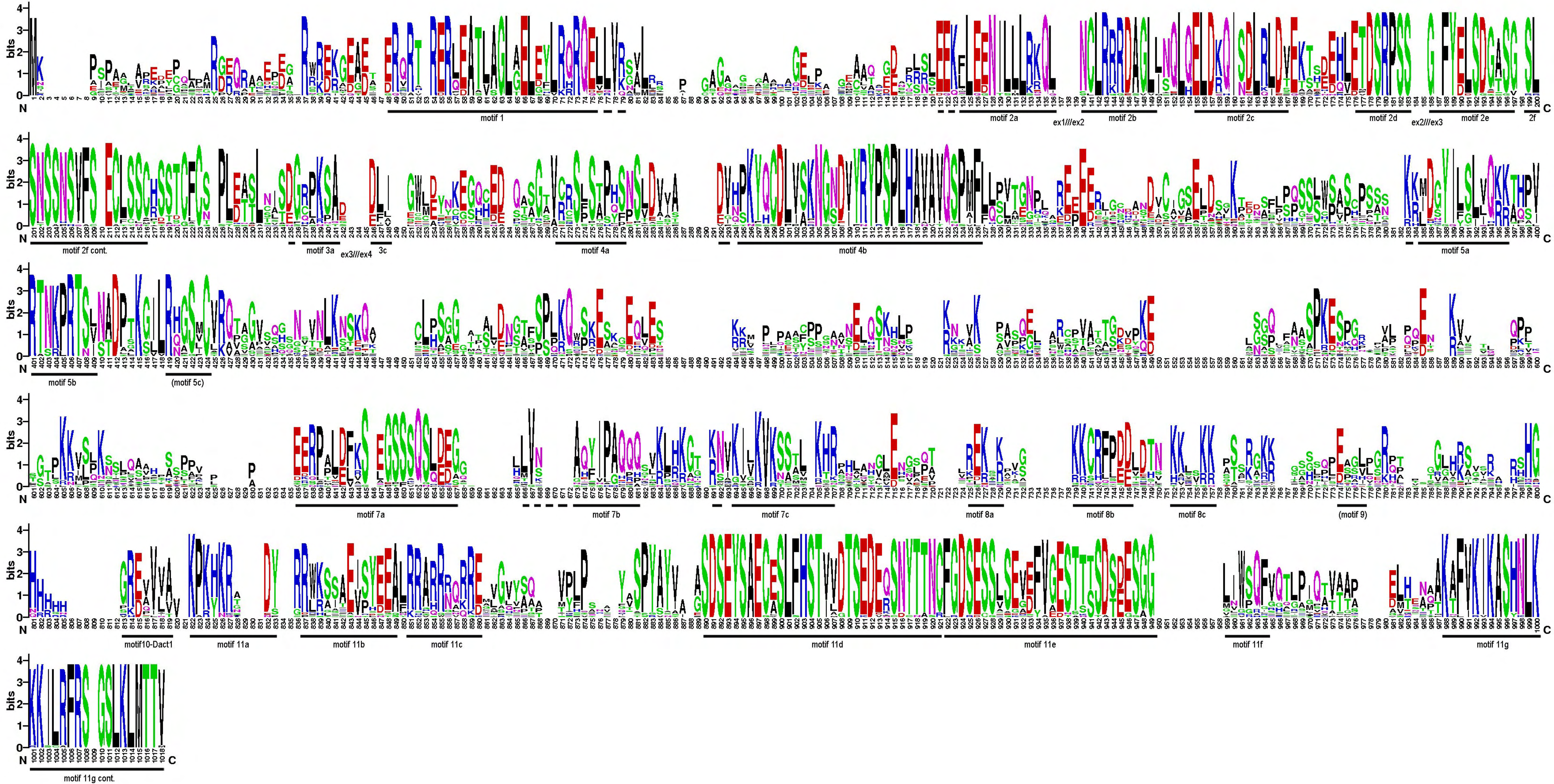

gnathostome Dact2 consensus

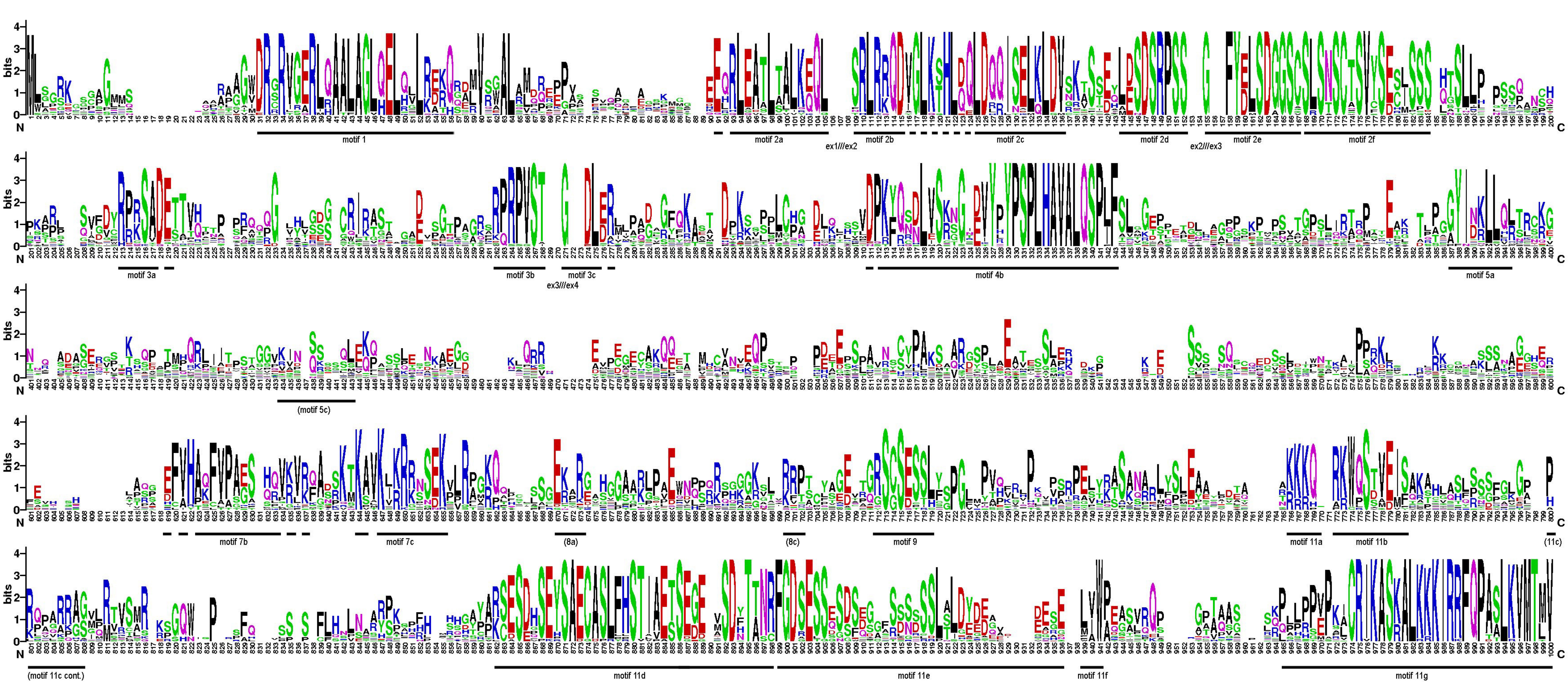

gnathostome Dact3 consensus

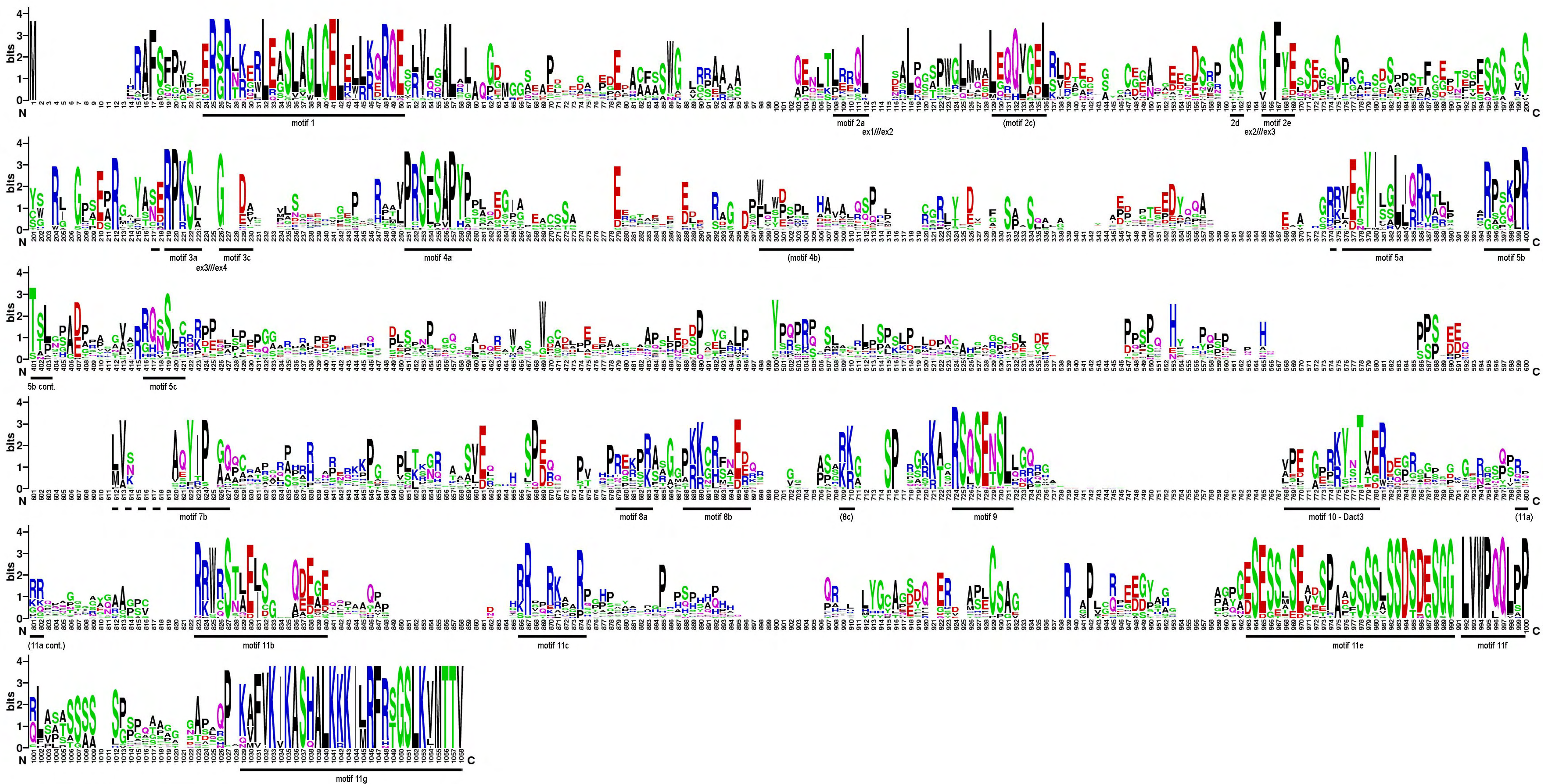

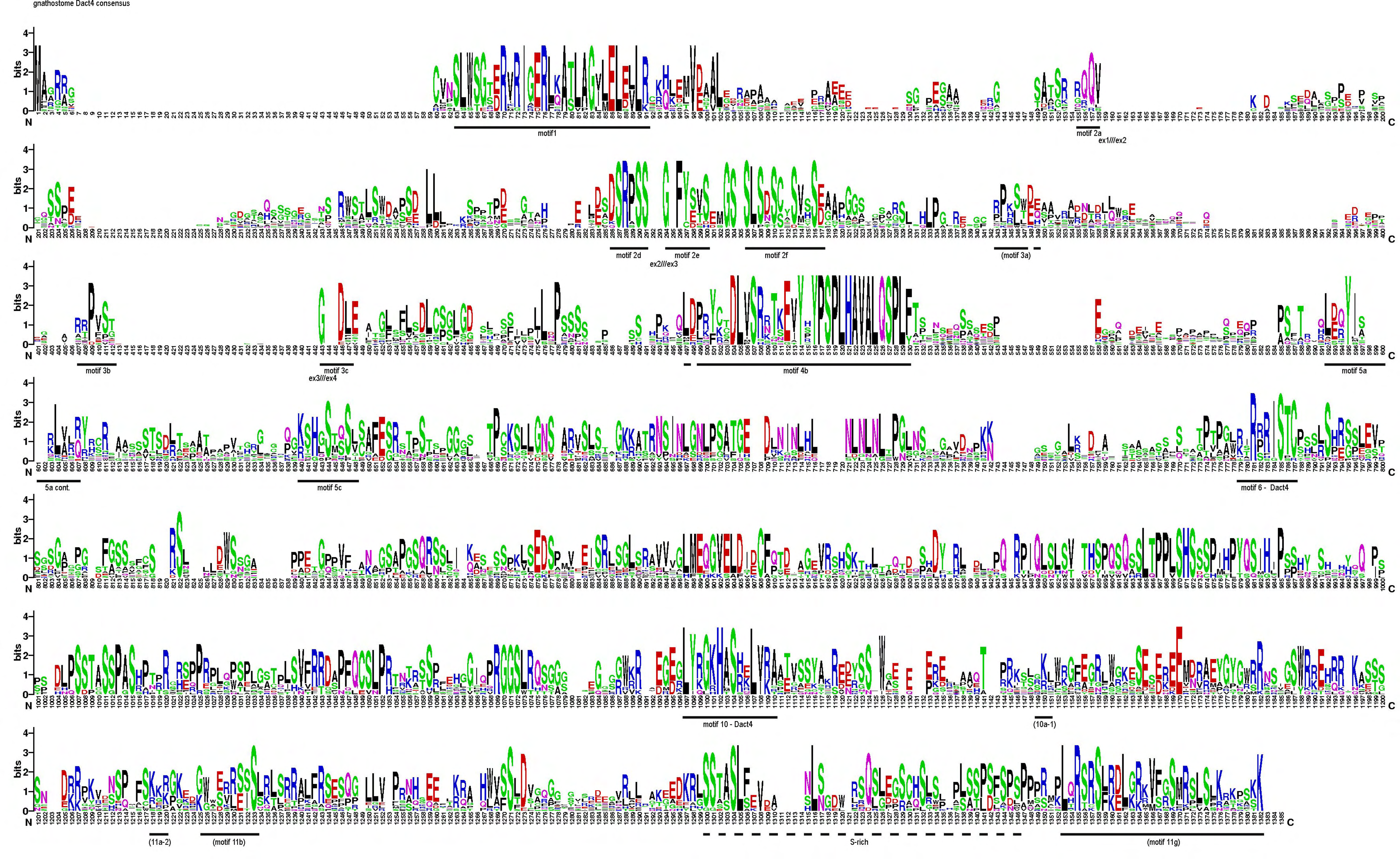
gnathostome Dact4 consensus
bits
motif1
motif 2a
ex1///ex2
motif 2d
motif 2e
motif 2f
(motif 3a)
ex2///ex3
motif 3b
motif 3c
ex3///ex4
motif 4b
motif 5a
5a cont.
motif 5c
motif 6 - Dact4
motif 10 - Dact4
(10a-1)
(11a-2)
(motif 11b)
S-rich
(motif 11g)
