# Supplementary material for: Dact genes are chordate specific regulators at the intersection of Wnt and Tgf-β signaling pathways
Source: BMC Evol Biol. 2014 Aug 6;14:157. doi: 10.1186/1471-2148-14-157 (PMC4236578; doi:10.1186/1471-2148-14-157)
Supplement: Additional file 12 — Arrangement of the leucine zipper. [file 1471-2148-14-157-S12.pdf]

Additional File 12.

Arrangement of the leucine zipper in gnathostome Dact1, 2 and 3, and in cyclostome and *Branchiostoma* dact proteins.

```
    L234567L234567L234567L234567L234567L234567L23                     L for L-zipper   gene
    LEENILLLRKQLNCLRRRDAGLLNQLQELDKQISDLRLDVEKTS                            6          Hs   DACT1
    LEAALAALQEQLSRLRQQDIGLKTHLDQLDLQISKLQLDVGTAS                            6          Hs   DACT2

    L234567L234567L2....345...67L234567L234567L234567L234567L         L for L-zipper   gene
           LEEQLEALP....GLV...WDLGQQLGDLSLESGGLEQESGRSSG                    5          Hs   DACT3
     (gap)          DMLP....GLM...WELEQQLGLLRLNPEKTPGEAAETDSWPSS            3          Ac   Dact3
           PEEQLDALP....GLM...WELEQQVGGLRLYPEKACGEAAETDSWPSS                3          Cpb  Dact3
            LQGGEDLD....RQL...WELERQLGELRLRAENDNEN                          3          Xt   dact3

    L234567L234567L234567L2...67L234567L234567L234567L234567L         L for L-zipper   gene
 GARHFTEESTLKRQLNGLKSHYLGLI...SQLEQQVGELQLEREPAYGDIVETLDSQPSSG          3+2        Lc   Dact3
 PVSRAQGDLTLRRQLNSLKGTPWALM...ASLEQQVGELRVDAETAYAEPPSDVGDSRPSSG         3+2        Lo   dact3
     EQEQLTLRRQLNRLQGAPSLLM...LALQQQLSEMRVDSGLACEQNTEEDLESPSGSSSG       3          Dr   dact3a
      QESLTLRRQLRALQGSPWGLM...QALQQQVGELRLDDAEGCGGAPGEPGDTWPSSG         3+2        Tn   dact3a
      QQNLTLRRQLSALHSSPWGFM...QALERQVGELRIDTDDGCCDGAQGDTGDSRPSSG        2+2        Ol   dact3a
      QENLTLRRQLSALQSSPWGLM...QALEQQVGELRIDAADDCCDGAQGETGNSRPSSG        3+2        On   dact3a
           EQQSPRGTPESCWSLM...KLLQHQVGELKVDTENSSAAAVENCTEDGERAGAG         2        Dr   dact3b
         RSALVSLAHLQCASWYFD...PAMKQQVAELSLKTEDHST                        -         Tn   dact3b
             RQISRFQLSHVDSR...SSLEEHVAELKIKTEVKSVNSDEEEDRPLTS             2        Ol   dact3b
     DAPNGKESYGLSSLQFTPWGLS...ASLEEQVAELKVDCEVKSSDSTNLDASQVISG          2+2        On   dact3b

 L234567L234567L23L234567L234567L234567L234567L34567L                 L for L-zipper   gene
LGGRDWLYALLVGPHLARQHDPACGTAGSVQLLEQRFCELGLRGEGGPPAAAG                     2        Lj   dactA
LERDFVESLRAMKEQLGCLPQRDLGLLSRLRHLNEQFSELKMDMDRAA                        2+4        Lj   dactB
                       QCGLGKPCALERPLSELRVDTDGT                         ?+2        Lj   dactC
ALLAVGRDLELIKEQL                                                        3+?        Lj   dactC/D

    L234567L234567L234567L234567L234567L234567L234567L234567L         L for L-zipper   gene
PGGCINSSIDKLRNAVDHLKRTDSDLVGALHTLNTQILQLKLAEAAETDGENDLMSASCRSMSSS       5          Bfl  dact

  exon1-encoded                 exon2-encoded
```

Amino acids are displayed using the 1-letter code. A template with L correctly placed for a L-zipper is shown above each set of sequences. Abbreviations: see Additional File 1.

Green highlighter, end of exon 1 - encoded sequences
orange L, L encoded by 1st exon
red L, L encoded by 2nd exon

L contributing to a continuous or 1st part of a bipartite L zipper are highlighted in blue, L contributing to the 2nd part of a L zipper are highlighted in pink.

In gnathostome Dact1 and Dact2 proteins, the exon1-2 splice generates a continuous 6x leucine (L-) zipper (top).

In coelacanth (Lc) and actinopterygian dact3 proteins, 3 amino acids having been lost, leading to a bipartite motif. In most teleost dact3b sequences, conversion of L into other amino acids further reduced the L-zipper. In tetrapod Dact3 proteins, additional four amino acids have been shed, restoring a reduced but continuous L-zipper.

In cyclostomes, dact A carries a small, exon 2 encoded L-zipper, and dact B a bipartite L-zipper. For dact C and D, exon 1 cannot currently be allocated, hence the state of the L-zipper is unclear.

In Branchiostoma dact, a continuous 5L zipper is found.
