# Supplementary material for: Dact genes are chordate specific regulators at the intersection of Wnt and Tgf-β signaling pathways
Source: BMC Evol Biol. 2014 Aug 6;14:157. doi: 10.1186/1471-2148-14-157 (PMC4236578; doi:10.1186/1471-2148-14-157)
Supplement: Additional file 13 — Alignment of Dact protein sequences. [file 1471-2148-14-157-S13.pdf]

Additional File 13. Alignment of Dact protein sequences.

Alignment of protein sequences for all Dact genes used in the phylogenetic analysis. Gaps are indicated by ‘-‘, missing sequences by ‘X’. Also shown is the position of the conserved protein motifs.

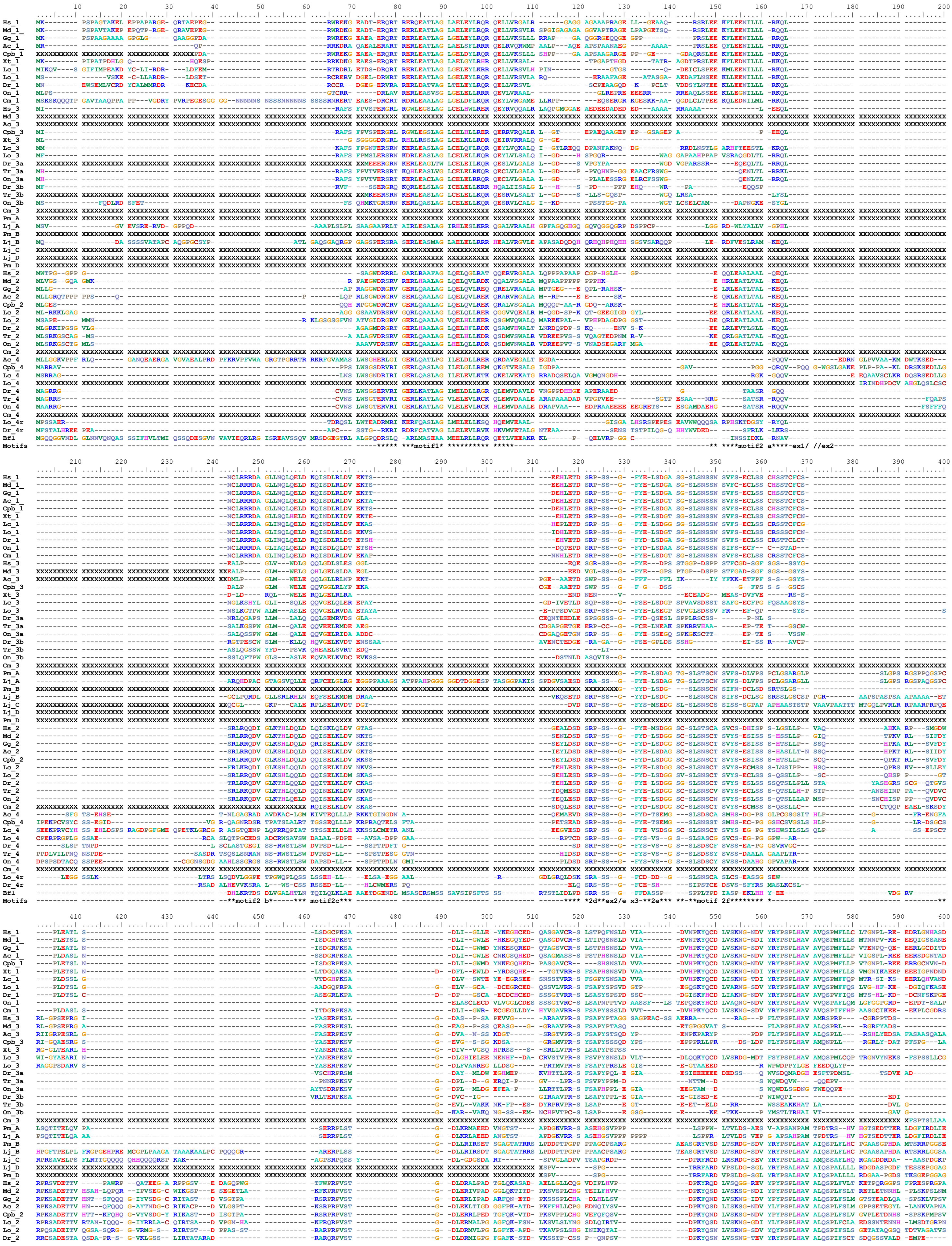

```
Tr_2     HQRSADESTT QPHP-PRAV- G-LHLGSS-- RIRVN---AE ---------- ---------- -QKRPRPVST ---------- ---------G- -DLDAMLTQG LGYFM-FVD- QRK-PLMCPK L--KSSTV-- ---------- ---DPKFQSN LVSRTG-AEV YPYPSPLHAV ALQSPIFSVG GEPTIPGLLE GQGPPTNS--
On_2     RRRSADENTT QPNP-PRGS- G-LHLGSS-- RIRASATGSE ---------- ---------- -QARPRPVST ---------- ---------G- -DLDRMLAQV PGYHK-SVD- AKK-PPMCPN L--KTSTG-- ---------- ---DPKFQRN LESRIG-TEK YHYPSPLHAV ALQSPIFSNG GEASMYGLLE GQGPPGN---
Cm_2     RPRSADETTV QATD-FRRR- G-VFIRPGR- GIRTTIEPVS LMAKKSG--- ---------- -VLRQRPVST ---------- ---------G- -DLERIRPTS RGFPS-ASD- LKQLSSMCTA DHLRLRLA-- ---------- ---NQRYRCD LVSKNS-SDV YNYPSPLHAV ALQSPLFSLT GE--APGF-- ----------
Ac_4     RPHSTDEAAV RLLDLQIQ-H LTLAANLPDA ---------- ---------- ---------- --INRRPVST ---------- ---------G- -DLE------ FLLGL----- GDLSLSF--- ---PKNPCL- ---------- -QL-LQYKAD LVSRNT-SEV YHYPSPLHAV ALQSPLFTSS LSQSSSQEDF ----------
Cpb_4    RPHSTDEATV RLLDLRLQ-R -VMGPNAPSS ---------- ---------- -------PHA --AARRPVST ---------- ---------G- -DLE------ FLLSL----- GELSPAF--- ---P------ ---------- -QL-LQYKCD LVSRST-SEI YHYPSPLHAV ALQSPLFTSG LSRGPSQEDL ----------
Lc_4     RPRSTDEAAV RLINLRLQQL LAGSGAEDNT D--------- --------RT QGTNNTGLLS -KVTRRPFST ---------- ---------G- -DLEFIHRFM NVQDISSSL- H---KSF--- I--------- -------CHS TGLDPKYKCD LISRNT-NEV YHYPSPLHAV ALQSPLFITN SSFNSSKEDL ----------
Lo_4     RPHSADHSAA QWPGARLQPA PLQQTRPERG E--------- ---------- ---------- ----RRPVST ---------- ---------G- -DLEILRGFL SLSDLCPGL- GGRSPGS--- ---PSTSSHR PPASFCWDPR FPLDPKFCSD LVSRKT-KEV YPYPSPLHAV ALQSPLYAPC GASN--PEPD ----------
Dr_4     RPRSLDHSAT HWREAEAHGA DASEGTLDKH D--------- ---------- ---------- ----RRPFST ---------- ---------G- -DLE-LSSLI FLTDLCSSL- GGIHPGS--- LL-PSSNS-- -------SPQ LQLDPKFCAD LVSRKT-KEV YPYPSPLHAV ALQSPLFTTC QDPSPTRLSP ----------
Tr_4     APKLWEQIVL SADNPDILWT EGAVQQQQEQ QQHQEQQQEQ QEQQQQQHQE QQQEQQEQQ- -----QPVQQ ---------- ---------- -DLE-ATGLS FFSDLCSGV- GDSLISSFLP ILDPSSS--- ----SSIHPK PQLDPRYCMD LVSRRT-KEV YSYPSPLHAV ALQSPLFNFL NQEQSGSQSP ----------
On_4     PLKLWEQVPV CADNTDLLWS EGVVHLQQPV LQ-------- ---------D SQEDAEQTEE -----TPVSG ELVQTIDPYL VNQFM----- -DLE-ATGLT FLSELCSGL- GDSLISFILP LLNPSSSSST ----SSQNSK LQLDSRYCTD LMSRRT-KEV YSYPSPLHAV ALQSPLFTTQ NPEQSASVSP ----------
Cm_4     XXXXXXXXXX XXXXXXXXXX XXXXXXXXXX XXXXXXXXXX XXXXXXXXXX XXXXXXXXXX XXXXXXXXXX XXXXXXXXXX XXXXXXXXXX XXXXXPLSPS PSGDL----- ---------- LLGLSASSLS G--------R WSS--RYQCD LVSRNS-SEV YRYPSPLHAV ALQSPLFTPA AGGCQAGEGL ----------
Lo_4r    ----WEEGRF GRRDLTLKQR SVPLRGRGRE GK-------- ---------- --GRDTPADS ----WVPSSP R--------- ---------- -DLSPISGFL SIADLCP--- -QSYWQAVSS ILQP------ ---------Q MVLERRYRLD LVSHKT-KEA YYYPSPLHAV ALQSPLYSPP QSSQASMQQS P--TDQLNEE
Dr_4r    ---------- ---------- ---------- ---------- ---------- -EERDETIDE ----AIPEV- ---------- ---------- -------GFR SVADLYP--- -DFHWPDVSD VLQP------ ---------K VVLRPNYRSD LP-GH--QGI YRYPSPLHAV ALQSPLYASH SLQNNQDNNG RRFSENSATI
Bfl      STGWPDEIIR EPAP-ITNSQ F-HNHRHSF- ---------- ---------- ---------- ---------- ---------- ---------G- -ESDSVASND AKQSYFSGLM PVQSQAPR-- EEKKKDDSLS ---------- --RRFKLALR PRSKTGLSVC YPYPSPMHAI AIQSDMYVPA ---------D CESVVEEEQS
Motifs   *3a****--- ---------- ---------- ---------- ---------- ---------- ---***3b** ---------- ----ex3/ex 4-3c------ ---------- -----**mot if4a***--- ---------- -********* ********** ***motif4b *******--- ---------- ----------

                610        620        630        640        650        660        670        680        690        700        710        720        730        740        750        760        770        780        790        800

Hs_1     ICGGSELDAV KTDSSLPSPS SLWSASHPSS S--------- ---------- ---------- ---------- ---------- ---------- ---------- ----KKMDGY ILSLVQKKTH P--------- ---------- ---------- ---------- ---------- ---------- ---------- ----------
Md_1_    ACIGSELDSV KSDNSSLPQS NSTSVSYPAS N--------- ---------- ---------- ---------- ---------- ---------- ---------- ----KKIDGY ILSLVQKKTH P--------- ---------- ---------- ---------- ---------- ---------- ---------- ----------
Gg_1     VCATSETDSG KATDAFLPPG S-WPASCPSA S--------- ---------- ---------- ---------- ---------- ---------- ---------- ----KRIDGY ILSLVQRKTH S--------- ---------- ---------- ---------- ---------- ---------- ---------- ----------
Ac_1_    VDAGAEPNSA KPGSPCLPHI P-HSASCPTA S--------- ---------- ---------- ---------- ---------- ---------- ---------- ----RKMDSY ILSLVQKRIP S--------- ---------- ---------- ---------- ---------- ---------- ---------- ----------
Cpb_1    VCIGAELDSV KTGNSFLPQS S-WSVSCPSA N--------- ---------- ---------- ---------- ---------- ---------- ---------- ----KKIDGY ILSLVQKKTH S--------- ---------- ---------- ---------- ---------- ---------- ---------- ----------
Xt_1     DCIVPELDHL KDEDSFLHQS SLCSLPL-SS G--------- ---------- ---------- ---------- ---------- ---------- ---------- ----KKMDGY ILSIIQKKAH P--------- ---------- ---------- ---------- ---------- ---------- ---------- ----------
Lc_1     ISMTNEPGSV K-TECSVPQS SSWPTSNTPS N--------- ---------- ---------- ---------- ---------- ---------- ---------- ----KKLHNY IFSLVQRRAQ P--------- ---------- ---------- ---------- ---------- ---------- ---------- ----------
Lo_1     PSN--EF--L KPEQAPEPHN VSWPASYQPS N--------- ---------- ---------- ---------- ---------- ---------- ---------- ----KRLDHY ILGLLQRRAQ P--------- ---------- ---------- ---------- ---------- ---------- ---------- ----------
Dr_1     ALN--DE--Q KPEQVVGSQT SSWHASQMLP N--------- ---------- ---------- ---------- ---------- ---------- ---------- ----KKLDSY IFGLLQRRAQ P--------- ---------- ---------- ---------- ---------- ---------- ---------- ----------
On_1     ASV------- --SAPHVSQS SSWPASSSQT P--------- ---------- ---------- ---------- ---------- ---------- ---------- --SHKRLDSY IYSLLQRRAQ P--------- ---------- ---------- ---------- ---------- ---------- ---------- ----------
Cm_1     MAPQSAPADG E-SAAAIPQS SSWPTIQLST V--------- ---------- ---------- ---------- ---------- ---------- ---------- ----KRLDNY IFGLIXXXXX XXXXXXXXXX XXXXXXXXXX XXXXXXXXXX XXXXXXXXXX XXXXXXXXXX XXXXXXXXXX XXXXXXXXXX XXXXXXXXXX
Hs_3     ---------- PD--AGGA-- ---------- ---------- ---------- ---------- ---------- ---------- ---------- ---------- ---GRPLDGY ISALLRRRRR ---------- ---------- ---------- ---------- ---------- ---------- ---------- ----------
Md_3     -----PVTGP PED-AAP--- ---------- ---------- ---------- ---------- ---------- ---------- ---------- ---------- ---GRHLESY ISGLLRRRRR IRGR------ ---------- ---------- ---------- ---------- ---------- ---------- ----------
Ac_3     Y----PPD-- QDGFAFY-D- -------EAA S--------- ---------- ---------- ---------- ---------- ---------- ---------- --HCHKVESY ISRLIRRRSQ L--------- ---------- ---------- ---------- ---------- ---------- ---------- ----------
Cpb_3    YGA--EQAGG LEEF-GGQE- --LPCYPEAA ---------- ---------- ---------- ---------- ---------- ---------- ---------- --HCHKVESY ISRLIRRRSQ L--------- ---------- ---------- ---------- ---------- ---------- ---------- ----------
Xt_3     -----PPPP- -----PPPS- ---------- ---------- ---------- ---------- ---------- ---------- ---------- ---------- ----RRAEGY ILSLL-RR-Q L--------- ---------- ---------- ---------- ---------- ---------- ---------- ----------
Lc_3     YTA--DFDG- -EVSCSATSH GEFQPYTEST ---------- ---------- ---------- ---------- ---------- ---------- ---------- --HSRKVENY IMGLIQKRNY L--------- ---------- ---------- ---------- ---------- ---------- ---------- ----------
Lo_3     -----GDQPT SEDIQQAH-- ---------- ---------- ---------- ---------- ---------- ---------- ---------- ---------- -----RVENY ILGLIQRRAL P--------- ---------- ---------- ---------- ---------- ---------- ---------- ----------
Dr_3a    PVGKIDDGPT EEDIQQAM-- ---------- ---------- ---------- ---------- ---------- ---------- ---------- ---------- -----RVETY ILGLLQRR-Q L--------- ---------- ---------- ---------- ---------- ---------- ---------- ----------
Tr_3a    -----EDLTT EEHYQQAM-- ---------- ---------- ---------- ---------- ---------- ---------- ---------- ---------- -----RVEGF IVGLIQRYAL A--------- ---------- ---------- ---------- ---------- ---------- ---------- ----------
On_3a    -----EDQVT EEDYQLAL-- ---------- ---------- ---------- ---------- ---------- ---------- ---------- ---------- -----KVEGY ILSLIQRHTL T--------- ---------- ---------- ---------- ---------- ---------- ---------- ----------
Dr_3b    -----DEKPT VDDYRQAQ-- ---------- ---------- ---------- ---------- ---------- ---------- ---------- ---------- -----HVETY ILGLIQRRSP P--------- ---------- ---------- ---------- ---------- ---------- ---------- ----------
Tr_3b    --DIREIQLD QEDYHQAH-- ---------- ---------- ---------- ---------- ---------- ---------- ---------- ---------- -----KIETY IFGLIQRRTQ H--------- ---------- ---------- ---------- ---------- ---------- ---------- ----------
On_3b    --KLKEVDPS LYDYQQTQ-- ---------- ---------- ---------- ---------- ---------- ---------- ---------- ---------- -----KVETY IFGVIQRRTL P--------- ---------- ---------- ---------- ---------- ---------- ---------- ----------
Cm_3     YKMELEGGGF YCDALEPGVG ---------- ---------- ---------- ---------- ---------- ---------- ---------- ---------- ---AKRVESY IAGLVHKRTQ P--------- ---------- ---------- ---------- ---------- ---------- ---------- ----------
Pm_A     RRG-DG-RRF KSPGKARP-- ---------- ---------- ---------- --------IS TTTSINGADR HLRHGKAQQS GAA------- ---------- ---SARSDAA LPIAKQQ--P PTQEATR--- ---------- AAPCDDGAGD DRRPGGSGAA EAVEGSLKEP RAHVTDAMKV -GCLAAPLPP LPPPPPPPP-
Lj_A     RRG-DG-RRF KSPGKARP-- ---------- ---------- ---------- --------IS TTASINGADR HLRHSKTQQS GAA------- ---------- ---SVRGDAA LPITKQQQQP TAQEATR--- ---------- TAPCDNGAGD DRRPGGSGAA EAVEGSLKEP RAHVADTMKV -GCLAAPLPP LLPPPPPPP-
Pm_B     RRPCSGFVPS PGPVPLSRDG VA-------- ---------- ---------- ---------- ---------- ---------- ---------- ---------- ---NERVSGY ICSLLDRKRA PGAS------ ---------- ---------- ---------- ---------- ---------- ---------- ----------
Lj_B     QRPCSGFVPS PGPVPLSRDG VA-------- ---------- ---------- ---------- ---------- ---------- ---------- ---------- ---NERVSGY ICSLLDRKRV PGAS------ ---------- ---------- ---------- ---------- ---------- ---------- ----------
Lj_C     PTGGRT-RRH RRHDDAGVDD EG-------- ---------- ---------- ---------- ---------- -------SAT NAA------- ---------- PPSADKVTAY IAALLKRRGQ KTRRRTSGAG T-------AA AAVVERKASN AAKTSASPSA EAARGRPITN LSVRYKHGAA AAASPVPAAT AYGGRAGGAA
Lj_D     ALDTSGTPPT PRASTDSGAA GALRTSGASG GSGTSGKSGR SGMTSGISSR SSTSGEAGTS SGTSSGTSSG GACTTGESAA PAATSTDGEA APRSAPFAER LPSAGKPDDY IARLLERRKW RQAAVAAVKA PALASSKAAA AASFSSSLSS SSSSSGRGSS VPSLPAAANA AAGEDSET-- ---------- ----------
Pm_D     ALDTSGTSGT PPTPRASADS AV-------- ---------- -GMTSGISSR SSTSGEAGTS -GTSSGTSSG RACTTGESAV PAATSTDGGA APRSPPFAER FPSAGKPDDY IARLLERRKW RQAA-AA-KA PALASSK--A AASFSSSLSS SSSSSGRAP- ---------- ---------- ---------- ----------
Hs_2     GLNTIQT-GP VLEAGP---- ---------- ---------- ---------- ---------- ---------- ---------- ---------- ---------- ----ARARAY IDRLLHL-WG RETP------ ---------- ---------- ---------- ---------- ---------- ---------- ----------
Md_2     SGPSLIRTKP IVEVP----- ---------- ---------- ---------- ---------- ---------- ---------- ---------- ---------- -----PPGGY IDKLLQLTRD RGKY------ ---------- ---------- ---------- ---------- ---------- ---------- ----------
Gg_2     TGPTLIRTRP TTEAK----- ---------- ---------- ---------- ---------- ---------- ---------- ---------- ---------- ------PGGY INKLLQLTRC KGNN------ ---------- ---------- ---------- ---------- ---------- ---------- ----------
Ac_2     VGPSLIRTRP ARESR----- ---------- ---------- ---------- ---------- ---------- ---------- ---------- ---------- ------PGGY INKLLQLTRC RGNN------ ---------- ---------- ---------- ---------- ---------- ---------- ----------
Cpb_2    TGPSLIRTRP VIEAR----- ---------- ---------- ---------- ---------- ---------- ---------- ---------- ---------- ------PGGY INKLLQLTRC KGNN------ ---------- ---------- ---------- ---------- ---------- ---------- ----------
Lc_2     TASKLIVATP LFETR----- ---------- ---------- ---------- ---------- ---------- ---------- ---------- ---------- ------PSGY INKLLQLNKC KVNK------ ---------- ---------- ---------- ---------- ---------- ---------- ----------
Lo_2     -SCVSSKTS- -FESR----- ---------- ---------- ---------- ---------- ---------- ---------- ---------- ---------- ------PDGY IYKLLQHKKN RETL------ ---------- ---------- ---------- ---------- ---------- ---------- ----------
Dr_2     -EETQNLNEE STISS----- ---------- ---------- ---------- ---------- ---------- ---------- ---------- ---------- ------SVGY INKLLQRSSS RVNL------ ---------- ---------- ---------- ---------- ---------- ---------- ----------
Tr_2     -SDTLQKAQM GYETM----- ---------- ---------- ---------- ---------- ---------- ---------- ---------- ---------- ----TQT--Y IDRLLVRSFN K--V------ ---------- ---------- ---------- ---------- ---------- ---------- ----------
On_2     ---SYNTLEK AYETK----- ---------- ---------- ---------- ---------- ---------- ---------- ---------- ---------- ----TLTH-Y IDNLLQRSLS K--I------ ---------- ---------- ---------- ---------- ---------- ---------- ----------
Cm_2     ----SNRKIL ASESL----- ---------- ---------- ---------- ---------- ---------- ---------- ---------- ---------- ----TSTTPF NPVMSWPPVL ETTF------ ---------- ---------- ---------- ---------- ---------- ---------- ----------
Ac_4     ----DEPPLE AILDEPPKGV GSRSQEQR-- ---------- ---------- ---------- ---------- ---------- ---------- ---------- ---TVQLDQY IAKLVLRYKC RSIA------ ---------- ---------- ---------- ---------- ---------- ---------- ----------
Cpb_4    ---SERAAIH TVQEGSPQSG TAPSEEQR-- ---------- ---------- ---------- ---------- ---------- ---------- ---------- ---QVQLDRY IAKLVLRYRC RSAA------ ---------- ---------- ---------- ---------- ---------- ---------- ----------
Lc_4     ---QEDGLKP PAEAKSLSEN TLKRHEVLN- ---------- ---------- ---------- ---------- ---------- ---------- ---------- ---RTKLDKY ISKLVLRYKC RSAA------ ---------- ---------- ---------- ---------- ---------- ---------- ----------
Lo_4     ----EGGGGE EVPRCPCGPS PPDPPGPA-- ---------- ---------- ---------- ---------- ---------- ---------- ---------- -----RADRY IGQLLRRYRR RP-------- ---------- ---------- ---------- ---------- ---------- ---------- ----------
Dr_4     ----SPETEQ LVESELEENP VKIQPPAS-- -PSF------ ---------- ---------- ---------- ---------- ---------- ---------- ----TQLEQY INRLVHQHHC RVAA------ ---------- ---------- ---------- ---------- ---------- ---------- ----------
Tr_4     ----EVAQSD EILEPIVAPP LHVQSQQP-- -PSF------ ---------- ---------- ---------- ---------- ---------- ---------- ----THLEQY ITRLARQYHS RV-------- ---------- ---------- ---------- ---------- ---------- ---------- ----------
On_4     ----EGPQHD EPTESNADTA LPLQPQQPPT PPSF------ ---------- ---------- ---------- ---------- ---------- ---------- ----TQLEQY ISRLARQYHS RA-------- ---------- ---------- ---------- ---------- ---------- ---------- ----------
Cm_4     ----ETNLRP SPGPNSQAPG PSLSVETR-- ---------- ---------- ---------- ---------- ---------- ---------- ---------- ----GRLDGY ISSLASRRAG R--------- ---------- ---------- ---------- ---------- ---------- ---------- ----------
Lo_4r    SSR-HSSLGS AD-LQPNATT CCTPSSEFCS PPAS------ ---------- ---------- ---------- ---------- -------ASD SRYSSQLPIP FSEQRRLEKF ISKLVTRWHP ---------- ---------- ---------- ---------- ---------- ---------- ---------- ----------
Dr_4r    SIGLVCPWNF SEHSCRLDKS TYLSSSLLRK HSER------ ---------- ---------- ---------- ---------- ---------- --------SN HFGKQGLEEV ILKRARVQ-- ---------- ---------- ---------- ---------- ---------- ---------- ---------- ----------
Bfl      -CTDV----- RSDHN---QS SSRPAVVTDA QAVSLTGVPA DDKF------ ---------- ---------- ---------- ---------- ---------- --AKVPPGGT SAGLLRPVST PPDISRVWNC EVLHTKNSEQ TTEQLVDIPD YENIFSAYSE LPGNDVAPRR HSFAGSRAQK QHVEDAIPNY ENLSNLDNDY
Motifs   ---------- ---------- ---------- ---------- ---------- ---------- ---------- ---------- ---------- ---------- ----****mo tif5a***-- ---------- ---------- ---------- ---------- ---------- ---------- ---------- ----------

                810        820        830        840        850        860        870        880        890        900        910        920        930        940        950        960        970        980        990       1000

Hs_1     ---------- ---------- ---------- ---------- ---------- -----VRTNK PRTSVNA--- -------DPT KGLL--RN-- ----GSVCVR APGGVSQG-N SVNLKNSKQA -----CLPSG G-IPSLNNGT FSPPKQWSKE SKAEQAES-- -------KRV -PLPEGCPSG AASDLQSKHL P----KT-AK
Md_1_    ---------- ---------- ---------- ---------- ---------- -----VRTNK LRTSVNA--- -------DPT KGIL--RH-- ----GSVCVR ATGGVTHS-N SVNLKNSKQA -----CLHSG G-TTSLENGT FPPLKQWLKE SKGEQLES-- -------RRM -PVTVGCLPN AATEHQSKHL P----KN-VK
Gg_1     ---------- ---------- ---------- ---------- ---------- -----VRTNK PRTSLNA--- -------DPT KGIL--RH-- ----GSMCVR QTAGVVSHSS VVNLKSAKQV -----SLPSS G-ATASDNTA PSPLKQRPRE AGGEQVES-- -------RKV -LLPAAFPP- --SELQSKHQ P----RG-AK
Ac_1_    ---------- ---------- ---------- ---------- ---------- -----VRTNK PRTNLNA--- -------DPT KGIL--RH-- ----GSMCLR QVPGTALH-N ATTLKNLGQV -----FPPSG GTTAAIENGA FSPLKQHTKE T--EHQET-- -------KKM -PPT-----N ANNELQSKPP T----RN-VK
Cpb_1    ---------- ---------- ---------- ---------- ---------- -----VRTNK PRTSLNA--- -------DPT KGIL--RH-- ----GSMCVR QTAAVSHT-N IVNLKNSKQA -----CLHSS G-TTTLDNGT FSSLNQPSKE SNGEQLES-- -------RKV -PTAAAYPPS AVNELQSKHL S----RN-VK
Xt_1     ---------- ---------- ---------- ---------- ---------- -----VRTNK PRTSVNA--- -------DPG KGIL--RH-- ----GSICVK QTGGVSQS-S TVNLKNSKQT -----CLHST G-MISVDNST YPSLKQCSKE SLSEQLES-- -------KRM -PSISTYPSC NVNELQSQNN S----RNTVK
Lc_1     ---------- ---------- ---------- ---------- ---------- -----IRTNK LRTSLNT--- -------DPV KGIL--RQ-- ----GSICVK QAVGVSQGS- --TAENSRQA -----CLQSH G-TNSLDSSA LPPRKQLPKE SNNEQLEN-- -------KTE -HMYATYQQN SSNEYQSASL L----KKKDK
Lo_1     ---------- ---------- ---------- ---------- ---------- -----VRTNK PRTSINT--- -------DPA KSIL--RQ-- ----GSICVR QT-VGAAQGK TPDLKASRQA -----CLHSS A-TGSADVSA ASPMKQWPAE ATIDQLEN-- ---GKSLVPS AYHQHSEHLS NTIEIHTNSL S----KKNTS
Dr_1     ---------- ---------- ---------- ---------- ---------- -----LRTNK PRTSINT--- -------DPS KSIL--RQ-- ----ASLCSR APASVQAQQW TSDLKPNWQT -----CLQDA S-ATSTEPST ASPQRQWSAE -SKGGTPQ-- -----NGAYL SSSQPQNSYS TTNE-NTNCL L----KKKVS
On_1     ---------- ---------- ---------- ---------- ---------- -----IRTSK PRTSIST--- -------DAL KSIL--RQ-- ----ASLCVR QVSGPGSGFG --TLSGSEFK ASWTARRTSA EGA-----GT SSSQRQWSVD GKLEEQET-- -----QSVLK YD-VQSSCSA NNNDSSTNSL R----KKSK-
Cm_1     XXXXXXXXXX XXXXXXXXXX XXXXXXXXXX XXXXXXXXXX XXXXXXXXXX XXXXXXXXXX XXXXXXXXXX XXXXXXXXXX XXXXXXXXXX XXXXXXXXXX XXXXXXXXXX XXXXXXXXXX XXXXXXXXXX XXXXXXXXXX XXXXXXXXXX XXXXXXXXXX XXXXXXXXXX XXXXXXXXXX XXXXXXXXXX XXXXXXXXXX
Hs_3     ---------- ---------- ---------- ---------- ---------- -----RGAGQ PRTSPGGA-- -------DGG P----RRQ-- ----NSVRQR PPDASPSPGS ARPAREPSLE RVGGHPTSPA ALS-----RA WASSWESE-A APEPAAPPAA PSPPDSPAEG R--------- ---------- ----------
Md_3     ---------- ---------- ---------- ---------- ---------- -----PGPGQ PRTSPGPG-- -------PAE PSAALRRQ-- ----NSARQR PEEPAHSGGG GSPGPGG--- ---------- ---------- -WPPWDSSPP STEPVPAAAG SSPP-SPGEG R--------- ---------- ----------
Ac_3     ---------- ---------- ---------- ---------- ---------- -----VRGSK PRTSLGS--- -------EPN V---V-RQ-- ----NSLCKR PLELLPPPQS ERKHPPS-VE RGSSTPSPCG HEGGTAAAAR IWSSWDAAA- ERTLSTKV-D DYSPPPPSS- R--------- ---------- ----------
Cpb_3    ---------- ---------- ---------- ---------- ---------- -----ARGGK PRTSLNA--- -------EPP AKAVA-RQ-- ----NSVCKR PSELLAPQV- DRKHPPA-ME RGSSTPSPCG YEGGAGAAAR IWSSWDAAA- ERTLAGKGAP EGYAPPHSS- ---------- ---------- ----------
Xt_3     ---------- ---------- ---------- ---------- ---------- ------RPPY LR---SA--- -------SAE ---------- -------RPP PPPPAPPPSA SGCR------ ---------- ---------- ---------- ---------- ---------- ---------- ---------- ----------
Lc_3     ---------- ---------- ---------- ---------- ---------- -----VRSSK PRTSLNS--- -------EPL K-GLA-RQ-- ----NSLCIR HSEVL-GTQS NNCELKYPQS ERAN-ISSPY SYEGNGLSGK CRPQWNPGT- EKTQANKNIQ NTQTG--ETY TML------- ---------- ----------
Lo_3     ---------- ---------- ---------- ---------- ---------- -----LRPSK PRTNLNS--- -------EPH K-GLA-RQ-- ----NSLCRR PLEPG-YHQG GHGAIS-PSY EGPT------ ---------- ----WGCR-- ---------- SLDED--GYT GMP------- ---------- ----------
Dr_3a    ---------- ---------- ---------- ---------- ---------- -------SST ---------- ---------- ---------- ---------- ---------- ---AGLNSDP SEWQ-ELQTY PPGYPQLNQS E---WQEW-- EENE------ GQSQD--GYY FNL------- ---------- ----------
Tr_3a    ---------- ---------- ---------- ---------- ---------- -----SRPCQ PRTTLIP--- -------DTP GHNSI-HR-- ----RTPCLF TENLLLD--- --PVLLS--- --QS-DLSTN PTNQK----- ----WGCDLL EEEACGGEAP TLEGD--SYL SLP------- ---------- ----------
On_3a    ---------- ---------- ---------- ---------- ---------- -----PRPCQ PRTTFSP--- -------DPP YCSAS-GH-- ----SSLHRR TPSLTTE--- --HRPSDHHL QPHG-DLSTN PNGQG----- ----WGCDL- EREACGGEAP SLEED--CYL ALP------- ---------- ----------
Dr_3b    ---------- ---------- ---------- ---------- ---------- -----ARPNK PRTSLSP--- -------EPR A--VI-RQ-- ----SSLSRK EP-------- --PFVQE--- ---------- ----RLA--- ---WA----- ---------- --EDE--CTG -VR------- ---------- ----------
Tr_3b    ---------- ---------- ---------- ---------- ---------- -----TRPSK PRTSLAH--- -------DAR AVGVA-RQ-- ----SSLCHK DEQMPPK--- -PASAPEISG VSQT-SDAYF PKAYNT-DQE GYQGMGPAED PAPPCRHQQQ QQHPPGPYH- -RP------- ---------- ----------
On_3b    ---------- ---------- ---------- ---------- ---------- -----TRPSK PRTSLAH--- -------DAR VVNVV-RQ-- ----SSLCQK DEQHLPK--- -QESSQEIIA PSQW-SEGIA SEAGHNLDQE QYLRIGPTED PPLPYHLQQK GLY--T---- -KP------- ---------- ----------
Cm_3     ---------- ---------- ---------- ---------- ---------- -----ARSCK LRTSLNS--- -------DSN PKGVL-RQ-- ----SSLCLR NVDASSP--- -RGSVCESLS PPPP-SQPGL PPFEDNLPSS STTSSSSSRH RSPWNSGLAK KAHRP--EAY SA-------- ---------- ----------
Pm_A     ---------- ---------- ---------- ---------- ---------- ---------- ---------- ---------- ---------- ---------- ---------- ---------- ---------- ---------- ---------- ---------- ---------- ---------- ---------- ----------
Lj_A     ---------- ---------- ---------- ---------- ---------- ---------- ---------- ---------- ---------- ---------- ---------- ---------- ---------- ---------- ---------- ---------- ---------- ---------- ---------- ----------
Pm_B     ---------- ---------- ---------- ---GGNGVKL SRMESFNAGP NMPITNGRLK PKHEEKPVTL ENINLGAD-- -LKPDTLQ-- ---CVDSCKE VIPEKNVQTI NESETPRC-- ---HQQRS-E PSPSPKSKEQ PKLIANVLPL --------IC NDSPKRTGGL PSHLSRVKGS SRVERYEEDS KP--------
Lj_B     ---------- ---------- ---------- ---GGTGAKL SRMESFNAGP NMPITNGRLK PKHEEKAVTP ENINLGAD-- -PKPDTLQ-- ---GVDSCKE VIPEKNVQTI NESEAPRC-- ---HQQRS-E PSPSPKSKEQ TKLIANVLPL --------RC NDSPKRTGGL PSHLSRVKGS SRVERYEEDS KP--------
Lj_C     GSPV------ ---------- ---------- ---GGSSVNL AR------SP RGGTLPALVR PASIAHFDTS RYHDDDEDDD -GGGDDDERR WRASGPHVHP GAGDHRSSSE KSPNLQQQQS RQQHQQQQQS TDRERECRRR ALASVSMDLR PQRGAGWWGA AAEPTVGALG WQQGELGRPQ YQQR------ ----------
Lj_D     ---------- ---------- ---------- ---------- ---------- ---SVRLSLR SPPLPPPPPP PLLLPPVLPT ASPLPSSSSS SSSKAS---- ---------- ------VPPP PPAAPPPLPL PSPPPSPSPP GRRATRLVAS VPPMKHGIVR GVSQFLTGGR LHAGKRAANK QQPQPQQQRQ PQPPPPPSKA
Pm_D     ---------- ---------- ---------- ---------- ---------- ---PCRLS-R PRRTPPPDTS PRXXXXXXXX XXXXXXXXXX XXXXXXXXXX XXXXXXXXXX XXXXXXXXXX XXXXXXXXXX XXXXXXXXXX XXXXXXXXXX XXXXXXXXXX XXXXXXXXXX XXXXXXXXXX XXXXXXXXXX XXXXXXXXXX
Hs_2     ---------- ---------- ---------- ---------- ---------- -----AKGSE GEQGPLRHAA SPSPQRQ--- ---GGWSTD- --GGGRL-LV FAPGREDE-- -GG-----PA QSR-----GA GRGG--PQQQ GY-MPLEG-- -PQQSGSLPE EGSKPSNS-C VL-------- --RETMVQAS P---------
Md_2     ---------- ---------- ---------- ---------- ---------- -----TGGSV SERGSVKGSL PPSQQRLSIT SGISGLRGN- --SDSQL-EK QVSSPESRQA KVG-----RS QR-------E VPDANSMKLQ EAREACLDTE QPASQQS-LE SM---ATNTS FP-------- --EEVSPASA VQEEPGGI--
Gg_2     ---------- ---------- ---------- ---------- ---------- -----R-ADA NERISTKSQP ATVHQRLIIT PSAGGVKIN- -SSGSQL-EK QASSLESNKA EG------KL QR-------E LSEGECAKLQ EA-MHCVNEE QPSGF---PD TEPS-AVNSC YPAKSAARGS PLAEATESSL EH--------
Ac_2     ---------- ---------- ---------- ---------- ---------- -----Q-ADA SERCPTKSQP FTMLQRLIII PSAGGVKIN- -SSGSQL-EK QESSPHRNKA ER------KL QR-------E MPEGECAKQQ ET-LDCVNVE QTATM---GH AESSLAVNAS HSAKSSVRDS PVTEIVESSL ER--------
Cpb_2    ---------- ---------- ---------- ---------- ---------- -----H-ADA SERVSTKSQP STMPQRLIIT PSTGGVKIS- SSSSNQL-EK QGNSLEGNKA EG------KL QR-------E VPEGECAKQQ GT-VHCVNVE QPSNL---PD PESS-AVNSC YPAKSTARGS PLAEAVGRGL EH--------
Lc_2     ---------- ---------- ---------- ---------- ---------- -----Q-PDV NIRGFEKNQP TVTLQKDTPI QNIGPVKIN- -NDRQSS-MQ NEST-RNSRD VG------KI PA-------V PAKNEKAKQQ G--ANYLGNS TPSSN---FR EEQKI-TSDC CPLKSNARGL ASVEDV---- -RNYTGKS--
Lo_2     ---------- ---------- ---------- ---------- ---------- -----Q-PEG CCQV-DRAQP LLSCHNGVFS LSTAVTKVN- -CRAMLK-SQ GDQSLSQGQP NI------KQ QEVK-TVSEE KVAIVNYRQE TV--NCAQTA -KVFH---HR ED--L-KACH SPLDADQRVS VLVEAN---- -KCPERVQ--
Dr_2     ---------- ---------- ---------- ---------- ---------- -----LSSIK RIETVSGTHE ---QIPKSQE MIYGSLNGP- -QLLGSL-QQ LTMPLENALE TGKTSALNNN QKQM-----D PPCLGSNFKE VE--VQMLHH GKHP------ -ESSL----- ----DLQKNN FPINNT---A GKVGA-----
Tr_2     ---------- ---------- ---------- ---------- ---------- -----K-TDT GPEDVQTHSE C---QRNLAK VVTVVPEVP- -QRDKSV-VQ PHSVQTTSS- SLD-----NS QKRN------ --CMTYSSQE RA--DNANVK QPVKVP---- -DTS--FRYS YPARS--RDC TTEESTISSM RKKDNS----
On_2     ---------- ---------- ---------- ---------- ---------- -----Q-SDA GSQTLEKHSD YC--RKPIED KSVFS-EVA- -QKEVLM-LQ PLPTKMTNII PLD-----FT EKRH------ --CMAYSSQE LI--DIADHK QSVRSP---- -QVP--QQYS YPAAM--KEY SSDEVTYSSL KRNDKP----
Cm_2     ---------- ---------- ---------- ---------- ---------- -----G-HKP KLSQQSTAGE I-RTTAVHGD FELNQQTAA- -QKSDIS-QS FNGMKNNSNH KAMVQTVAEQ QDSN------ ---DGHYKDL GL--S-AYVA V--RLQ---- -EAG-HGSIK QLRED--SEA SI--GVQIVS KLDMKD----
Ac_4     ---------- ---------- ---------- ---------- ---------- -----S---- -RTD------ -------SGS LAAYQ-KS-- -LSMSSVC-- --------SS V--LG----- ---------- ---------- ---------- ---------- ---------- ---------- ---------- ----------
Cpb_4    ---------- ---------- ---------- ---------- ---------- -----S---- -RTE------ -------PGY LAGHQ-KS-- -QSMSSVC-- ---------- G-S------- ---------- ---------- ---------- ---------- ---------- ---------- ---------- ----------
Lc_4     ---------- ---------- ---------- ---------- ---------- -----SHIVM RRSE------ -------LSH LGGHQ-KS-- -LSMSSIC-- ----SSNSSV G--------- ---------- ---------- ---------- ---------- ---------- ---------- ---------- ----------
Lo_4     ---------- ---------- ---------- ---------- ---------- -----GSVLL SALD------ -------PGP AGRWQAKS-- -LSLSSVC-- -SGSTSSTLT GGALPSVPSL GSLGSSGSTP QAVWGGRR-- WVPSPAEDEE GRS----DRG GSLR------ -------GLG GDGLY----- -----RGKHY
Dr_4     ---------- ---------- ---------- ---------- ---------- ------EASS R--GPQINIA G------TGY EGLHP-RS-- -ASSSSLS-- ---------- -GGSM-TPCK SILGNS-ARV SLSSIGKRAS RNSINLGNLP SVTGE--DFN ISFHL----N LNLNLNPNLN KTLS-VDA-K NVKEEVSGSC
Tr_4     ---------- ---------- ---------- ---------- ---------- -----SSSTS DLSAGAT-AP V------THR GFSSQGKNH- -GSTQSLSAF ESRNTFS--- ----I-TPCK SLLGNS-ARV SLSAAGKKAT RNSINLGNLP SATGE--DLN INLHL----N LNLNLSPGSI SAQAALDNPK K-------ES
On_4     ---------- ---------- ---------- ---------- ---------- -----K-STS DLTSPATPGA V------THR GLCTPGKTH- -GSTQSLSAF ESRSTPSTQP -GVNI-TPSK SLLGNS-ARV SLSTTGKKGT RNSINLGNLP SATGE--DLN INLHL----N LNLNLTPGLN SGHGLVDNPR N---------
Cm_4     ---------- ---------- ---------- ---------- ---------- -----AGAGG E-----AGAG S------RLL GSQPKCRS-- ---LSSICG- ---------- ---------- ---------- ---------- ---------- ---------- ---------- ---------- ---------- ----------
Lo_4r    ---------- ---------- ---------- ---------- ---------- ---------- ---CSEASRL I------ASV SELQQLRS-- ----SSLSSV A--CQNSCGE GASLS-SGSS YSICGV-QTL RMKKRRHSAF AYVNDDSRQS GLFIS--DVP RKRAD----L EEGTRLDGSA GHWNPNSGCK AELRGSKHLC
Dr_4r    ---------- ---------- ---------- ---------- ---------- -----CNNSG D---RVKMSS R-PKKGMHCG NPIHKSKSFC AFQHTSL--- -----QKNRE AQ-QY-TRSY LNIXXXXXXX XXXXXXXXXX XXXXXXXXXX XXXXXXXXXX XXXXXXXXXX XXXXXXXXXX XXXXXXXXXX XXXXXXXXXX
Bfl      GDYVTLTPED LPQIPRATTA RPTSEAYSKS PSDSGVKPVP ATSENASRPE VQQERKAPRK PKPTP----- ---------- ------RQ-- ----SLLKKV NPTANPP--- -LPQPRAEPQ VDTLNVNVVK ATPVSETPSN QTTGQENATS KTAQPGPALL MA-----SLI ADG------- ---------- ----------
Motifs   ---------- ---------- ---------- ---------- ---------- -----***** **5b*----- ---------- ********** motif5c*** ---------- ---------- ---------- ---------- ---------- ---------- ---------- ---------- ---------- ----------

               1010       1020       1030       1040       1050       1060       1070       1080       1090       1100       1110       1120       1130       1140       1150       1160       1170       1180       1190       1200

Hs_1     --PASQEH-- ARCSAIGTGE SPKE------ -------SAQ LSGASPKESP SRGPAP-PQE N--KVV---- -QPL-----K KMSQKNSLQG VPPA-TPPLL STAFPV---- ---------- -EERPALDFK S-EGSS-QSL EEA------- -HLVK----- AQFIPGQQPS VRLHRGH--R NMGVVK--NS SL-KHRGPAL
Md_1_    --PGSQEF-- AWCPVSGAGD SPKE------ -------SGQ LFTASPKESP GRDPAL-QQE N--KVV---- -QPL-----K KMSQKSSSQA ALPAIPPAVP PPPLPSSQPI ---------- -EERPALDFK S-EGSSSQSL EDG------- -LLVN----- AQFIPGQQQS SKLQRAN--K NVRILK--SS TL-KHRAHGL
Gg_1     --AVPPEL-- SRHAVAATGD VPKE------ -------GSQ HFAASPKESP GKPVVL-QPE T--RVS---- -QPP-----K KVLLKGSSQA AHSSSPPV-- ---------- ---------- -EERPALDFK S-EGSSSQSL DDG------- -LLVN----- AQYIPAQQQS VKLHKGT--R NVKILK--SS AL-KHRPHLA
Ac_1_    ----SQEV-- ARCPVVGKGD MGKE------ -------SGQ SIVACPKESP CRHGTL-PKD N--KAT---- -HVP-----K KILFKNSGVQ VSCSIPLTQ- ---------- ---------- -ETRLPLDFK S-EGSSSQSL EEA------- -ALVN----- GSYIPAEPQT SRLHKGS--R SVKILK--SS MA-KHRSHLV
Cpb_1    --PASQEL-- NWSPAATTGD IPKE------ -------NGQ LFATSPKESP GRHTAL-QQE N--KVT---- -QPP-----K KLLLKNRLQA AHPTSLPL-- ---------- ---------- -EERPTLDFK S-EGSSSQSL DEG------- -LLVS----- AQFIPAQQQS LKLHKGT--K SVKILK--SS TL-KHRSHLA
Xt_1     --SVCQGL-- ARGSVAMTSN VQKENVTPNA PTNLSNASSS ACNGSPREST QMSALL-PQE I--KVV---- -PPV-----K KISPKNTLLS YHASSSF--- ---------- ---------- -DERPPLDFK S-EGSSSQSL DEG------- -LLVN----- AHYIPAQQQG VKLHKNI--K NVKIVK--SS TL-KHRANVQ
Lc_1     --VLSQGL-- PLSPV---SI DYQD------ ------GNTP YVINAPNDNM HKSAVV-MHE D--KVM-VNQ -PVNAVS-PK K-GLNSSPAT L--------- ---------- ---------- -EDRPPLELR S-EGSSLQSL DEG------- -QLVN----- AQYIPAQQQI LKLHKGT--K NVKTVKVRSF TV-KHRINID
Lo_1     --VANKGL-- PLSTV---TK DCQE------ ------MGAP HSTSSPKDS- GNRYLP-MPD D--RKG-SQS -PRQ----SL K-SPPAVPPP L--------- ---------- ---------- -EKRPPLELR S-LGSSSQSQ DEGG------ -RLVS----- AQYIPAQRQA GKLRKGG--R NVKIVKVKGA AL-KTRANI-
Dr_1     --GTSKGQ-- LLSAT---PK DCPD------ ------LGSP KAVSSPKLNK HCFY---NVE DN-KTG--QA -MKASP--LK R-SPKTQTSL SCGKD----- ---------- ---------- -SEQLAQELV S-LGSSSQSQ DEGG------ -PLVS----- AQYIPAQKQN VNLQKGGT-K NIKIGKVKNS ASSKSRPQVF
On_1     --ADLPHA-E LSAKTL--PK NFRD------ ------LGSP KANPAPKEIK PPCCPP-DQE MFLKSAPV-- -MKTQSSTPR N-SPGT---- ---------- ---------- -GRSA-LELA S-AGSSSQSQ DEAAGEGGSC -HTVT----- NRYVPAHQHK L--RKGG--- -FKIVKVKSS SM-KTNRVS-
Cm_1     XXXXXXXXXX XXXXXXXXXX XXXXXXXXXX XXXXXXXXXX XXXXXXXXXX XXXXXXXXXX XXXXXXXXXX XXXXXXXXXX XXXXXXXXXX XXXXXXXXXX XXXXXXXXXX XXXXXXXXXX XXXXXXXXXX XXXXXXXXXL EDSG------ -HLVN----- AQYIPGQQQV IKSRKGT--K NVKIGKVKSS AL-KPKPHC-
Hs_3     ---------- ---------- ---------- ---------- ---------- ---------- ---------- ---------- ---------- ---------- ---------- ---------- ---------- ---------- ---------- --LVK----- AQYIPG-AQA ATRGLPG--R AAR-RKPP-- PLTRGRSV--
Md_3     ---------- ---------- ---------- ---------- ---------- ---------- ---------- ---------- ---------- ---------- ---------- ---------- ---------- ---------- ---------- --LVK----- AQYIPA-AHG AVRGPPVA-R AARRKGPPPP PLTRGRSV--
Ac_3     ---------- ---------- ---------- ---------- ---LKK-PPK LQALAHMRSP SA-DQYEA-- ------VYPS SPL------- ---------- ---------- ---------- -PDRE--STN VQ---ASYDA EEGACL---- --MVK----- AQYIPA-QQP SRVQQAHH-R PGK-KKPP-- PLTKGRSV--
Cpb_3    ---------- ---------- ---------- ---------- ---LKK-PPK LQALAHLRPA LSVDHYEE-- ------GPDE DGT-HARNGQ E--------- ---------- -------PPP YEERG----G RS---PLACT EDGACQ---- --MVK----- AQYIPA-QQP PRAQQAH--R GAK-KKPP-- PLTKGRSV--
Xt_3     ---------- ---------- ---------- ---------- ---------- ---------- ---------- ---------- ---------- ---------- ---------- ---------- ---------- ---------- ---------- --MAN----- ASYTP----- -----PH--R VPR-RKHP-- PLLKGHSV--
Lc_3     ---------- ---------- --------VQ QQRSGNDLLP PHLLKK-QPK LIANV--PPL RPTS--LE-Y YELNYQASLN SPQENHSFEH SLKEISNIAQ LGKSASPRNG HNSAEIHSPS YEEKSEVRSK RS---PPQSM DEGLCQ---- --MVN----- AQYIPA-QQP TKAQAAN--R SVR-NKS-GS -MNKNRPI--
Lo_3     ---------- ---------- ---------- LNSDESFSYI YSRPKPTP-- LAAVR----P ASLDYPCSAA LE-PTSSDQE SPE-HYQGYA M----HRS-- ---------- --------PP ADDQ------ ---------- ---------- --MVS----- AQYIPA-QQA CRAQQSS--R NSS-QRSSGK -PGKSRA---
Dr_3a    ---------- ---------- ---------- PSSQIGPTSL SS-------- ---------- ----EEPE-- ---SSLEVDQ YLV-EDVYTS SNESPRHQ-- ---------- ---------- ---------- ---------- ---------- --PVY----- TERRAT-MSD TRKPHIHT-C CNHTCVPTTS -TPVSR----
Tr_3a    ---------- ---------- ---------- YPQSRSHSLT ERLPSPLPFI -DPNS---AI ---------- --------PP SPQ-NCIHPQ LQSIN-HKPS ---------- ---------- ---------- ---------- ---------- --LAS----- SCFIPG-QAC HAPVHSSR-P CNPVRSK--- --SIQKA---
On_3a    ---------- ---------- ---------- YPQPNPISLA GRLPSPPPSL -DHNC---GV GALDLCCE-- --------PP SPH-HYLHPQ PPILHMHS-- ---------- ---------- ---------- ---------- ---------- --LVS----- AQYIPG-QAC HAPLCSPR-H YNQEQPK--- --SNQVFT--
Dr_3b    ---------- ---------- ---------- YPRMHT---S TSLDFPCGAL -DPSS---S- ---EAE---- ---------- SPQ-HFYQHT ------H--- ---------- -------SPS SDE------- ---------- ---------- --LVN----- AQYIPA-QPC RAQAR----- --PTTYK--- -SPKSSY---
Tr_3b    ---------- ---------- ---------- RQASRSTLVE YQSFRVLQVN PDSSN---S- ---EPD---- ---------L PPNYHYHSPP PLLK-AHQ-- ---------- -------PPP AEDQ------ ---------- ---------- --LVN----- AKYIPA-QPC RGSARASG-H QHSNPHKG-F GVSKPNQGAF
On_3b    ---------- ---------- ---------- RQASMM-LLD YPACKDVQ-- PDSST---T- ---EPD---- ---------- LSQ-HLPKYQ SPPAAAAKST Q--------- -------PSS PEDH------ ---------- ---------- --LVS----- AEYIPG-QPC RTSNRAYA-H HHTNHHKG-P GVSKSNRCTY
```

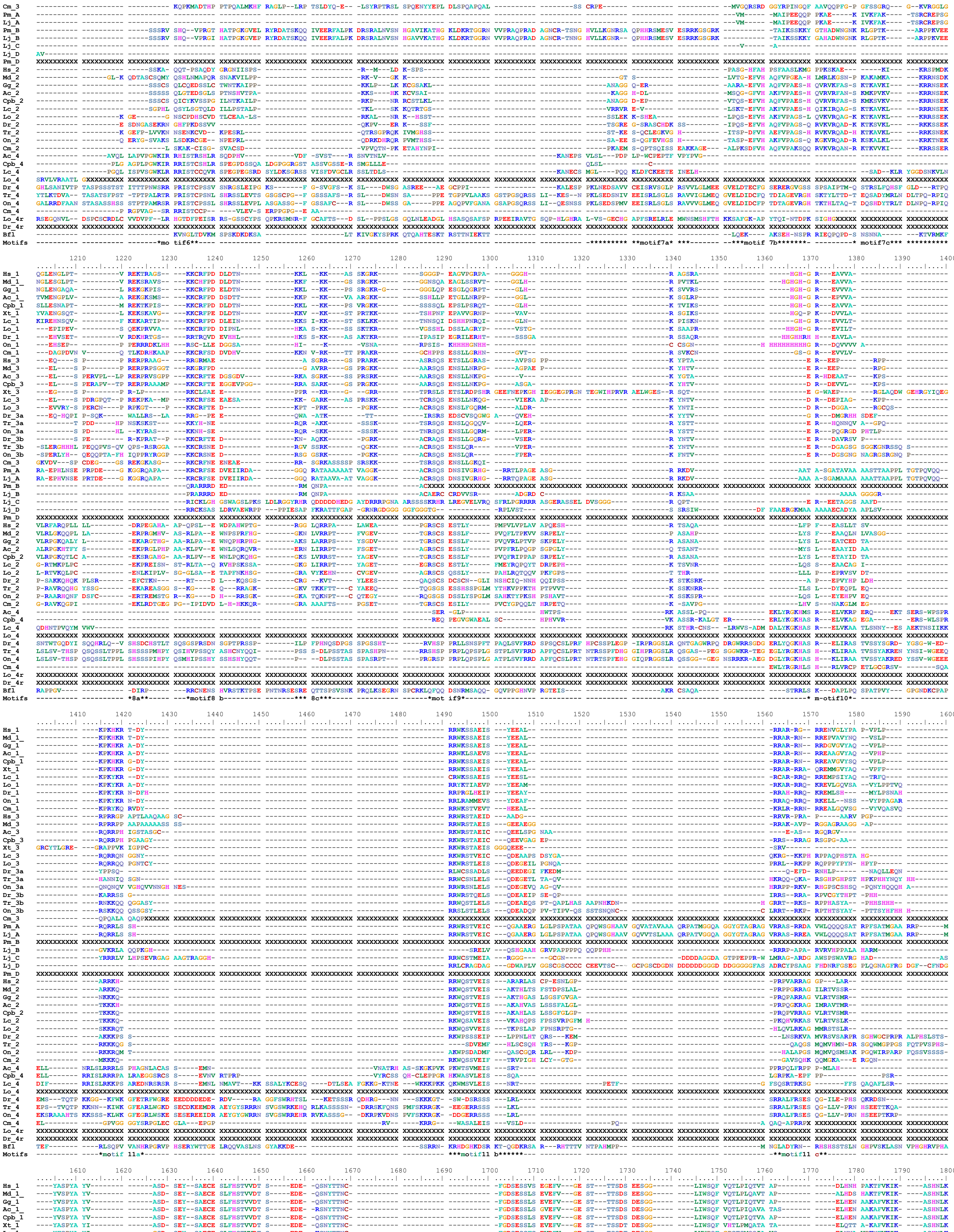

```
Dr_1     ----YTSPYA YV-------- ------GSD- SEY--SAECA SLFHSTILDT S-----EDE- -RSNYTTNC- ---------- ---------- ---------- -FGDSESSAS EADYV---AE SS---TSSDS EGSAG----- ----VNWRQI SQAGSGSH-- ---------- -----GMTSA QAKAFVKIK- ----ASHNLK
On_1     ----LTSPYS YTA------- ------GSD- SEY--SAECA SLFHSTIVDT S-----EDE- -RSNYTANC- ---------- ---------- ---------- -FGDSESS-- EEEYV---EE ST---NTSDT EESGGGG--- ----MGRGRG QLGGAGVRAT GQ-------- -----EMTSA QAKAFVKIK- ----ASHNLK
Cm_1     ----QASGVC GIR------- ------GSD- SEY--SAECE SLFHSTVADT S-----EDE- -QSNYTTNC- ---------- ---------- ---------- -FGDSESSLS EVDFV---GE TT---SSSDS DESGG----- ----LVWPQL GQPRPSQATA TTTAAP---- -----ELKEV STKAFVKIK- ----ASHNLK
Hs_3     ----GPSPSA P----QRRLL YGCA--GSD- -----S-ECS AG------RL GPLGRRG--P AGGVG--GG- ---------- ---------- ---------- -YGESESSAS EGESP---AF SS---ASSDS DGSGG----- ----LVWPQQ L-------VA ATAASGG-GA GAG---APAG PAKVFVKIK- ----ASHALK
Md_3     ----GPSP-A P----PRRLL YGCA--GSD- -----S-ECS AGRGGAGGRR APPLGSVGGS YGGSYG-PA- ---------- ---------- ---------- -YGESESSAS DAESL---AF SS---ASSDS EGSGGG---- ----LVWPQQ L-------AA ASAAAGP-AG GPGGP----Q G-KVFVKIK- ----ASHALK
Ac_3     ------TAI- ---------G YTYNT-GSD- -----S-ECP AE----PPRQ APTCRMEEG- -------LV- ---------- ---------- ---------- -YGDSESSVS DGDSP--AVY SS--GASSDT DESGG----- ----LVWPQQ LSPQL---VS AS---GP-GK TSGG-----Q P-KVFVKIK- ----ASHALK
Cpb_3    ------SP-- -----PCNVL YGYA--GSD- -----S-ECS GGRGG-GARR APVCRLEEG- -------LA- ---------- ---------- ---------- -YGDSESSLS EGGSP---TF ST--G-SSDT DESSG----- ----LVWPQQ LSPQL---VA SA---GP-GQ AGG------R P-KVFVKIK- ----ASHALK
Xt_3     ---------- ---------- ---------- ---------- ---------- ---------- ---------- ---------- ---------- ---------- ---DSEGEEE EEGE------ ---------- ---------- ----LVWPLQ LPPR------ ---------- -----DADGR P-QMF-KVK- ----ASQALK
Lc_3     ---------- -----PRSSM YGYA--ESD- SEY--SADC- ----GS-VRC APVCEPEDD- -LSSYTMNC- ---------- ---------- ---------- -FGDSESSLS EVESP---GF ST---CSSDT DESGG----- ----LVWPQQ VPPQL---VA SS-------- GSN--QSSMQ P-KVFVKIK- ----ASHALK
Lo_3     ---------- ---------- ------ETDF QER--APLCR P--------- ------EEG- ----YPTPC- ---------- ---------- ---------- -LGDSESSLS EADSP---GS SS---LSSDS DESGG----- ----LVWPQQ LPPQL---A- -----RP-PS PSS-PPCGPQ P-KAFIKIK- ----ASHALK
Dr_3a    ---------- ------QRL- ---------- QE-------R ---------- ------ME-- ----ASTGP- ---------- ---------- ---------- -TNGSDSSLS ETFSP---GT SS---VSSDS DESGG----- ----LVWPQQ LPPRL---PP SSSSS----- -------SQN PPNAVVKIK- ----ASHALK
Tr_3a    ---------- ------QRHR -------LDV QER--APLCQ ---------- ------EED- ---SYAATI- ---------- ---------- ---------- -PAESESSMS EVYSP---AS SS---LSSDS DESGG----- ----LVWPQQ LPPRF---AS TSSSSSP-SP QTTA-NATAQ P-KAFVKIK- ----ASHALK
On_3a    ---------- ------KHWQ -------QDF QES--APLCQ ---------- ------GEE- ---SYGGAA- ---------- ---------- ---------- -PAESESSMS EVYSP---AS SS---LSSDS DESGG----- ----LVWPQQ LPPRL---AS TSSSSSP-SP QATA-NTPSQ P-KAFVKIK- ----ASHALK
Dr_3b    ---------- ---------- ---------- ------PQPQ ---------- ------LRD- ----YT-AV- ---------- ---------- ---------- -PGESESSLS EAESP---GS SS---LSSDS DGSGG----- ----LVWPQQ LHPQP---AP T----SP-SH PPGH-GQDLQ P-KAFVKIK- ----ASHALK
Tr_3b    ---------- ------QHL- -------EYQ PERVPMHPCQ P--------- ------SED- ----YHHPA- ---------- ---------- ---------- -QGESESSTS EADSP---DS SS---LSSDS DESGG----- ----LVWPQQ LAPQL---SL A----SP-P- TPP--GAAQQ P-KAFVKIK- ----ASHALK
On_3b    ---------- ------QHM- -------EFQ LERDQAQLCQ A--------- ------TDD- ----YSHAG- ---------- ---------- ---------- -QVESESSMS EADSP---DS SS---LSSDS DESGG----- ----LVWPQQ LPPRL---S- -----LP-ST LAPS-GAPLQ P-KAFVKIK- ----ASHALK
Cm_3     XXXXXXXXXX XXXXXXXXXX XXXXXXXXXX XXXXXXXXXX XXXXXXXXXX XXXXXXXXXX XXXXXXXXXX XXXXXXXXXX XXXXXXXXXX XXXXXXXXXX XXXXXXXXXX XXXXXXXXXX XXXXXXXXXX XXXXXXXXXX XXXXXXXXXX XXXXXXXXXX XXXXXXXXXX XXXXXXXXXX XXXXXXXXXX XXXXXXXXXX
Pm_A     PVMVPVAPAA LVGGGALHGS QRLV--ESD- SEY--SAECE SLFRT-SFGA T--GSSDDD- ---------- ---------- ---------- ----DDDDDR DEEDTDCDAD DAEDA-RGG- GVDTVGPANV AEGGGRAEPA ----LAGSLA SSSSISSSSF FSXXXXXXXX XXXXXXXXXX XXXXXXXXXX XXXXXXXXXX
Lj_A     PVMVPVAPAA VVGGGARHGS QRLV--ESD- SEY--SAECE SLFRT-SFGA T--GSSDDD- ---------- ---------- ---------- ----DDDDDR DEEDTDCDVD DAEDA-RGGG GGDTVGPADV AEGGGRAEPA ----LTGSTA TSSSISSSSF FSSSSSSSSD GPRAGGLGCV PQAQICRIK- ----ASHALK
Pm_B     XXXXXXXXXX XXXXXXXXXX XXXXXXXXXX XXXXXXXXXX XXXXXXXXXX XXXXXXXXXX XXXXXXXNRF EKERRAPAES FRTQKFDDYD RDGDDEEEEE VATDSDKRRG RRRLQREHGF SASDEEEEDD VDTGGRQQSK GLP-LAGRRG GQKAPVVAAP VAARGPEARG GG--GGGVRG QQHAACRIK- ----ASRSLK
Lj_B     ------ARAY VTAVARADS- ------DSER SEY--SAECE SLFHGTVMDT S-----EDE- -MSDHTTNRF GDGETSASEG VPCSEVDDDY RD--DDEEEE VATDSDSDGD AGGFSVSTGS SASDEDEEDD VDNGGRQQSK GVP-LAGRRG GQKAPAVAAP VAARGPEARG S----GGARG QQHAACRIK- ----ASRSLK
Lj_C     PGYPNVYPAA GCPGELLQR- ---------- -----SAIAE M--------- ------SED- ---------- ---------- ---------- -------DAD DADEA----- --DGAAGRLP ADG--SAEP- ---------- ---------- RSS---GSPE GLRGP----- -----CRVSQ QVRAVPSAVK
Lj_D     GGGNGFRGNS FHGNGFRGDG FHT---DGFH ADG-FHADGF PRSGFQGHSF HGVAKLPRGS NGLRGNPMH- AGSAGCLRDG F----LGDGY R-----GDAW RRGDARPQQG KASTGFGPGW GRPGAGVAGP QAGGG----- ------WWGA GPGGLG--VA GGRFN-GPGP QVSVA--HVV PVGPAGLDG- -----PAARR
Pm_D     XXXXXXXXXX XXXXXXXXXX XXXXXXXXXX XXXXXXXXXX XXXXXXXXXX XXXXXXXXXX XXXXXXXXXX XXXXXXXXXX XXXXXXXXXX XXXXXXXXXX XXXXXXXXXX XXXXXXXXXX XXXXXXXXXX XXXXXXXXXX XXXXXXXXXX XXXXXXXXXX XXXXXXXXXX XXXXXXXXXX XXXXXXXXXX XXXXXXXXXX
Hs_2     --------GR PSLV----RQ DAYTRSDSEP SKH--SAECD PRFPSVIPET S-----EGE- -SSDHTTNR- ---------- ---------- ---------- -FGDRESSSS DEEGGAQSRD CD---LALGY VAA-----GH AE--LAW--- -TQE------ -----AP--V S---SGPLLS PVPKLCRIK- ----ASKALK
Md_2     --------SR SKIP----SH NTYAKSESDH SEY--SAECA SLFHSTIVET S-----EDD- -VSDYTANC- ---------- ---------- ---------- -FGDSESGDS DSDGACRSSE SS---LTLDY EEA-----VG SE--LIWPKA IVQQP----- VK---GQ--A A---SKPSHH PVPKICRIK- ----ASKALK
Gg_2     --------AR PKSH----HH GAYAKSESDH SEY--SAECA SLFHSTIAET S-----EGE- -VSDFTANR- ---------- ---------- ---------- -FGDSESSES DSDD--SSNS SS---LALDY DEG-----DE SE--LIWPEG SVRQL----- -----GTAQA S---SKP-LP PVPKICRIK- ----ASKALK
Ac_2     --------AR SKSRH-PFHP GTYAKSESDH SEY--SAECA SLFHSTIAET S-----EGE- -VSDYTANR- ---------- ---------- ---------- -FGDSESSES DSEG------ SS---LTLDY EEEEI---EE HE--LIWPDS SVRQP----- -----TSTQT S---SQP-LP SVPKLCRIK- ----ASKALK
Cpb_2    --------AR SKHHR----H GTYAKSESDH SEY--SAECA SLFHSTIAET S-----EGE- -VSDYTTNC- ---------- ---------- ---------- -FGDSESSES DSEG--SSNS SS---LTLDY DDA-----DE SE--LVWPEA SVRQP----- -----AAVQA S---SKP-LP PMPKICRIK- ----ASKALK
Lc_2     --------TH QAHYRHSAYI RSSARSDSDQ SEY--SAECA SLFHSTIVET S-----EDE- -RSDYTTNR- ---------- ---------- ---------- -FGDSESSES DSDAT-SD-- SS---LTLDY DDT-----DA SE--FVWPEA SS-QP---AA SS---QPAAA SVAPSKQ-AH TEPKLCRIK- ----ASKALK
Lo_2     ---HRPSQYG VPRHPH-PNS YSCLLSESEV SEY--SAECA SLFHSTIAES S-----EGE- -LSDFTANR- ---------- ---------- ---------- -FGDSESSES SSDGS-SDSS LS---LNSE- D---M---ED GE--LVWAQS TV-------- -----GPTAA G---LPLSSR PEPPACRIK- ----ASKALK
Dr_2     SCFSYLESRY PAAP---VSS RHPPRCESEF SEY--SAECA SLFHSTIAAS S-----DGE- -MSDYTTNR- ---------- ---------- ---------- -FGDSESSQG SQTA--SESD SS---LSLDE ED-LLEEEEE DEGGLVWAQA AM-------- -----GTTAA GFS-LQQHHR SESAACRIK- ----ASRALK
Tr_2     -FLHNLNARY PPSPFH-MSR HYPPRCE--- SEY--SAECA SLFHSTIAES S-----EGE- -MSDNTTNR- ---------- ---------- ---------- -FGDSESSQS FLSF--SDSD SS---LSLDE DYQVDNQGEE RN--LVWAED SL-------- -----GPTAA G---LQQLPR PEPSACRIK- ----ASRALK
On_2     -FLHSLNAKY PPAPFH-VTS HYPPRCE--- SEY--SAECA SLFHSTIAES S-----EGE- -MSDNTTNR- ---------- ---------- ---------- -FGDSESSQS FQSF--SDSD SS---LSLYE GEQADSHEEE GS--MVWAEA AL-------- -----GPTAA GRPLKQLPPR PEPAACRIK- ----ASRALK
Cm_2     -----VSFRS RTFENH-FSV YSYARSESDQ SEY--SAECA SLFHSTIVET S-----EDD- -ESDHTTNC- ---------- ---------- ---------- -FGDGELSGG SVSD--TASN SS---LSLDD DDPA------ ----LVWPQG LV------AA N----EPTSS S---CQP-RH SEAQVCRIK- ----ASKALK
Ac_4     ---------- ---------- ---------H LAF--SSDSP NSFCFL--EG F------ER- -SLT------ ---------- ---------- ---------- -ATTSSSS-- -LAKVEATSA IN---LNGD- ---------- -----WILQQ LGDGW----- ------PHT- A--DSTDVPV RRVKLHRSR- ----SFKEFK
Cpb_4    ---------- ---------- ---------Q LAF--STDS- ---CFP--DG S-----QLE- --LQPQRA-- ---------- ---------- ---------- -EGSSASS-- -LSEVD---A VS---LNGD- ---------- -----WRLRQ LGPPRARP-- ------LHAS ATLDSSNLAM ASFKLHRTR- ----SFKELK
Lc_4     ---------- ---------- ---------H RAF--STDCP SSFHSPILEP S-----QTD- --LQNTTAHY ---------- ---------- ---------- -LYGSESS-- -LSEVDATSC TS---LNRE- ---------- -----LIHED RSIARPHLGE ------KFFS NPLDNKNMVN GSIQLYRTK- ----SFKELK
Lo_4     XXXXXXXXXX XXXXXXXXXX XXXXXXXXXX XXXXXXXXXX XXXXXXXXXX XXXXXXXXXX XXXXXXXXXX XXXXXXXXXX XXXXXXXXXX XXXXXXXXXX XXXXXXXXXX XXXXXXXXXX XXXXXXXXXX XXXXXXXXXX XXXXXXXXXX XXXXXXXXXX XXXXXXXXXX XXXXXXXXXX XXXXXXXXXX XXXXXXXXXX
Dr_4     ---------- ---------- ---------Q WRS--SFEI- -SRAGRGSER A------RN- --LVRK---- ---------- ---------- ---------- -EEKHHSSTA SLFHL-SRSQ S----LEGSS H--------- -----SLS-- ---------- --------PL SSPSLSPSPP PSAPLTRSR- ----SFRDLG
Tr_4     ---------- ---------- ---------H WVS--SLDVA QGGLYVSRDE A------VR- --LLSTKG-- ---------- ---------- ---------- -EDKRLSSTA SLFNL-SRSQ S----LEGSC H--------- -----SIS-- ---------- --------PL SSPSFSPSPP SRMPLQRSR- ----SLRDLG
On_4     ---------- ---------- ---------H WVS--SLDVG HGGMGIRKDE G------VR- --LLRAKE-- ---------- ---------- ---------- -EDKRLSSTA SLFNL-SRSQ S----LEGSC H--------- -----SLS-- ---------- --------PL SSPSFSPSPP PRLPLQRSR- ----SLRDLG
Cm_4     XXXXXXXXXX XXXXXXXXXX XXXXXXXXXX XXXXXXXXXX XXXXXXXXXX XXXXXXXXXX XXXXXXXXXX XXXXXXXXXX XXXXXXXXXX XXXXXXXXXX XXXXXXXXXX XXXXXXXXXX XXXXXXXXXX XXXXXXXXXX XXXXXXXXXX XXXXXXXXXX XXXXXXXXXX XXXXXXXXXX XXXXXXXXXX XXXXXXXXXX
Lo_4r    XXXXXXXXXX XXXXXXXXXX XXXXXXXXXX XXXXXXXXXX XXXXXXXXXX XXXXXXXXXX XXXXXXXXXX XXXXXXXXXX XXXXXXXXXX XXXXXXXXXX XXXXXXXXXX XXXXXXXXXX XXXXXXXXXX XXXXXXXXXX XXXXXXXXXX XXXXXXXXXX XXXXXXXXXX XXXXXXXXXX XXXXXXXXXX XXXXXXXXXX
Dr_4r    XXXXXXXXXX XXXXXXXXXX XXXXXXXXXX XXXXXXXXXX XXXXXXXXXX XXXXXXXXXX XXXXXXXXXX XXXXXXXXXX XXXXXXXXXX XXXXXXXXXX XXXXXXXXXX XXXXXXXXXX XXXXXXXXXX XXXXXXXXXX XXXXXXXXXX XXXXXXXXXX XXXXXXXXXX XXXXXXXXXX XXXXXXXXXX XXXXXXXXXX
Bfl      F-------SR GVRGLK---- ----KAGQSR STSR-LYDYD T---SVLGSS S-----EE-- -SSSS----- ---------- ---------- ---------- ---DSEINRS DSESD---SE S--------S DEAGG----- ----LVLA-- ----KAKWP- AVVGTAT--- ---------- ---------- ---------R
Motifs   ---------- ---------- ----****** **motif11d *(ex4/ex5) ********** ********-- ---------- ---------- ---------- -********* ****11e*** ********** *****----- ----***11f ***------- ---------- ------**** ******moti f11g******

                 1810       1820
         ....|....| ....|....| ...
Hs_1     KKILRFRSGS LKLMTTV--- ---
Md_1_    KKILRFRSGS LKLMTTV--- ---
Gg_1     KKILRFRSGS LKLMTTV--- ---
Ac_1_    KKILRFRSGS LKLMTTV--- ---
Cpb_1    KKILRFRSGS LKLMTTV--- ---
Xt_1     KKILRFRSGS LKLMTTV--- ---
Lc_1     KKILRFRSGS LKLMTTV--- ---
Lo_1     KKILRFRSGS LKLMTTV--- ---
Dr_1     KKILRFRSGS LKLMTTV--- ---
On_1     KKILRFRSGS LKLMTTV--- ---
Cm_1     KKSCAFXXXX XXXXXXXXXX XXX
Hs_3     KKILRFRSGS LKVMTTV--- ---
Md_3     KKILRFRSGS LKVMTTV--- ---
Ac_3     KKIMRFRSGS LKVMTTV--- ---
Cpb_3    KKIMRFRSGS LKVMTTV--- ---
Xt_3     KKIMRFHTGS LKVMTTV--- ---
Lc_3     KKIMRFRTGS LKVMTTV--- ---
Lo_3     KKILRFRTGS LKVMTTV--- ---
Dr_3a    KKIMRFRSGS LKLMTTV--- ---
Tr_3a    RKILRFRSGS LKVMTTV--- ---
On_3a    KKILRFRSGS LKVMTTV--- ---
Dr_3b    KKILRFRSGS LKVMTTV--- ---
Tr_3b    KKILRFRTGS LKLMTTV--- ---
On_3b    KKILRFRTGS LKVMTTV--- ---
Cm_3     XXXXXXXXXX XXXXXXXXXX XXX
Pm_A     XXXXXXXXXX XXXXXXXXXX XXX
Lj_A     KKILRCRSGS LKLMTTV--- ---
Pm_B     KKMMHFRSGS LKVITTV--- ---
Lj_B     KKMMHFRSGS LKVITTV--- ---
Lj_C     RKIRQRIADA AKTSTDV--- ---
Lj_D     RALLRCHSVE PALTTTV--- ---
Pm_D     XXXXXXXXXX XXXXXXXXXX XXX
Hs_2     KKIRRFQPTA LKVMTMV--- ---
Md_2     KKIRRFQPAS LKVMTMV--- ---
Gg_2     KKIRRFQPAS LKVMTMV--- ---
Ac_2     KKIRRFQPAS LKVMTMV--- ---
Cpb_2    KKIRRFQPAS LKVMTMV--- ---
Lc_2     KKIRKFQPAS LKVMTMV--- ---
Lo_2     KKIRRFQPAA LKVMTMV--- ---
Dr_2     KKIRRFQPAS LKVMTLV--- ---
Tr_2     KKIRRFQPAS LKVMTLV--- ---
On_2     KKIRRFQPAS LKVMTLV--- ---
Cm_2     KKIRRFQPDS LKIMTMV--- ---
Ac_4     KAMSRSLRG- -SRATK---- ---
Cpb_4    RMVSRSLRPL APKASGASK- ---
Lc_4     KRVSRSMRPF SFKGTSSTK- ---
Lo_4     XXXXXXXXXX XXXXXXXXXX XXX
Dr_4     RKVFGSMRSL SFNKNHKSKK KD-
Tr_4     RKVFGSMRSL SLKRKPSKK- ---
On_4     RKVFGSMRSL SLKQKSSKK- ---
Cm_4     XXXXXXXXXX XXXXXXXXXX XXX
Lo_4r    XXXXXXXXXX XXXXXXXXXX XXX
Dr_4r    XXXXXXXXXX XXXXXXXXXX XXX
Bfl      RRL-SSQDSL SRMFPSCHLV TEL
Motifs   ******PDZ- binding--- ---
```
