# Supplementary material for: Dact genes are chordate specific regulators at the intersection of Wnt and Tgf-β signaling pathways
Source: BMC Evol Biol. 2014 Aug 6;14:157. doi: 10.1186/1471-2148-14-157 (PMC4236578; doi:10.1186/1471-2148-14-157)

Additional File 14. Trimmed alignment of Dact protein sequences.

Alignment of protein sequences for all Dact genes used in the phylogenetic analysis after removal of poorly aligned columns. This aligment was used for the reconstruction of the phylogenetic trees. Gaps are indicated by ‘-‘, missing sequences by ‘X’.

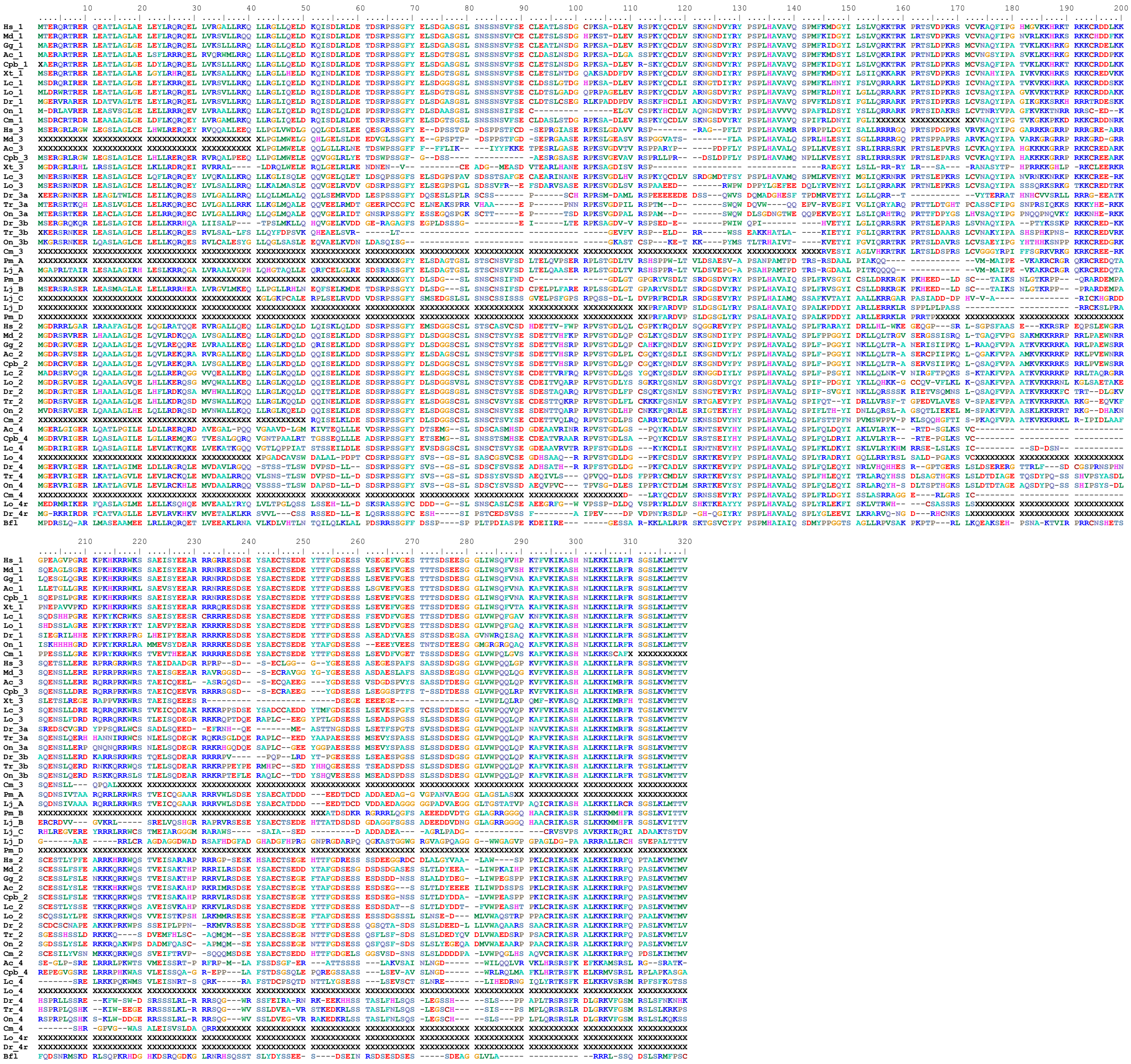

Supplement: Additional file 14 — Trimmed alignment of Dact protein sequences. [file 1471-2148-14-157-S14.pdf]
